# MeDeMSA care study protocol: developing personalized best medical care with integrated mobile palliative and telemedicine support for individuals with multiple system atrophy

**DOI:** 10.1007/s00702-025-02933-z

**Published:** 2025-05-24

**Authors:** Alessandra Fanciulli, Bianca Caliò, Svenja Schmidt, Georg Goebel, Fabian Leys, Karoline Radl, Caroline Breitegger, Marjan Arvandi, Alexa Blum, Christoph Gabl, Oliver Galvan, Achim Herms, Birgit Högl, Bernhard Holzner, Frank Jagusch, Stefan Kiechl, Andrea Knoflach-Gabis, Mariella Koegl, Igor Kuchin, Stefan Lorenzl, Werner Poewe, Sinikka Prajczer, Gerhard Rumpold, Andreas Schlager, Anette Schrag, Klaus Seppi, Uwe Siebert, Gudrun Schönherr, Gaby Sroczynski, Martina Schmidhuber, Petra Schwingenschuh, Beate Jahn, Florian Krismer, Gregor K. Wenning

**Affiliations:** 1 Department of Neurology, Medical University of Innsbruck, Anichstrasse 35, 6020 Innsbruck, Austria; 2 EPICENTER - Institute of Clinical Epidemiology, Public Health, Health Economics, Medical Statistics and Informatics, Medical University of Innsbruck, Innsbruck, Austria; 3 Department of Health Care Ethics, University of Graz, Graz, Austria; 4 Institute of Public Health, Medical Decision Making and Health Technology Assessment, Department of Public Health, Health Services Research and Health Technology Assessment, UMIT TIROL-University for Health Sciences and Technology, Hall in Tirol, Austria; 5 Department of Psychiatry, Psychotherapy, Psychosomatics, and Medical Psychology, University Clinic of Psychiatry II, Innsbruck Medical University, Innsbruck, Austria; 6 Tyrolean Regional Institute for Integrated Palliative and Hospice Care, Tiroler Hospiz Gemeinschaft, Hall in Tirol, Austria; 7 Department for Hearing, Speech and Voice Disorders, Medical University of Innsbruck, Innsbruck, Austria; 8 Neuro-Urology Unit, Department of Urology, Medical University of Innsbruck, Innsbruck, Austria; 9 University Clinic of Psychiatry I, Innsbruck Medical University, Innsbruck, Austria; 10 Department of Neurology, Medical University of Graz, Graz, Austria; 11 Institute of Palliative Care, Paracelsus Medical University, Salzburg, Austria; 12 Department of Anesthesiology and Intensive Care Medicine, Medical University of Innsbruck, Innsbruck, Austria; 13 Department of Neurology, University College London, London, UK; 14 Department of Neurology, Provincial Hospital of Kufstein, Kufstein, Austria; 15 Institute for Technology Assessment and Department of Radiology, Massachusetts General Hospital, Harvard Medical School, Boston, MA USA; 16 Center for Health Decision Science and Departments of Epidemiology and Health Policy & Management, Harvard T.H. Chan School of Public Health, Boston, MA USA

**Keywords:** Multiple system atrophy, Neuro-rehabilitation, Advance care planning, Telemedicine, Palliative medicine, Caregivers

## Abstract

Multiple system atrophy (MSA) represents a management challenge due to its variable clinical presentation, and lack of established multidisciplinary care protocols. In advanced stages, physical barriers often prevent access to specialized care, leaving patients and caregivers to face complications and fear alone. This 18-month, monocentric, open-label study evaluates the impact of a personalized, multidisciplinary treatment, integrating mobile palliative care, on the quality of life (QoL, measured by EQ-5D-5L indices) of MSA individuals compared to a matched historical European MSA cohort. Forty-six participants will undergo baseline clinical, psychological, and neuro-rehabilitation assessments, along with an online interview to identify individual healthcare preferences. These assessments will guide personalized therapeutic plans, including palliative care, self-directed physio-, speech, and occupational exercises, based on operational protocols informed by evidence and good clinical practice. Six-, 12-, and 18-month follow-ups will reassess needs and adapt plans to address disease progression and changing preferences, ensuring continuous personalization. Repeated interviews at month-12, phone-calls and satisfaction surveys at month-1, 7, 13, and 18 will monitor compliance, identify barriers, and gather feedback. A subgroup of 23 participants will be block-wise randomized to additionally receive monthly and on-demand telemedicine visits for tailored support. Informal caregivers will join an 18-month observational study assessing their QoL and burden through repeated evaluations, offering insights into evolving challenges. We expect that multidisciplinary, patient-centered care with integrated mobile palliative and telemedicine support helps to warrant continuity of care and improve the QoL of MSA individuals throughout the disease course. Its acceptance, safety, and cost-effectiveness will be also assessed.

**Supplementary Information:**

The online version contains supplementary material available at 10.1007/s00702-025-02933-z.

## Introduction

Multiple system atrophy (MSA) is a rare, fatal neurodegenerative disease characterized by progressive autonomic failure, parkinsonian, cerebellar, and pyramidal features in varying combinations (Fanciulli and Wenning 2015). Life expectancy averages less than ten years from symptom onset (Wenning et al. 2013), with common causes of death including urosepsis, aspiration pneumonia, and sudden death during sleep.

Despite existing clinical guidelines for managing key MSA symptoms (Calandra-Buonaura et al. 2021; Brignole et al. 2018; Gibbons et al. 2017; Cortelli et al. 2019; B. Blok 2022; Jordan et al. 2019; Randerath et al. 2017), comprehensive, personalized, multidisciplinary care protocols remain lacking. This poses a challenge for both neurologists and allied health professionals—including psychologists, physiotherapists, speech and occupational therapists—who currently lack regular interdisciplinary collaboration, despite the potential value of concerted pharmacological and non-pharmacological interventions for mitigating MSA symptomatic burden.

Medical tests play a critical role in guiding diagnosis, treatment decisions, and clinical trial enrollment, ultimately affecting patient outcomes. The benefits, risks, and costs of different test-treatment strategies can be systematically evaluated using decision-analytic models (Siebert 2003; Caro et al. 2012), which rely on trial data, registries, literature, and other sources (Siebert 2003). However, no studies to date systematically assessed the benefit-harm balance or cost-effectiveness of MSA test-treatment strategies. Additionally, data on the annual healthcare costs of MSA remain limited (Winter et al. 2011; McCrone et al. 2011), altogether creating a barrier to informed health policy decisions aimed at optimizing care and improving patients’ quality of life (QoL).

Early palliative care has been shown to alleviate the symptoms burden while reducing healthcare costs due to avoidable hospital admissions in chronic neurological conditions, including MSA (Hepgul et al. 2020). Multidisciplinary palliative care programs have been developed in the past for individuals with amyotrophic lateral sclerosis (https://www.hospiz-tirol.at/begleitung/als-netzwerk). However, integration between neurology and specialized palliative care for individuals with MSA services remains limited to date (van Vliet et al. 2016; Oliver et al. 2024; McKenzie et al. 2022).

Beyond the affected individuals, MSA places a significant burden on informal caregivers, i.e., family members or friends who provide unpaid support to those with chronic illnesses (Roth et al. 2015). Caregiver burden encompasses physical, psychological, financial, and social challenges (Zarit et al. 1980), and is influenced by caregiving intensity and supervision hours, with female caregivers often experiencing greater strain (Miyashita et al. 2011; Campese et al. 2024; Shurer et al. 2024; Lindt et al. 2020). Considering that MSA typically results in moderate-to-severe disability within five to six years of onset (Krismer et al. 2024), early familial involvement is essential (Wiblin et al. 2017; Gallop et al. 2023). However, research on the QoL of MSA caregivers remains limited, and support services remain insufficient globally.

The COVID-19 pandemic further exacerbated these challenges by disrupting in-person healthcare services, and increasing the burden on individuals with chronic neurological conditions (Russo et al. 2021; Rakusa et al. 2024). At the same time, the pandemic crisis accelerated the adoption of digital health solutions (Fanciulli et al. 2023). In Parkinson’s disease (PD), telemedicine consultations proved effective for “practicing medicine at a distance” and alleviating the psychological burden of patients and their caregivers (Adams et al. 2020; Fanciulli et al. 2023). Telerehabilitation also demonstrated benefits on parkinsonian motor and non-motor symptoms (Morris et al. 2021; Tamplin et al. 2021; Vellata et al. 2021). For people with MSA, the benefits of remote healthcare may extend beyond the pandemic horizon (Russo et al. 2021), particularly if overcoming physical and geographical barriers enables sustained healthcare delivery at advanced disease stages, when identifying life-threatening complications supports coping (Borders et al. 2021).

Building on the multidisciplinary expertise in MSA care at the Medical University of Innsbruck and its national and international partners, the Medical Decision Making in Multiple System Atrophy (MeDeMSA) Care study has been launched. It aims to evaluate whether a personalized, multidisciplinary care model, integrating mobile palliative care and telemedicine support, can attenuate QoL decline in prospectively recruited individuals with MSA by alleviating the symptomatic burden, ensuring continuity of care, and prioritizing individual healthcare preferences. QoL changes over 18 months are captured with the European QoL Questionnaire – 5 Dimensions – 5 Levels [EQ-5D-5L, (Rabin and de Charro 2001; Feng et al. 2021)] and compared to a matched historical European MSA cohort that received standard care [European MSA Study GroupEMSA-SG, cohort (Wenning et al. 2013)].

Secondary objectives of the MeDeMSA Care study are to assess the acceptance, safety, and cost effectiveness of such an integrated healthcare model, and are detailed in Table 1, along with their respective outcome measures. We here aim at sharing the MeDeMSA Care study design and operational protocols.

**Table 1:** Primary and secondary objectives of the MeDeMSA Care Study (left) and respective outcome measures (right)

	Objective	Outcome measure
Quality of life	*Primary*	
	Impact of a multidisciplinary, personalized treatment plan including mobile palliative care (as needed) on the baseline to 18-months change in QoL of prospectively recruited MSA individuals, compared to sex-, age- (± 5 years), and disease duration- (± 12 months) matched MSA individuals from the historical EMSA-SG cohort^a^	- Baseline to 18-months EQ-5D-5L score change
	*Secondary*	
	Impact of a multidisciplinary, personalized treatment plan including mobile palliative care (as needed) on the baseline to 6- and 12-months change in QoL of prospectively recruited MSA individuals, compared to sex-, age- (± 5 years), and disease duration-(± 12 months) matched MSA individuals from the historical EMSA-SG cohort^a^	- Baseline to 6- and 12-months EQ-5D-5L score change - Baseline to 6-, 12- and 18-months changes in the MSA-QoL total score and subscores
Clinical features	Impact of a multidisciplinary, personalized treatment plan including mobile palliative care (as needed) on the baseline to 6-, 12- and 18-months progression of motor, non-motor symptoms and time to clinical milestones of prospectively recruited MSA individuals, compared to sex-, age- (± 5 years), and disease duration- (± 12 months) matched MSA individuals from the historical EMSA-SG cohort^a^	- Baseline to 6-, 12- and 18-months change in motor and non-motor scales (total and subscores, including video-based, rater-blinded assessment of the UMSARS motor subscore and Hoehn & Yahr stage) - Time to clinical milestones (i.e. falls at least once a day, feeding by nasogastric tube or gastrostomy, unintelligible speech, indwelling catheter, wheelchair dependency)
	Baseline to 18-months therapeutic needs of individuals with early versus advanced^b^ MSA	- Medical and neuro-rehabilitation interventional needs over the 18-months study period (see MeDeMSA Operational Protocol in Supplementary Material 1)
	To compare the number of medical complications in individuals with early versus advanced^b^ MSA over the 18-months study period	- Number of medical complications (i.e., falls with or w/o injuries, urinary tract infections, choking, aspiration pneumonia, hospitalizations, death and others) over the 18-months study period
Psychological aspects	To compare the individual healthcare preferences at baseline and after 12 months in individuals with early versus advanced^b^ MSA	- Baseline and 12-months semi-structured online interview - Baseline to 12-months APIS change
	To compare the baseline to 18-months psychological counselling needs in individuals with early versus advanced^b^ MSA	- Psychological counselling needs of individuals with MSA over the 18-months study period (see modular intervention system in the MeDeMSA Operational Protocol, Supplementary Material 1)
	To compare the month 1, 7, 13, and final individual satisfaction with the personalized treatment plan in individuals with early versus advanced^b^ MSA	- Month 1 to 7, 13 and 18 SAPS change referred to the overall individualized treatment plan
Palliative care	To assess the feasibility and barriers to mobile palliative care in individuals with advanced^b^ MSA	- Structured documentation of the palliative intervention - Individual satisfaction with single mobile palliative interventions (IPOS)
HTA	To perform a health technology assessment of an 18-months multidisciplinary, personalized treatment plan including mobile palliative care and telemedicine interventions in individuals with early versus advanced^b^ MSA	- Changes in the EQ-5D-5L, MSA-QoL, motor and non-motor scales, time to clinical milestones, number of medical complications, and satisfaction indices over the 18-months study period - Single-intervention and cumulative healthcare costs over the 18-months study period
Caregivers	To compare the caregiver-burden in informal caregivers of individuals with early versus advanced^b^ MSA over 18-months study period	- Baseline to 6-, 12- and 18-months change in the EQ-5D-5L score of informal caregivers of prospectively recruited MSA individuals - Baseline to 6-, 12- and 18-months change in PQoL Carers score and other caregiver-burden indicators in informal caregivers of prospectively recruited MSA individuals - Baseline and 12-months semi-structured online interview
Telemedicine	To compare the feasibility and barriers to multidisciplinary telemedicine visits in individuals with early versus advanced^b^ MSA	- Individual satisfaction with the single telemedicine interventions, i.e. neurological consultations, psychological counselling, physio-, speech and occupational therapy (online forms, including numeric rating scales and open-end questions) - Healthcare professional’s satisfaction with the single telemedicine consultations (numeric rating scales and open-end questions)
	To assess whether monthly multidisciplinary telemedicine visits in addition to a multidisciplinary, personalized treatment plan including mobile palliative care - Attenuate the baseline to 6-, 12- and 18-months QoL worsening - Attenuate the progression of motor and non-motor MSA symptoms, including time to clinical milestones in prospectively recruited MSA individuals compared to sex-, age- (± 5 years), and disease duration-(± 12 months) matched MSA individuals from the historical EMSA-SG cohort^a^ and to prospectively recruited MSA individuals receiving in-person visits and mobile palliative care (as needed) only	- Baseline to 6- and 12-months EQ-5D-5L score change - Baseline to 6-, 12- and 18-months changes in the MSA-QoL total score and subscores - Baseline to 6-, 12- and 18-months change in motor and non-motor scales (total and subscores, including video-based, rater-blinded assessment of the UMSARS motor subscore and Hoehn & Yahr stage) - Time to clinical milestones (i.e., falls at least once a day, feeding by nasogastric tube or gastrostomy, unintelligible speech, indwelling catheter, wheelchair dependency)
	To assess whether monthly multidisciplinary telemedicine visits in addition to a multidisciplinary, personalized treatment plan including mobile palliative care - Reduce the number of medical complications - Reduce the caregiver burden - Provide an advantage in terms of benefit-harm and cost-effectiveness tradeoffs - Increase the individual satisfaction with the personalized treatment plan over the 18-months study period in prospectively recruited MSA individuals compared to prospectively recruited MSA individuals receiving in-person visits and mobile palliative care (as needed) only	- Number of medical complications (i.e., falls with or w/o injuries, urinary tract infections, choking, aspiration pneumonia, hospitalizations, death and others) over the 18-months study period - Baseline to 6-, 12- and 18-months change in the EQ-5D-5L score of informal caregivers of prospectively recruited MSA individuals - Baseline to 6-, 12- and 18-months change in PQoL Carers score and other caregiver-burden indicators in informal caregivers of prospectively recruited MSA individuals - Baseline and 12-months semi-structured online interview - Single-intervention and cumulative healthcare costs over the 18-months study period - Month 1 to 7, 13 and 18 SAPS change

^a^ In case such matching will not be possible due to unavailable controls from the historical EMSA-SG cohort, a matching between individuals below/above 60 years of age and below/above 3 years of disease duration will be applied

^b^ Advanced MSA: wheel-chair bound at the time of recruitment

*APIS* autonomy preference index survey; *EMSA-SG* European MSA Study Group; *EQ-5D-5L*: European Quality of Life Questionnaire – 5 Dimensions – 5 Levels; *IPOS*: integrated palliative outcome scale; *QoL* quality of life; *MSA-QoL* MSA quality of life questionnaire; *PQoL* carers: carers quality of life questionnaire for Parkinsonism; *SAPS* short assessment of patient satisfaction; *UMSARS* unified multiple system atrophy rating scale *w/o* without

## Methods

### Study design

This 18-month, monocentric, open-label study evaluates the impact of multidisciplinary, personalized best medical care, including mobile palliative and telemedicine support on the EQ-5D-5L score change of prospectively recruited MSA individuals compared to a sex-, age- and disease-duration matched historical European MSA cohort that received standard care [EMSA-SG cohort, (Wenning et al. 2013)], and whose data is stored at the Medical University of Innsbruck. The EMSA-SG cohort included individuals with MSA from ten countries, who underwent in-person assessments every six months over a 24-month period, showed largely comparable clinical-demographic characteristics across countries, but did not regularly receive treatment for MSA non-motor symptoms, neurorehabilitation, psychological counselling or palliative care offer (Kollensperger et al. 2010).

The study also assesses the participants'motor progression over 18 months using the Unified MSA Rating Scale (UMSARS) Part II through blinded video evaluations. Their progression is then compared to the motor progression curves of matched individuals from the EMSA-SG cohort.

**Fig. 1:**
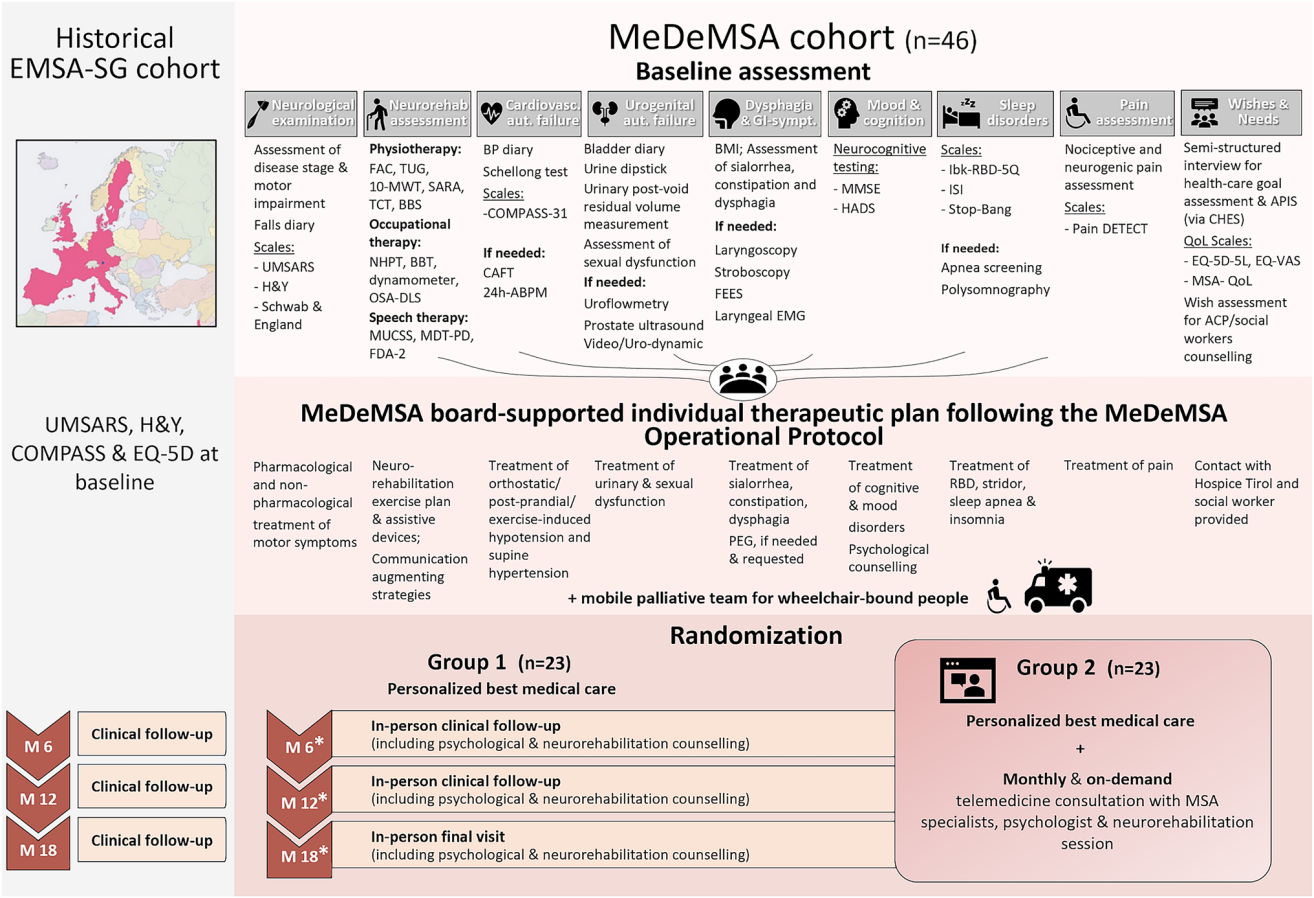
MeDeMSA Care study design. * In the event that the enrolled subjects are for legitimate reasons unable to travel to hospital, they will be offered the opportunity to complete the scheduled on-site assessments of months 6, 12, and 18 using the CHES online platform. *10MWT* 10 m walking test; *24-ABPM* 24-h ambulatory blood pressure monitoring; *ACP* advance care planning; *APIS* autonomy preference index scale; *BBS* berg balance scale; *BBT* box and blocks test; *BMI* body mass index; *BP* blood pressure; *CAFT* Cardiovascular autonomic function tests; *CHES* computer-based health evaluation system; *COMPASS* composite autonomic symptom score; *EMSA- SG* European Multiple System Atrophy-Study Group; *EQ-5D-5L* European Quality of Life- 5 Dimensions- 5 Levels; *EQ-VAS* European quality of life- visual analogue scale; *FAC* functional ambulation categories; *FDA-2* Frenchay Dysarthria Assessment; *FEES* fiberoptic endoscopic evaluation of swallowing; *HADS* hospital anxiety and depression scale; *H&Y* Hoehn and Yahr Scale; *Ibk-RBD-5Q* Innsbruck REM sleep behavior inventory- 5 questions; *ISI* insomnia severity index; Laryngeal EMG laryngeal electromyography; *MDT-PD*: Munich Dysphagia Test- Parkinson Disease; *MMSE* mini mental state examination; *MSA- QoL* multiple system atrophy- quality of life; *MUCSS* Munich- Copenhagen Swallowing Screen; *OSA-DLS* occupational self-assessment- daily living scale; NHPT nine hole peg test; *PEG* percutaneous endoscopic gastrostomy; *QoL* quality of life; *RBD* REM sleep Behavior Disorder; *SARA* scale of the assessment and rating of ataxia; *TCT* trunk control test; *TUG* timed up and go test; UMSARS: unified multiple system atrophy rating scale

An overview of the study design is provided in Fig. 1, and the detailed MeDeMSA Care Visit Plan is reported in Table 2. After providing written consent, participants complete a home bladder and blood pressure (BP) diary for up to 72 h prior to the baseline visit, as well as a falls protocol referring to the month before the visit. During the baseline visit, participants undergo an in-person multidisciplinary assessment with the MeDeMSA Care study team. Following recommendations for PD settings (Bloem et al. 2021), the MeDeMSA Care team is composed of medical, neurorehabilitation, psychology, and palliative healthcare professionals with expertise in MSA care and is designed to meet the care needs that individuals with MSA may develop over the disease course. Baseline assessments include clinical, psychological, and neuro-rehabilitation evaluations, along with an online semi-structured interview on the individual healthcare preferences by the MeDeMSA team in Graz via a telemedicine platform currently used in the Tirol Kliniken [Computer-based Health Evaluation System, CHES, (Holzner et al. 2012)]. Additional tests, such as cardiovascular autonomic, sleep, urodynamic, or swallowing studies, are scheduled as needed during or shortly after the baseline visit. If not performed in the six months prior to the baseline visit, blood tests are conducted to check for undetected comorbidities (i.e., liver or renal failure) that could affect MSA medication dosages or require specific treatment (i.e., anemia, iron or vitamin deficiency). Following the baseline assessments, an individualized treatment plan is prepared using the MeDeMSA Care Operational Protocol (see Supplementary Material 1), which includes pharmacological and non-pharmacological measures, mobile palliative care for wheelchair-bound individuals, and guidance for self-practiced physiotherapy, occupational therapy, and speech therapy exercises (see Supplementary Materials 2, 3 and 4). The MeDeMSA Care Operational Protocol was developed by the MeDeMSA Care multidisciplinary study team based on available guidelines, consensus recommendations, scientific evidence and principles of good clinical practice. The protocol follows a “screen > diagnose > treat  with first-, second-, and third-line options” structure, designed to minimize the burden on recruited individuals and optimize healthcare resource allocation. Additionally, the treatment strategies are tailored to accommodate individual preferences, including the choice between pharmacological and non-pharmacological treatments, as well as among different types of pharmacological options or between tight symptoms’ control versus simplified medication schedules.

**Table 2:**
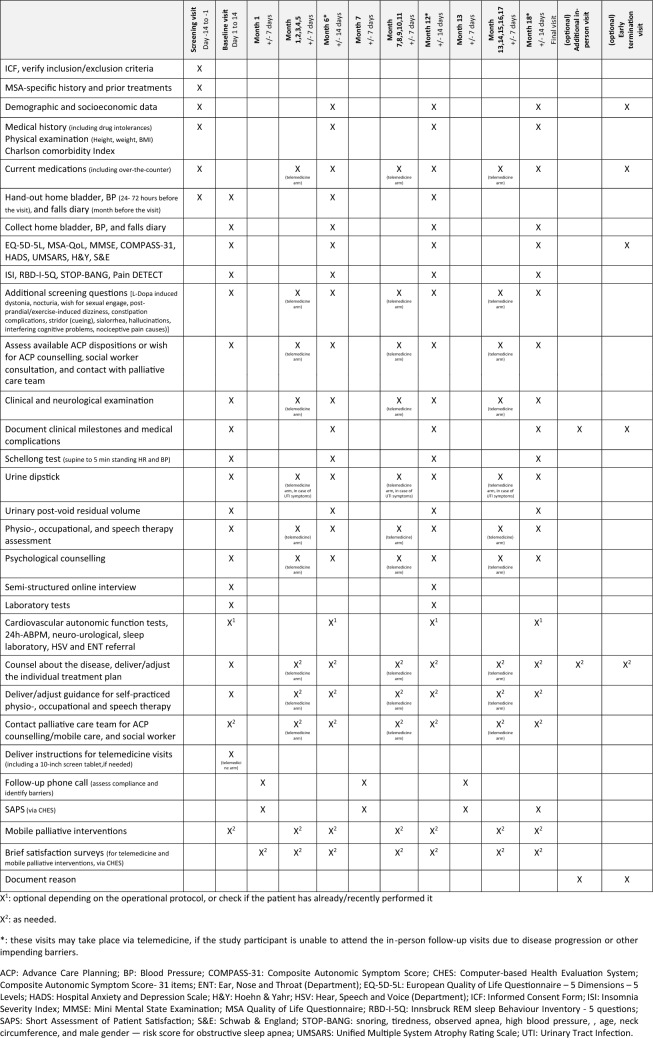
MeDeMSA Care Patient Visit Plan

During follow-up in-person visits at 6-, 12-, and 18-months, the therapeutic needs are reassessed and the treatment plan is adapted as required. If subjects cannot travel to the center, they are offered the possibility to complete the scheduled on-site assessments via the CHES online platform. At month-12, the semi-structured interview is repeated to evaluate changes in healthcare preferences due to disease progression. Follow-up calls are scheduled one month after each in-person visit (at months 1, 7, and 13) to verify treatment compliance and address barriers. Short Assessment of Patient Satisfaction (SAPS) surveys are administered online at months 1, 7, 13, and 18 to evaluate the overall individual satisfaction with the treatment plan. In case additional in-person visits are scheduled, the reason for such visits and eventual therapeutic adaptations are documented.

Upon completion of the baseline assessment, 23 patients are block-wise randomized to additionally receive monthly and on-demand telemedicine visits for neurological, psychological, and neurorehabilitation support via CHES. If the individuals randomized to receive additional telemedicine visits own a tablet or computer with a ≥ 10-inch display, they may use it to enhance digital comfort. Otherwise, a CE-certified tablet (e.g., SAMSUNG Tab A7 T505 LTE 32 GB) is provided. The MeDeMSA Care team members are asked to rate telemedicine feasibility and potential barriers beforehand. After each telemedicine visit, healthcare providers give feedback on how the visit was conducted, document any challenges encountered, and note the adopted strategies to solve them. Brief patient satisfaction surveys follow telemedicine and mobile palliative care interventions: these include a 0–10 rating scale for the overall satisfaction with the intervention and open-ended questions about its advantages and disadvantages. The MeDeMSA Care team holds regular meetings every six to eight weeks to review changes in the care needs of recruited individuals with MSA and coordinate additional support as required.

Informal caregivers of the recruited individuals with MSA are invited to participate in an 18-month observational study, with QoL and caregiver burden assessed at baseline, 6-, 12-, and 18-months using a semi-structured interview and standardized questionnaires, including the EQ-5D-5L and PQoL Carers (Carers Quality of Life Questionnaire for Parkinsonism) (Pillas, Selai et al. 2016). The caregiver visit plan is reported in Table 3.

**Table 3:**
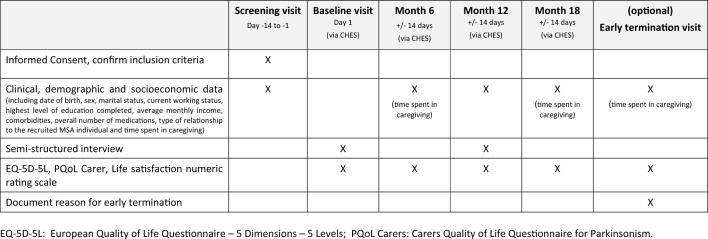
MeDeMSA Care Caregiver Visit Plan

### Population

We plan to recruit 46 individuals, who fulfill all the inclusion criteria listed below:

i. Age ≥ 30 years at the time of consent;
ii. Diagnosed with clinically probable OR clinically established MSA according to the current criteria of the International Parkinson Disease and Movement Disorder Society (Wenning et al. 2022);
iii. Life-expectancy of at least 24 months as assessed by the investigator at the time of consent;
iv. Understands and agrees to comply with the study procedures and provides written informed consent (Note: a legal representative may NOT provide consent on behalf of the subject);
v. Signed and dated informed consent document;
vi. Fluency in German;
vii. If unable to walk or stand without assistance/support at the time of consent, the participant must live in Tyrol or in another Austrian region with available mobile palliative care.

Candidates are excluded from the study if they meet any of the following conditions:

i. Participation in an interventional clinical study at screening and throughout the study that would interfere with the MeDeMSA Care personalized treatment plan or would not permit telemedicine or mobile palliative care;
ii. Charlson Comorbidity Index > 4 at the time of consent;
iii. Other major underlying medical conditions that may confound interpretation of study results as assessed by the investigator.

We further plan to recruit up to 46 informal caregivers of individuals with MSA participating in the MeDeMSA Care trial, provided they meet the following inclusion criteria:

i. Informal caregiver (i.e., a person not receiving payment for caregiving, except for care allowances and tax return schemes available in Austria) of an individual with MSA recruited in the present study;
ii. Life-expectancy of at least 24 months, as assessed by the investigator at the time of consent;
iii. Aged 18 or older at the time of consent;
iv. Understands and agrees to provide information as outlined in the study protocol and to engage in semi-structured online interviews;
v. Provides signed and dated written informed consent;
vi. Full legal capacity;
vii. Fluency in German.

Screening failures, i.e., patients who do not meet eligibility criteria at the time of screening, may be eligible for rescreening at a later time point. Rescreening is acceptable, for example, if individuals in the meantime conclude their participation in other interventional trials or if individuals previously not willing to engage in palliative care or telemedicine interventions change their opinion.

## Statistics

### Sample size calculation

The sample size for the present study has been estimated based on data of the EMSA-SG natural history study (Wenning et al. 2013). In this historical cohort, individuals with MSA showed an average EQ-5D score at baseline of 0.35 (95% CI 0.27–0.42), which worsened to 0.17 (95% CI 0.06–0.28) over an observational period of 18 months under standard care (Wenning et al. 2013). In other chronic medical conditions, treatment is considered to be positively associated with QoL, if the EQ-5D score change in the interventional group averages 0.08 points above that of the control group at follow-up (Kwakkenbos et al. 2013). If effective, we therefore estimate the MeDeMSA Care program to limit the baseline to 18 months EQ-5D worsening to 0.25 (± 0.25 standard deviation, SD) in the MSA individuals receiving personalized best medical care with integrated mobile palliative and telemedicine support, as needed. With an assumed significance level of 5% and a power of 80%, 46 individuals with MSA should therefore enroll in the trial to observe an effect of the personalized multidisciplinary care program compared to the standard care offered to the historical MSA cohort, including an expected drop-out rate of 10% over the study course.

### Randomization

To evaluate the feasibility of healthcare provision via telemedicine in MSA settings, recruited individuals with MSA are 1:1 block-randomized to receive either personalized best medical care with integrated mobile palliative interventions as needed (n = 23) OR personalized best medical care (including on demand mobile palliative interventions) PLUS telemedicine (n = 23). The randomization has a 2-strand stratification that accounts for the early (i.e., able to walk) versus advanced (i.e., wheel-chair bound) stage of MSA at the time of recruitment. The randomization sequence is kept concealed to the recruiting doctor until the baseline assessment is completed.

### Analysis sets and subgroups

For the primary objective (see Table 1), we will compare the prospectively recruited MeDeMSA Care cohort with data from the historical EMSA-SG cohort (Wenning et al. 2013), matched by age (± 5 years), sex, and disease duration (± 12 months). Disease duration is defined as the time from the onset of motor symptoms or selected autonomic features, including orthostatic hypotension, urinary urge incontinence, or incomplete bladder emptying. If exact matching is not possible due to unavailable controls, an alternative matching approach by categorizing individuals into subgroups based on age (< 60 versus ≥ 60 years) and disease duration (< 3 vs. ≥ 3 years) will be applied. To better characterize the MeDeMSA Care cohort and, secondarily, enable detailed comparisons between personalized best medical care with and without telemedicine, the EQ-5D-5L tool has been chosen for the present study (Janssen et al. 2013; Feng et al. 2021). This offers five response levels: no/slight/moderate/severe/extreme problems. For comparisons with the historical cohort, which used the EQ-5D-3L with three response levels (no/moderate/extreme problems), EQ-5D-5L responses will be grouped post-hoc and consistently applied across assessments (van Hout et al. 2012).

For secondary objectives (see Table 2), the MeDeMSA Care cohort will be divided into early versus advanced MSA (i.e., wheelchair-bound at the time of recruitment), and further sub-grouped by sex (male versus female), MSA phenotype [parkinsonian versus cerebellar (Wenning et al. 2022)], age (< 60 versus ≥ 60 years), and disease duration (< 3 vs. ≥ 3 years) for exploratory purposes.

To explore the impact of telemedicine on various outcomes (QoL, symptom progression, complications, caregiver burden, costs, and individual satisfaction with the personalized treatment plan), exploratory comparisons will be made between the subgroup of individuals randomized to receive personalized best medical care and individuals receiving personalized best medical care PLUS monthly and on-demand telemedicine consultations, as well as between each of these subgroups and the historical EMSA-SG cohort.

The individual healthcare preferences, psychological counselling needs, as well as the feasibility and barriers to mobile palliative care in MSA settings will be analyzed in a descriptive way. Subgroup analyses will be performed depending on the available sample size for the different subgroups (i.e., sex, MSA phenotype, younger versus older age, and shorter versus longer disease duration) upon study completion.

### Data analysis

The distribution of quantitative data will be assessed using the Shapiro–Wilk test. Quantitative variables will be reported as mean (± SD) for normally distributed data or median (interquartile range, IQR) for non-normally distributed data, and qualitative variables as counts (percentages). Qualitative variables will be compared using Pearson’s Χ^2^ test (or Fisher’s exact test, if n < 5) and quantitative variables using the Mann–Whitney U or t-test, depending on the data distribution. A Benjamini–Hochberg correction will be applied for multiple tests, whenever appropriate. Binary logistic regression with a receiver operating characteristics (ROC) area under the curve (AUC) calculation will follow univariate analysis when applicable.

Changes in EQ-5D, motor, and non-motor scores between the MeDeMSA Care cohort and age-, sex-, and disease duration-matched historical EMSA-SG individuals will be evaluated with ANOVA for repeated measurements or linear mixed models, as most appropriate. Comparisons between randomization groups and the historical cohort will be run on a descriptive basis, with post-hoc tests adjusting for type-1 error based on ANOVA results.

QoL and caregiver burden changes in caregivers of MSA individuals receiving personalized best medical care with or without telemedicine will be analysed with ANOVA or linear mixed models, adjusted for the disease stage of the cared-for individual with MSA. Correlation analyses will assess the relationship between the caregivers’ QoL and burden with the disease characteristics (e.g., symptom constellation and severity) of the cared-for individual with MSA.

Statistical analysis will be performed with SPSS version 30.0.0 or future releases. P values below 0.05 will be considered statistically significant, with 95% confidence intervals considered where appropriate.

### HTA-supported medical decision making in MSA

In parallel to the clinical study, we aim to develop decision-analytic models for evaluating MSA test and treatment strategies. We will create a model framework and conduct a literature search to inform decision-analytic models, which simulate MSA disease progression, diagnostic work-up, different treatment pathway, health states and events (Roberts et al. 2012; Siebert et al. 2012). The model parameters will be informed by QoL data from the historical EMSA-SG, the MeDeMSA Care cohort and substantiated by a literature search. The decision-analytic model design, analyses and reporting will follow the international methods guidelines of the ISPOR-SMDM Modeling Good Research Practices Task Force, (Caro et al. 2012), key principles of health technology assessment (HTA) and the CHEERS 2022 reporting guidelines for economic evaluations (Drummond et al. 2008; Husereau et al. 2022). Our model will evaluate test-treatment strategies across the remaining lifetime of recruited individuals with MSA, assessing benefits (e.g., increase in life expectancy, quality-adjusted life expectancy), harms (e.g., complications, adverse events), and costs (e.g., diagnostic procedures, treatment, travels), and trade-offs such as harm-benefit and cost-effectiveness ratios. The evaluation will include both a healthcare system perspective and a societal perspective.

## Trial time plan and regulatory aspects

The MeDeMSA Care study commenced in April 2023 and is planned to last five years, with an expected active recruitment phase of 27 months. Following a six months preparatory phase, the MeDeMSA Care Study protocol was approved by the Ethical Committee of the Medical University of Innsbruck (EK No.: 1225/2023) and Medical Director of the Tirol Kliniken in September 2023, and started recruiting in October 2023. The study adheres to the principles of the Declaration of Helsinki, the current European Data Protection Regulation, and the Austrian Agency for Scientific Integrity. Monitoring visits will be carried out by the Innsbruck Competence Center for Clinical Studies in due course.

By the time of the manuscript revision (March 2025), 23 individuals with MSA and 20 informal MSA caregivers have been actively recruited. Regular MeDeMSA Care trial status updates are released on ClinicalTrials.gov (NCT06072105).

## Discussion

At early disease stages, individuals with MSA are usually able to attend specialized clinics, where healthcare provision focuses on symptom control and maintaining autonomy in daily life. At advanced stages, healthcare goals shift towards maximizing the QoL and preventing medical complications. However, by the wheelchair-bound stage, physical and organizational barriers often prevent access to specialized MSA care. Physicians frequently lose track of individuals with advanced MSA, who face life-threatening complications and receive limited support. Beyond mobility and communication problems, the loss of “connection to others” was indeed found to most heavily impact QoL in people with advanced parkinsonism (Strupp et al. 2018; Wiblin et al. 2017). Palliative care plays a crucial role in addressing the physical, psychosocial, and spiritual needs of individuals with life-limiting illnesses (Jasemi et al. 2017). Tailored to cultural and educational backgrounds, it involves mobile teams, hospices, and family caregivers in a holistic approach to care (Breitegger et al. 2024). A key component is advance care planning, which enables individuals to express their healthcare preferences and make healthcare decisions in anticipation of a time when they may no longer be able to express their preferences (Breitegger et al. 2024). Studies however showed that, for individuals with MSA with do-not-resuscitate orders, discussions on advance healthcare directives often occurred only few hours before death (Dayal et al. 2017), highlighting major unmet needs for tailored healthcare strategies throughout the disease course.

We hypothesize that, while efforts are underway worldwide to develop effective disease-modifying strategies, establishing protocols for multidisciplinary, patient-centered symptomatic care may influence the course of MSA by improving the QoL of affected individuals (Bloem et al. 2020) and potentially reducing the occurrence of life-threatening medical complications. Various studies across Europe and the US are exploring different support approaches, including tailored counselling (NCT04965922), educational programs (NCT05819957), assessments of multidisciplinary care impact (NCT03811808), healthcare service utilization (NCT06645626), and remote integrated care to improve monitoring and reduce hospital visits (NCT05792332). MeDeMSA Care is the first study integrating in-person multidisciplinary visits with mobile palliative care, medical, psychological, and neurorehabilitation counselling via telemedicine for individuals with MSA. In this context, mobile palliative care teams may help to overcome the physical barriers between people with MSA and their specialists, particularly towards the end of life. Even though telemedicine in MSA remained underexplored so far, digital health technologies likewise offer potential for cost-effective, high-quality care delivered throughout the disease course (Pinto et al. 2017). Additional measures are likely required for a safe telerehabilitation practice. For this purpose, the MeDeMSA Care neurorehabilitation interventions have been designed in a modular format with varying levels of difficulty (see Supplementary Materials 2, 3 and 4), enabling personalized recommendations based on the level of disability and neurorehabilitation needs identified during in-person assessments. Satisfaction surveys will ultimately help to identify barriers and strategies to improve this novel treatment format. Psychological counselling over telemedicine can additionally provide support to individuals living with MSA and their caregivers in coping with fears and concerns. Despite the high symptomatic burden of MSA, caregiver burden remains in fact poorly understood and should be factored into health-economic studies (Chan et al. 2023). We hypothesize that a multidisciplinary approach, including mobile palliative care and telemedicine, could improve disease management and consequently reduce caregiver burden, particularly in advanced stages.

In clinical practice, access to multidisciplinary care is often limited by the scarcity of specialized services outside MSA referral centers (Coon et al. 2022; Reis-Carneiro et al. 2024) and the uneven distribution of care providers across countries (Habek et al. 2022). Centralized care models contribute to such disparity, as individuals residing farther from MSA referral centers often receive suboptimal care and remain underrepresented in clinical studies (Campese et al. 2024). Beyond the trial horizon and the abovementioned strategies to abate physical barriers, the MeDeMSA Care study aims to bridge this gap in healthcare access by developing test-treatment algorithms, which include widely available diagnostic tools and easily accessible, scalable, therapeutic options, including neuro-rehabilitation. Such algorithms are made available to the readership in the supplementary material of the present manuscript (see Supplementary Materials 1, 2, 3, 4) and may assist neurologists outside referral centers in delivering multidisciplinary MSA care.

## Potential study limitations

MeDeMSA Care's monocentric design and MSA’s rapid progression pose recruitment and retention challenges, with risks of dropout due to treatment noncompliance or fatal events. Recruitment empowering strategies are therefore regularly implemented. The re-assessment of the patient’s health-care goals at month-12 should also minimize the risk of incompliance. In case the drop-out rate will exceed current estimations, recruitment may be extended to achieve the targeted population size. To ensure continuity of care, individuals not randomized to the telemedicine arm may replace in-person follow-ups with telemedicine visits if disease progression prevents travel. While this safeguards ongoing care to the individuals participating in the MeDeMSA Care study, it may introduce a retention bias, favoring more severe cases that would have likely been lost to follow-up in the EMSA-SG study.

## Future directions

Upon proved feasibility and positive impact on QoL, along with health-economic evaluations, we aim to translate the MeDeMSA Care findings into actionable recommendations for national and international policymakers, guideline developers, and public health authorities to allocate the necessary resources to raise the standards of MSA care within and outside of referral centers.

Given MSA's progressive nature and its impact on patients and caregivers, integrating multidisciplinary care models into healthcare systems is essential for improving symptom management, prevent complications, and provide long-term support. In Tyrol, integrated care frameworks for stroke (PMID: 32,954,239; https://www.i-med.ac.at/neurologie/patienten/ambulanzen/stroke_card.html), heart failure (PMID: 34,269,863; https://www.herzmobil-tirol.at/page.cfm?vpath=index), and amyotrophic lateral sclerosis (https://www.hospiz-tirol.at/begleitung/als-netzwerk) successfully combine specialist expertise with community-based support, ensuring continuity of care across all disease stages. Implementing a similar model for MSA may help to bridge the gap between early-stage symptom management and late-stage palliative care.

Ultimately, we aim to shift MSA care from a fragmented, crisis-driven model to a proactive, patient-centered approach that ensures specialized support throughout the disease course.

## Supplementary Information

Below is the link to the electronic supplementary material.

Supplementary file1 (PDF 1833 KB)

Supplementary file2 (PDF 4748 KB)

Supplementary file3 (PDF 2986 KB)

Supplementary file4 (PDF 666 KB)

## Data Availability

We confirm that all the study data are contained in the main manuscript and its supplementary materials.
