# Supplementary material for: MeDeMSA care study protocol: developing personalized best medical care with integrated mobile palliative and telemedicine support for individuals with multiple system atrophy
Source: J Neural Transm (Vienna). 2025 May 24;133(5):903–17. doi: 10.1007/s00702-025-02933-z (PMC13216168; doi:10.1007/s00702-025-02933-z)
Supplement: Supplementary file 1 — Supplementary file1 (PDF 1833 KB) [file 702_2025_2933_MOESM1_ESM.pdf]

Parkinsonism

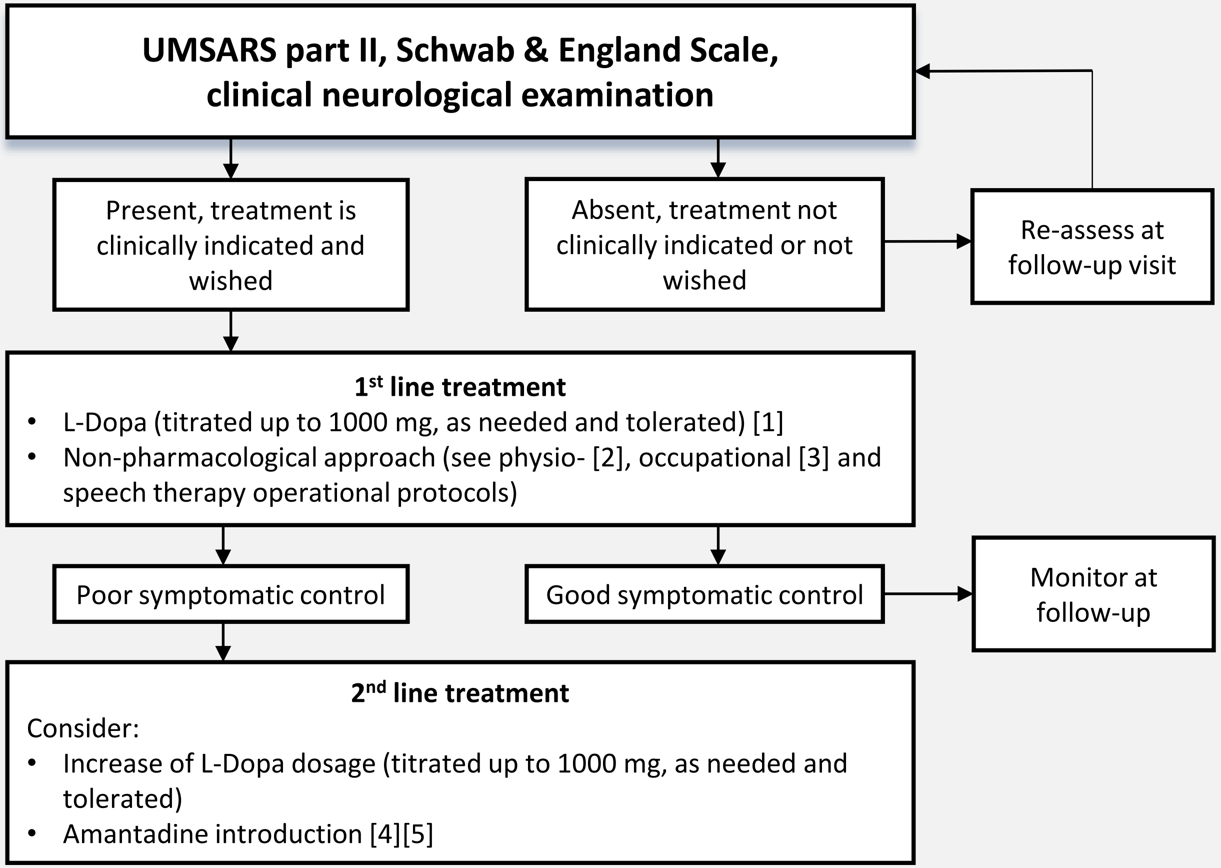

UMSARS: Unified Multiple System Atrophy Rating Assessment

[1] https://movementdisorders.onlinelibrary.wiley.com/doi/epdf/10.1002/mds.27701
[2] https://pubmed.ncbi.nlm.nih.gov/31621609/
[3] https://movementdisorders.onlinelibrary.wiley.com/doi/10.1002/mds.20211
[4] https://journals.lww.com/clinicalneuropharm/Abstract/2005/09000/Placebo_Controlled_Trial_of_Amantadine_in.4.aspx
[5] https://www.sciencedirect.com/science/article/abs/pii/S1353802097000229?via%3Dihub

Cerebellar ataxia

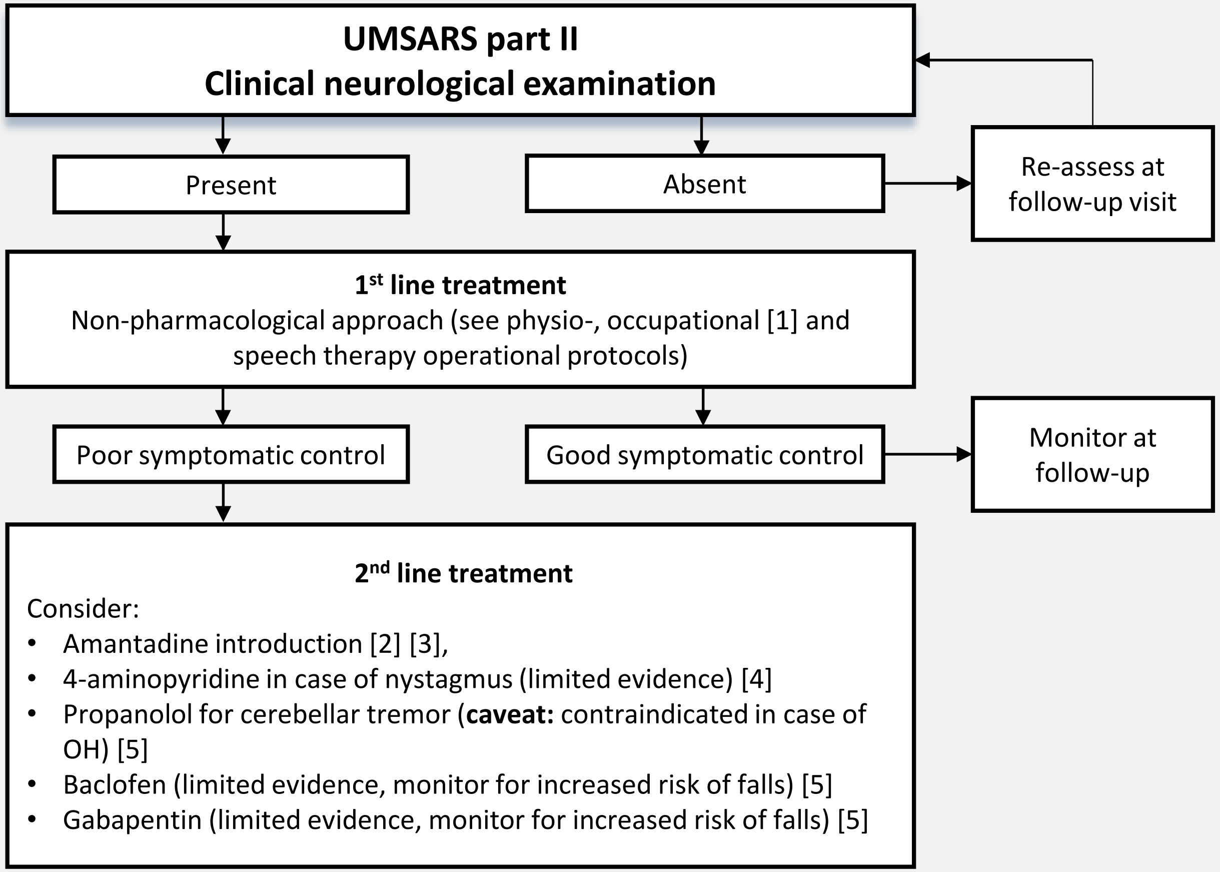

UMSARS: Unified Multiple System Atrophy Rating Assessment; OH: Orthostatic Hypotension.

[1] https://movementdisorders.onlinelibrary.wiley.com/doi/10.1002/mds.20211
[2] https://journals.lww.com/clinicalneuropharm/Abstract/2005/09000/Placebo_Controlled_Trial_of_Amantadine_in.4.aspx
[3] https://www.sciencedirect.com/science/article/abs/pii/S1353802097000229?via%3Dihub
[4] https://www.ncbi.nlm.nih.gov/pmc/articles/PMC4344126/
[5] https://www.ncbi.nlm.nih.gov/pmc/articles/PMC3002658/#bibr160-1756285610375328

Dystonia

Inquire about cramps
Ask about L-Dopa-induced dystonia
Clinical neurological examination

Present

Absent → Re-assess at follow-up visit

1st line treatment
- BTX for focal dystonia (e.g. hands) [1] [2]
- Non-pharmacological approach (see physio-, occupational [3] and speech therapy operational protocols)
- Consider adjusting L-Dopa dosage in case of L-Dopa-induced dystonia [4]
- Add-on amantadin

Poor symptomatic control

Good symptomatic control → Monitor at follow-up

2nd line treatment
Consider:
- Central anticholinergics (e.g., biperidene; monitor for cognitive side-effects) [1]
- Clonazepam [1] (if myoclonus is present, monitor for increased risk of falls)

BTX: Botulinum toxin.

[1] https://www.sciencedirect.com/science/article/pii/S1474442206705749?via%3Dihub
[2] https://www.ncbi.nlm.nih.gov/pmc/articles/PMC7969540/
[3] https://movementdisorders.onlinelibrary.wiley.com/doi/10.1002/mds.20211
[4] https://jnnp.bmj.com/content/72/3/300

Spasticity

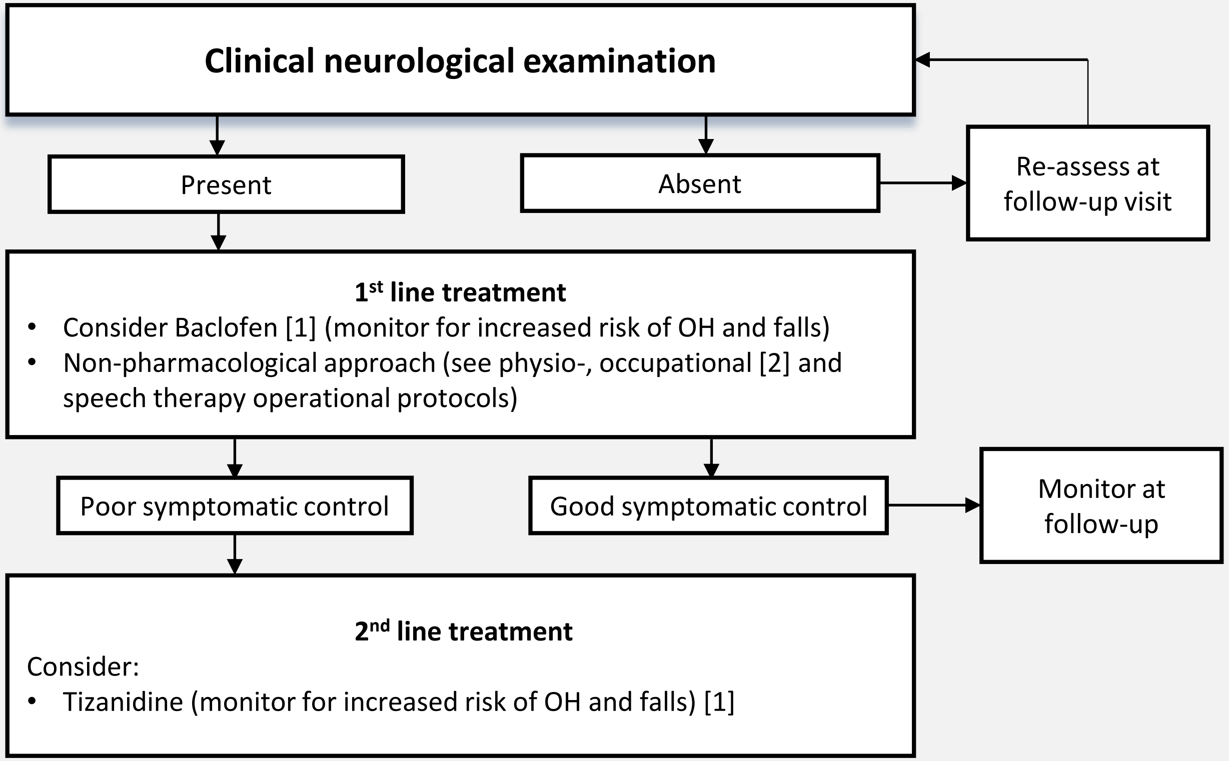

OH: Orthostatic Hypotension.

[1] https://pn.bmj.com/content/12/5/289.long

[2] https://movementdisorders.onlinelibrary.wiley.com/doi/10.1002/mds.20211

Recurrent falls

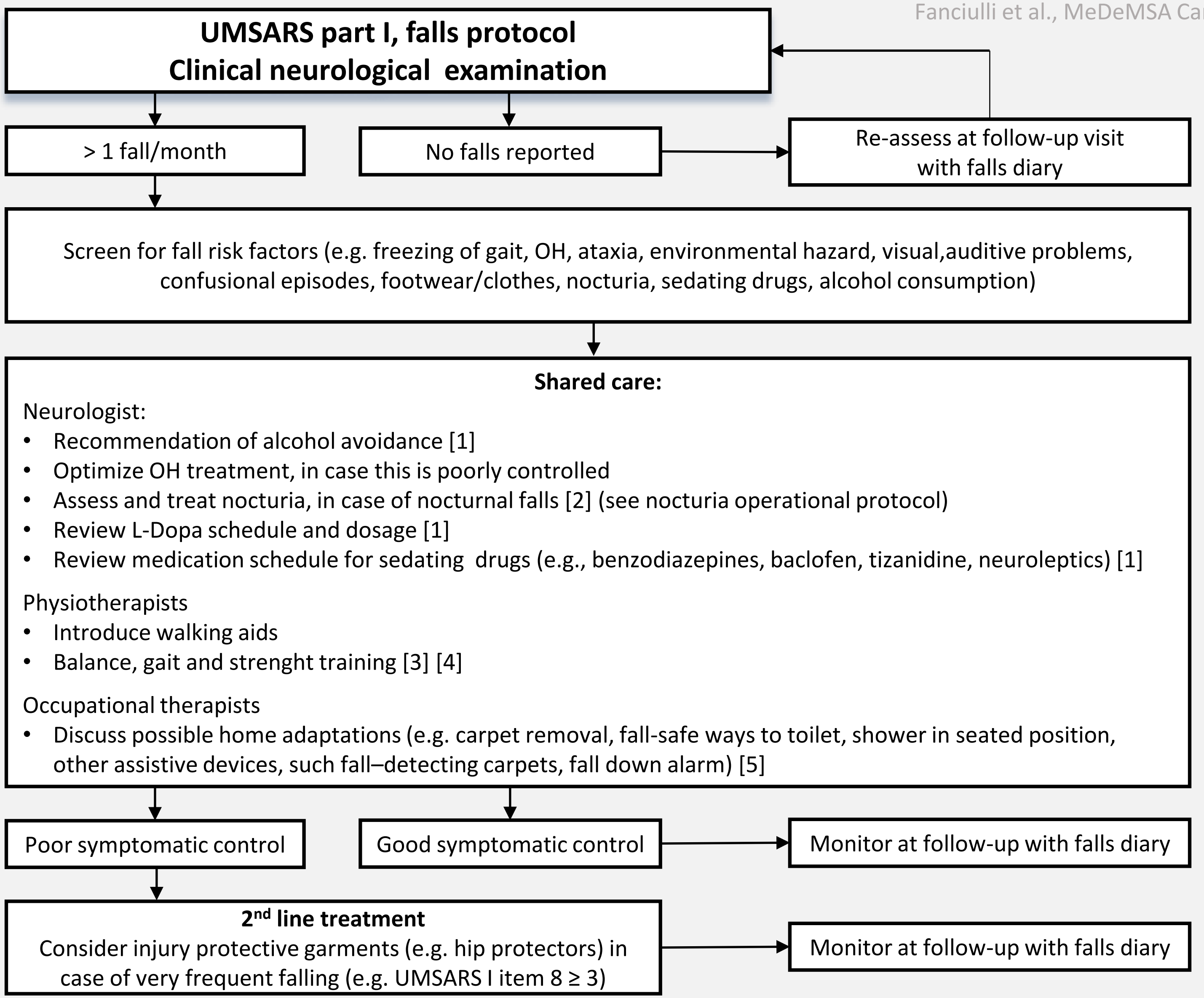

OH: Orthostatic Hypotension.
UMSARS: Unified Multiple System Atrophy Rating Assessment.

[1] https://www.sciencedirect.com/science/article/pii/S1353802013004471?via%3Dihub
[2] https://www.cambridge.org/core/journals/canadian-journal-of-neurological-sciences/article/falls-in-synucleinopathies/1C3F91AB55AB37D8AB61CFF9E2B6E4E4
[3] https://journals.sagepub.com/doi/10.1177/1545968314565511?url_ver=Z39.88-2003&rfr_id=ori:rid:crossref.org&rfr_dat=cr_pub%20%200pubmed
[4] https://pubmed.ncbi.nlm.nih.gov/31621609/
[5] https://journals.sagepub.com/doi/10.1177/0898264308324672?url_ver=Z39.88-2003&rfr_id=ori:rid:crossref.org&rfr_dat=cr_pub%20%200pubmed

Gait impairment: physiotherapeutic approach

FAC

- **< 3 points**
  - Using assistive devices
  - Transfer training: from sit to stand, from bed/chair to chair, …
  - Mobility training: standing unsupported, stepping exercises, weight shifting, trunk movements.
- **≥ 3 points**
  - **TUG [1]**
    - **< 20 sec**: **High level gait training with focus on changing positions**
      - Gait training without assistive devices
      - Walking aids
      - Longer distances, more variations, dual tasking (such as enumeration, calculation)
      - Walking around and over obstacles
      - Gait training on different surfaces
    - **≥ 20 sec**: **Low level gait training with focus on changing positions** (with support of therapists/ caregivers, walking aids):
      - Stepping training
      - Walking reduced distances
      - Increased number of breaks
      - Less variations
      - Training transfers
      - Cueing training against freezing of gait
  - **10 MWT [2]**
    - **< 12,5 sec**: **High level gait training with focus on pace**
      - Gait training without assistive devices, walking aids
      - Longer distances, changing pace and directions, more variations
      - Dual tasking (such as enumeration, calculation)
      - Walking around and over obstacles
      - Gait training on different surfaces
    - **≥ 12,5 sec**: **Low level gait training with focus on pace** (with support of therapists/ caregivers, walking aids, furniture,…):

      See high level, but:
      - Reduced distances
      - Increased number of breaks
      - Less variations

FAC: Functional Ambulation Categories; TUG: Time Up-and-Go; 10 MWT: 10 meters walking test.

[1] https://medicaljournalssweden.se/jrm/article/view/10427
[2] https://journals.lww.com/jgpt/Fulltext/2016/10000/Test_Retest_Reliability_and_Minimal_Detectable.3.aspx

Cerebellar ataxia: physiotherapic approach

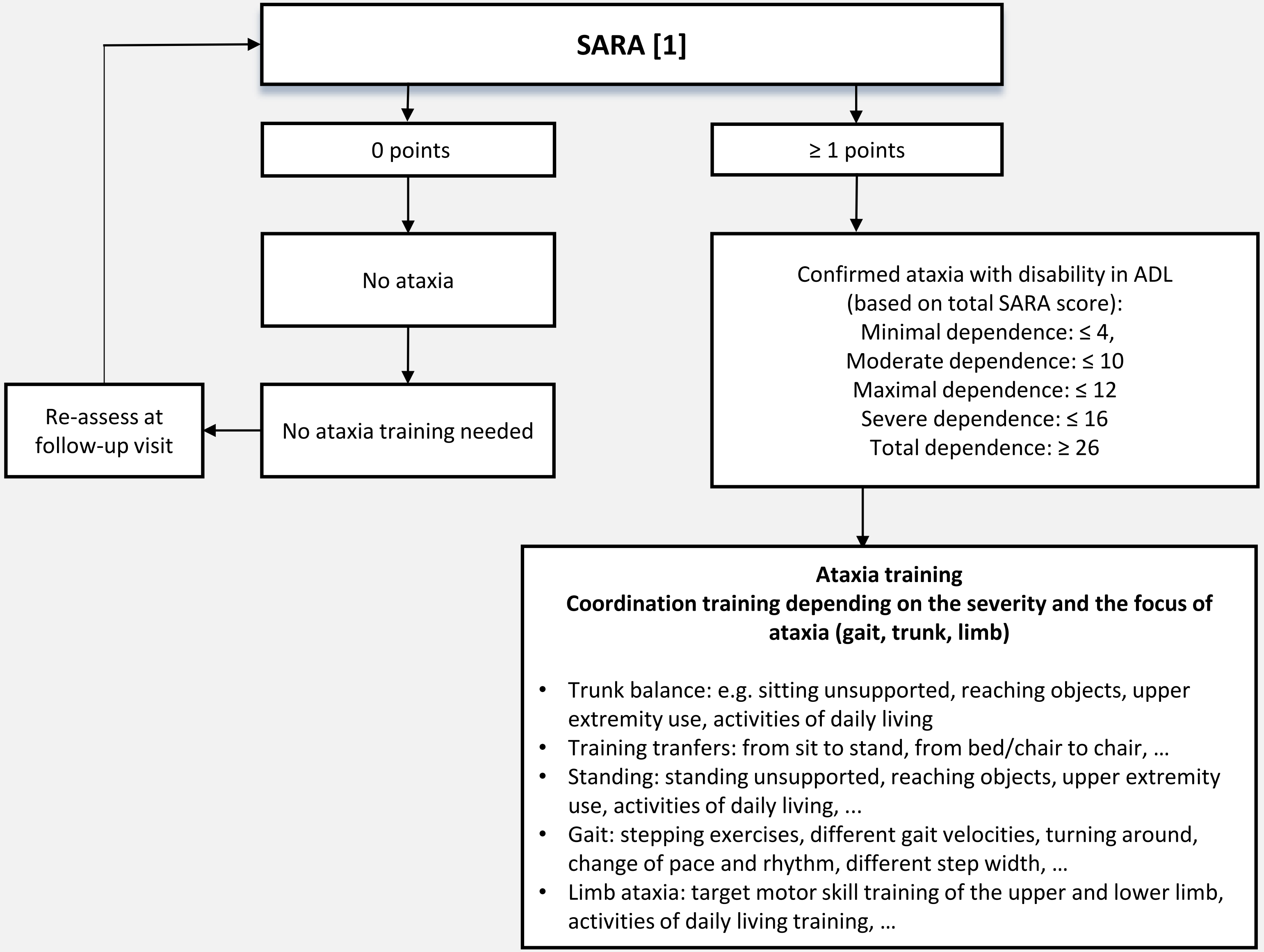

SARA: Scale of the Assessment and Rating of Ataxia.

[1] https://pubmed.ncbi.nlm.nih.gov/16769946/

Balance impairment: physiotherapic approach

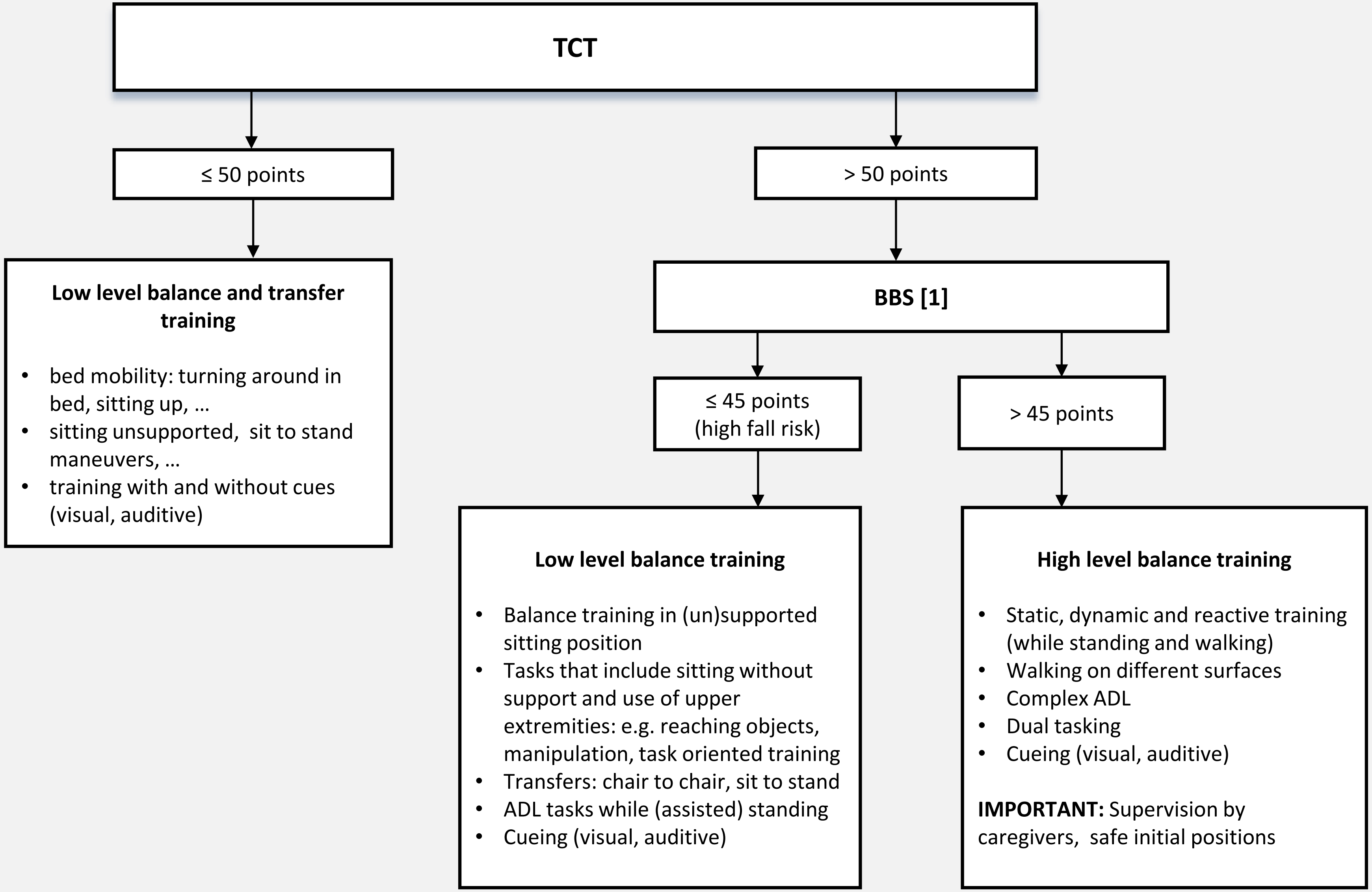

TCT: Trunk Control Test; BBS: Berg Balance Scale; ADL: activities of daily living.

[1] https://ijclinmedcasereports.com/pdf/IJCMCR-RW-00176.pdf

Activities of daily living: occupational therapy approach

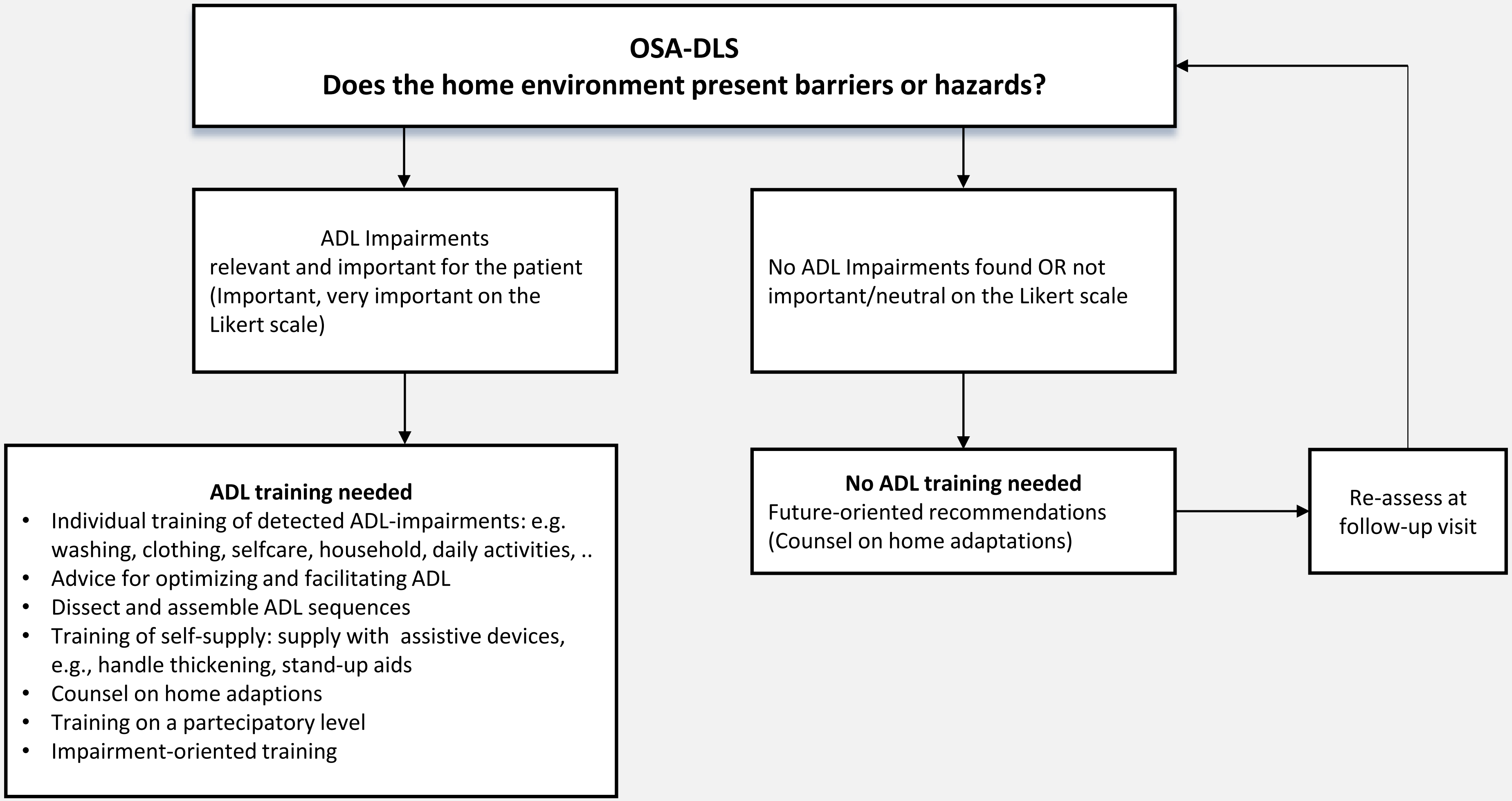

OSA-DLS: Occupational Self Assessment- Daily Living Scale.
https://research.aota.org/ajot/article-abstract
Psychometric testing of the Icelandic Occupational Self-Assessment (OSA-IS

Motor Skills: occupational therapy approach

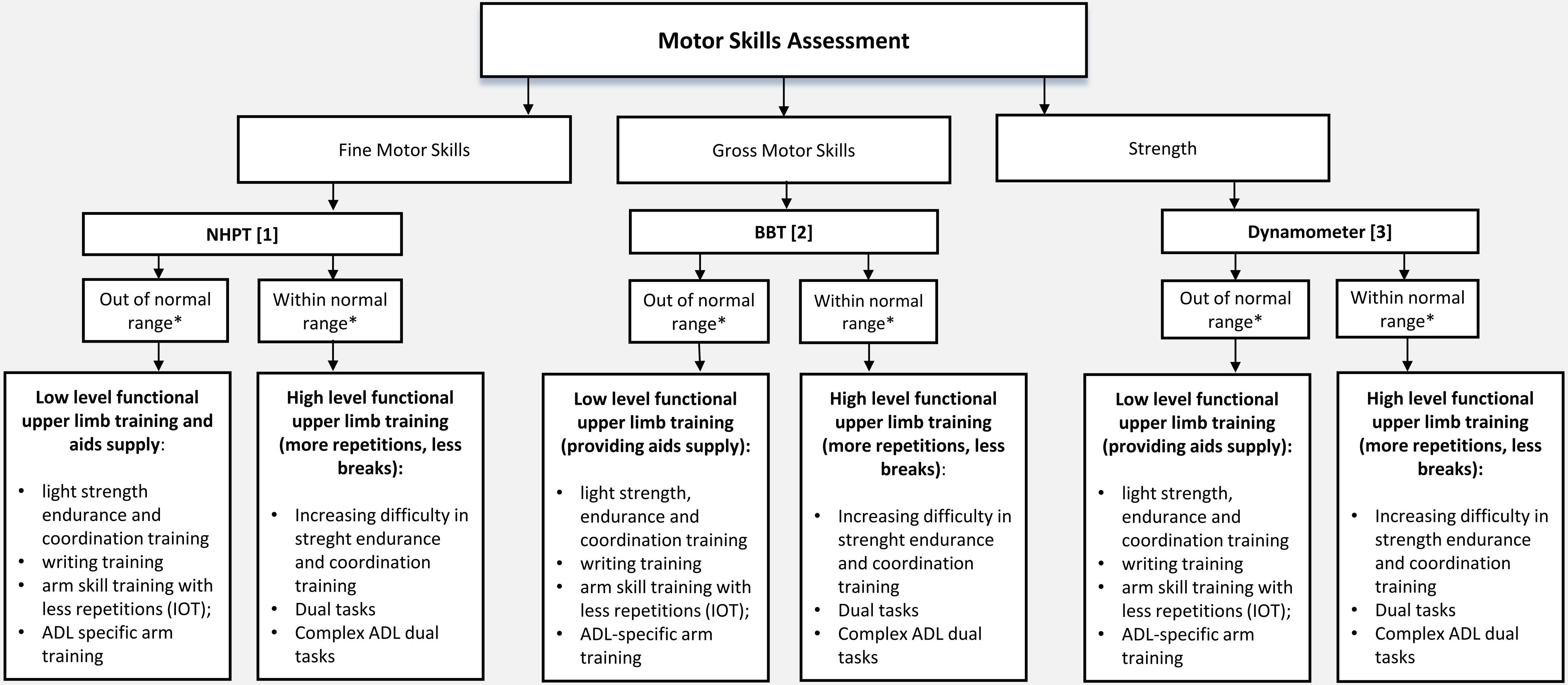

BBT: Box and Block Test; NHPT: Nine Hole Peg Test; IOT: impairment-oriented training.

*: According to the standard value lists broken down by age, gender, handedness and left or right side – see detailed description in Schädler, S et al. (2020): Assessments in der Rehabilitation. Band 1 Neurologie (4. Aufl.). Bern: Huber.

[1] Exploring the ability of strength and dexterity tests to detect hand function impairment in individuals with Parkinson's disease - PubMed (nih.gov).
[2] Assessment of Manual Dexterity in VR: Towards a Fully Automated Version of the Box and Blocks Test - PubMed (nih.gov)
[3] Grip Work Measurement with the Jamar Dynamometer: Validation of a Simple Equation for Clinical Use - PubMed (nih.gov)

Urinary disturbances 1/2

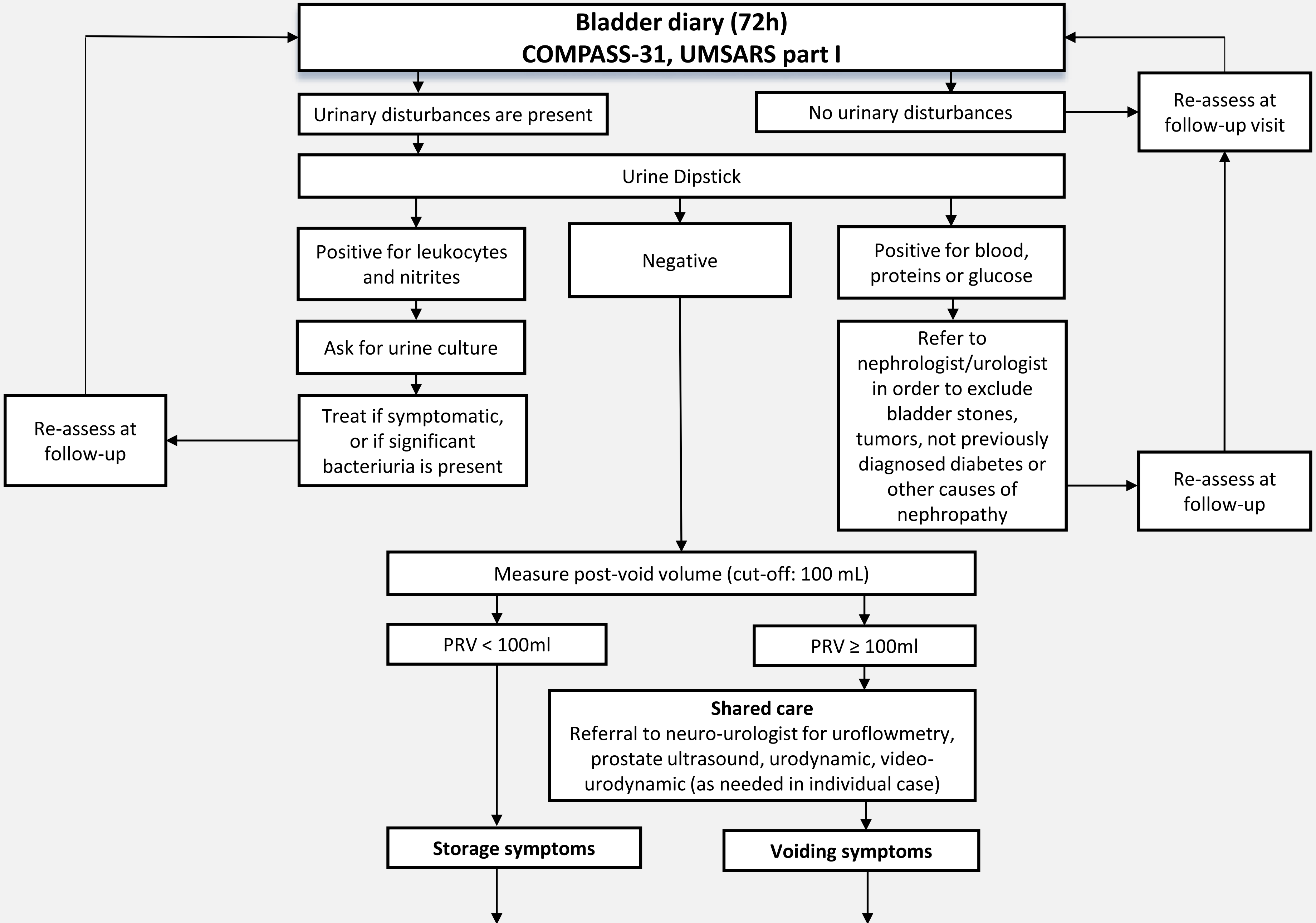

Urinary disturbances 2/2

Storage symptoms

1st line treatment [1]

Non-pharmacological
- Limit caffeine intake
- Consider removal or dose reduction of aggravating drugs (e.g.: α-adrenoceptors antagonist to treat benign prostatic hypertrophy or arterial hypertension)
- Prescribe absorbent pads, external collection devices

Pharmacological:
- Antimuscarinics: e.g., trospium (monitor post-void residual volume at follow-up)
- Mirabegron (monitor for increased risk of aggravating/exacerbating supine hypertension)

Good symptomatic control → Monitor at follow-up

Poor symptomatic control ↓

2nd line treatment [1]

Shared care with neuro-urologist:
Consider combining anticholinergics with mirabregon

Good symptomatic control → Monitor at follow-up

Poor symptomatic control ↓

3rd line treatment [1]

Consider: bladder detrusor BTX injection

Voiding symptoms

1st line treatment

Consider removal or dose reduction of aggravating drugs
(e.g. urinary retention due to anticholinergics, mirabegron or sympathomimetic drugs for treating OH)

Good symptomatic control → Monitor at follow-up

Poor symptomatic control ↓

2nd line treatment [1]

Clean intermittent self-catheterization
1x cath./100 mL retained urine/day, e.g.,
3x cath./day, if post-void urine volume = 300 mL

Important: consider as 1st line treatment, if there are no aggravating drugs scheduled or these cannot be reduced.

Good symptomatic control → Monitor at follow-up

Poor symptomatic control ↓

3rd line treatment

In case voiding symptoms cannot be managed (anymore) with any of the above-mentioned strategies, counsel about:
- suprapubic catheterization (preferred choice)
- transurethral indwelling catheterization

BTX: botulinum toxin; COMPASS-31: Composite Autonomic Symptom Score; UMSARS: Unified Multiple System Atrophy Rating Assessment; PVR: post-void residuum.

[1] https://d56bochluxqnz.cloudfront.net/documents/full-guideline/EAU-Guidelines-on-Neuro-Urology-2023.pdf

Nocturia

How many toilet visits per night on average?
Bladder diary for up to 72h

One or more toilet visits/night [1] and treatment is wished

Absent

Re-assess at follow-up visit

1st line treatment [2]

- Reduce fluid intake in the second half of the day
- Avoid caffeine and alcohol intake in the evening
- Ensure a fall-safe way to the toilet
- Consider: bedside commode, flask, absorbent pads, external collection devices

>= 2 toilet visits/night

Good symptomatic control

Monitor at follow-up

2nd line treatment [3]

Exclude nocturnal hypertension

Shared care with neuro-urologist:

- Trospium at night
- Desmopressin spray at night- in case of nocturnal polyuria (monitor sodium levels and weight at follow-up)

Reconsider: bedside commode, flask, absorbent pads, external collection devices

[1] https://onlinelibrary.wiley.com/doi/10.1002/nau.10053
[2] https://www.sciencedirect.com/science/article/pii/S0302283818300022?via%3Dihub
[3] https://d56bochluxqnz.cloudfront.net/documents/full-guideline/EAU-Guidelines-on-Neuro-Urology-2023.pdf

Male erectile dysfunction

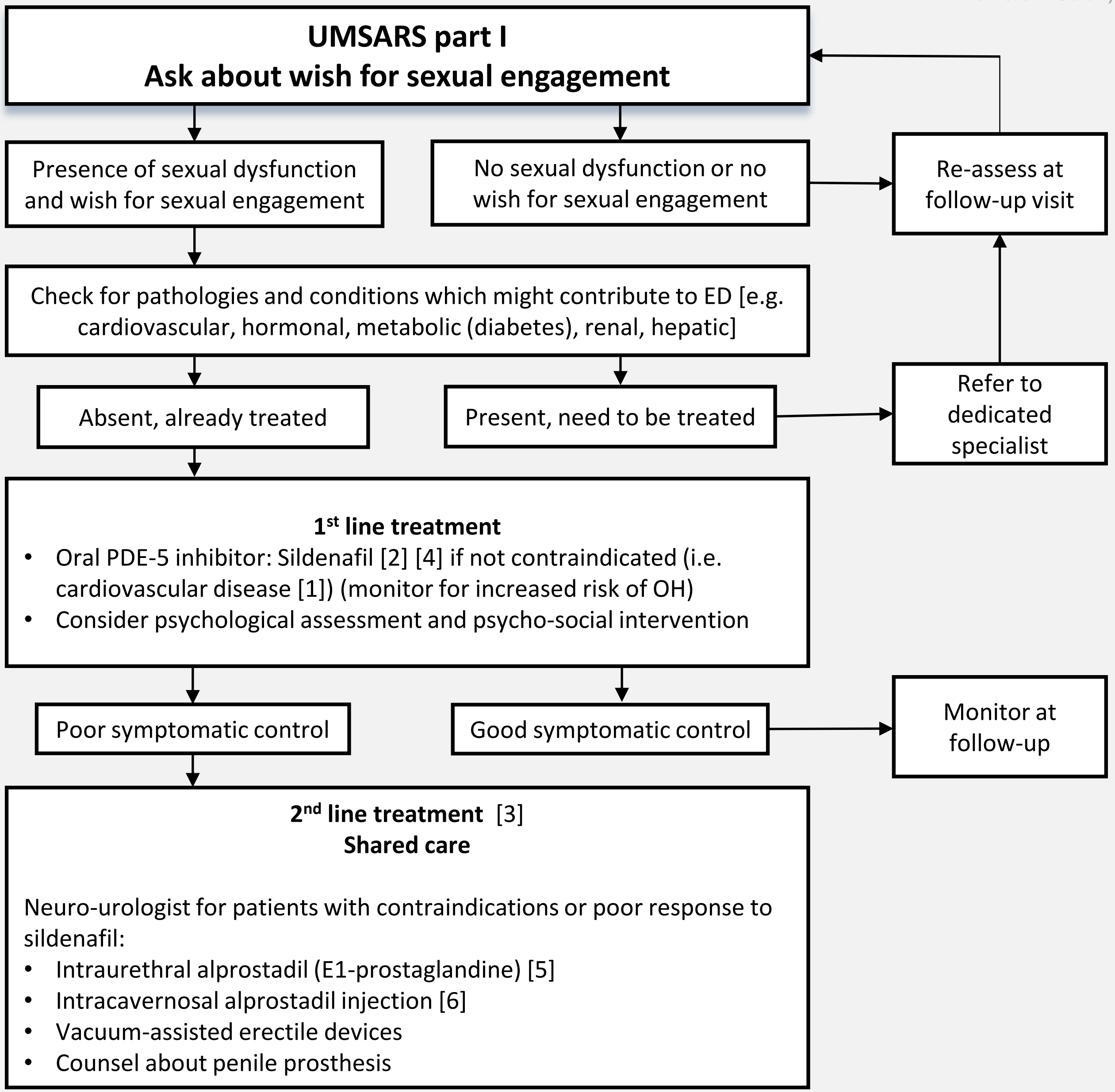

ED: Erectile Dysfunction.
OH: Orthostatic Hypotension.
UMSARS: Unified Multiple System Atrophy Rating Assessment.

[1] https://www.ncbi.nlm.nih.gov/pmc/articles/PMC3498391/
[2] https://www.ncbi.nlm.nih.gov/pmc/articles/PMC1737541/
[3] https://www.auajournals.org/doi/10.1016/j.juro.2018.05.004
[4] https://www.ncbi.nlm.nih.gov/pmc/articles/PMC1737541/
[5] https://bjui-journals.onlinelibrary.wiley.com/doi/full/10.1046/j.1464-410x.2000.00723.x
[6] https://www.nejm.org/doi/10.1056/NEJM199604043341401?url_ver=Z39.88-2003&rfr_id=ori:rid:crossref.org&rfr_dat=cr_pub%20%200www.ncbi.nlm.nih.gov

Female sexual dysfunction

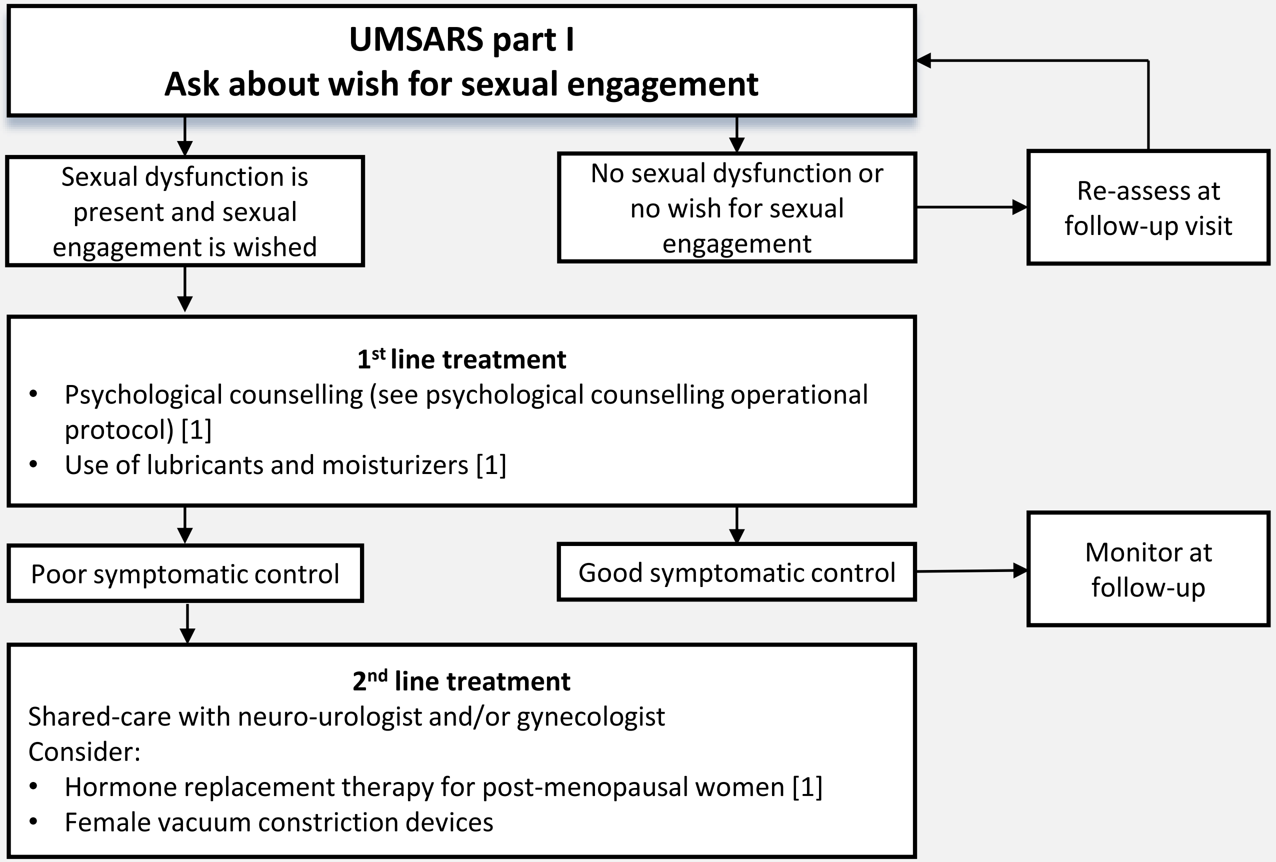

UMSARS: Unified Multiple System Atrophy Rating Assessment.

[1] https://journals.lww.com/greenjournal/Fulltext/2019/07000/Female_Sexual_Dysfunction__ACOG_Practice_Bulletin.45.aspx

Orthostatic hypotension 1/2

Home supine to standing BP diary for up to 72h
COMPASS-31
Inquire about post-prandial or exercise-induced lightheadedness/dizziness
Standing test [2]

→ Positive
→ Negative → Re-assess at follow-up visit (→ back to top)

Positive →

Consider:
- Cardiovascular autonomic function tests (in case of OH symptoms, but negative standing test/home BP diary)
- 24h-ABPM (in case the BP diary indicates moderate to severe supine hypertension or positive history for post-prandial/exercise-induced hypotension)

→ OH confirmed*
→ OH not confirmed → Rule out/treat confounders (e.g. dehydratation, drugs) → Re-assess at follow-up visit

OH confirmed* →

1st line treatment
- Removal or dose reduction of aggravating drugs (e.g. blood pressure lowering drugs, CNS-acting drugs with BP lowering effect, etc) [3]
- Lifestyle measures [4]: stand up slowly, avoid heat exposure; in case of dizziness sit or lay down; if not possible perform BP raising manouvres; sleep in 10-20° head-up tilted position
- Increased salt intake (≥ 8 g or 150 mmol /day) and water (2–2.5 l/day), if not contraindicated) [4]
- Recommend elastic stockings and/or abdominal binders (if there´s no suprapubic catheterization) [4]
- Schedule the main meal in the evening

For post-prandial hypotension: fragmented meals with low carbohydrate content; alcohol avoidance; caffeine before meals [4] [5]

→ Poor symptomatic control →
→ Good symptomatic control → Monitor at follow-up

***Orthostatic Hypotension:** a sustained reduction of systolic blood pressure of at least 20 mmHg and/or diastolic blood pressure of 10 mmHg within 3 min of standing or head-up tilt to at least 60° on a tilt table [1]

Orthostatic hypotension 2/2

Poor symptomatic control despite non-pharmacological measures or pharmacological treatment clinically indicated (e.g. very frequent syncope) and wished

2nd line treatment

For OH:

- Etylephrine
- Midodrine [6]
- Fludrocortisone [7]

Caveat: monitor for supine hypertension at follow-up

For post-prandial hypotension: consider acarbose before meals [8]

Poor symptomatic control

Good symptomatic control → Monitor at follow-up

3rd line treatment

- Combine more pressor agents [9]

Consider:

- Droxidopa [6]
- Pyridostigmine [6]

COMPASS-31: Composite Autonomic Symptom Score; 24h- ABPM- 24 hours Ambulatory Blood Pressure Monitoring.

[1] https://www.sciencedirect.com/science/article/pii/S156607021100035X?via%3Dihub
[2] https://link.springer.com/article/10.1007/s10286-019-00619-7
[3] https://pubmed.ncbi.nlm.nih.gov/32894454/
[4] https://www.ncbi.nlm.nih.gov/pmc/articles/PMC7592655/
[5] https://www.nejm.org/doi/10.1056/NEJM198508293130905?url_ver=Z39.88-2003&rfr_id=ori:rid:crossref.org&rfr_dat=cr_pub%20%200pubmed
[6] https://pubmed.ncbi.nlm.nih.gov/28050656/
[7] https://www.ncbi.nlm.nih.gov/pmc/articles/PMC5686257/
[8] https://www.ahajournals.org/doi/10.1161/HYPERTENSIONAHA.107.091355?url_ver=Z39.88-2003&rfr_id=ori:rid:crossref.org&rfr_dat=cr_pub%20%200pubmed
[9] https://n.neurology.org/content/38/6/951

Supine and nocturnal hypertension

Ask about frequent nocturia episodes at history taking
Home supine to standing BP diary for up to 72h
Consider 24h ABPM (in case the BP diary indicates moderate to severe supine hypertension) [1]

Present

Absent → Re-assess at follow-up visit

Mild supine hypertension
(systolic BP 140–159 mmHg or diastolic BP 90–99 mmHg)
Non-pharmacological measures

- Avoid the supine position during daytime, when pressor agents are scheduled [2]
- Reduce fluid intake before bedtime
- Sleep in 10-20° head-up tilted position
- Encourage snack with high carbohydrate content before bedtime
- Screen for sleep disordered breathing and nocturnal pain (see dedicated operational protocol)

Moderate supine hypertension
(systolic BP 160–179 mmHg or diastolic BP 100–109 mmHg)

In addition to the therapy for "mild" SH :
- Avoid pressor agents in the afternoon [3]
- Avoid long-acting pressor agents; if unavoidable for OH symptomatic control, consider bedtime blood pressure lowering drugs

Severe supine hypertension
(systolic BP ≥ 180 mmHg or diastolic BP ≥ 110 mmHg) **overnight**

In addition to the therapy for "mild" and "moderate" SH, consider short-acting antihypertensive drugs at bedtime:

- Nitroglycerine patch (overnight only)
- Losartan
- Sildenafil
- Clonidine (schedule early in the evening)

Recommend to stand-up cautiously at night because of OH risk

BP: blood pressure; OH: orthostatic hypotension; ABPM: ambulatory blood pressure monitoring.

[1] https://www.ncbi.nlm.nih.gov/pmc/articles/PMC6097730/
[2] https://www.ncbi.nlm.nih.gov/pmc/articles/PMC7592655/
[3] https://journals.lww.com/jhypertension/Fulltext/2019/08000/Management_of_supine_hypertension_in_patients_with.2.aspx

Hypo/Anhidrosis

COMPASS-31
Any skin problems?

Present

Absent → Re-assess at follow-up visit

1st line treatment [1]

Preventive measures:

- Avoid strenuous activities and heat exposure
- Wear loose-fitting clothing

Behavioural measures:

- Moisturize skin
- Sufficient hydration (2-2.5 l/d if no contraindications exist)

Pharmacological measures:

- Review medication with adverse effects (i.e. anticholinergic and opioid drug dosages and schedules, if applicable and modifiable) [2]

Shared care with dermatologist/plastic surgery in case of skin complications (e.g. pressure sores, intertrigo)

Poor symptomatic control

Good symptomatic control → Monitor at follow-up

2nd line treatment
Consider use of cooling garments (e.g. bags, vests, water-sprays)

COMPASS-31: Composite Autonomic Symptom Score; Q-SART: Quantitative Sudomotor Axon Reflex Testing.

[1] https://www.thieme-connect.com/products/ejournals/abstract/10.1055/s-0040-1713844
[2] https://onlinelibrary.wiley.com/doi/10.1111/jdv.12014

Constipation 1/2

COMPASS-31

- Present
- Absent → Re-assess at follow-up visit (→ COMPASS-31)

Present → Screen at history taking for secondary causes and complications: blood in stools? Weight loss? Past history of polyps or other bowel disease? Hemorrhoids?

- Negative
- Positive → Refer to gastroenterologist/ proctologist

Negative → Review medication schedule for any anticholinergic drug and its dosage. If positive and possible, consider reducing/suspending anticholinergic drugs.

- Poor symptomatic control
- Good symptomatic control → Monitor at follow-up

Poor symptomatic control →

1st line treatment [2]

Non-pharmacological measures

- Inform about positive effects of physical exercise [1]
- Inform about positive effects of scheduling toilet visits after meals
- Increase dietary fibers (gradually to avoid adverse effects)
- Encourage sufficient water intake, i.e., at least 2-2.5L/day, if no contraindications exist
- Clockwise abdominal massage (Instructions for self-practice/with caregiver) [3] [4]

- Non-pharmacological measures already established, or pharmacological measures needed or wished →
- Good symptomatic control → Monitor at follow-up

Constipation 2/2

Non-pharmacological measures already established, or pharmacological measures needed or wished

2nd line treatment
Osmotic laxatives: macrogol, bisacodile, or sodium picosulfate

Poor symptomatic control

Good symptomatic control

Monitor at follow-up

3rd line treatment
- Combine macrogol with bisacodile or sodium picosulfate
- Prucalopride [2][5]
- Consider: domperidone

Approach for anorectal dysfunction [2]
- Enemas (warm water or glycerin) or suppositories (**caveat:** risk for anorectal injuries)
- Manual dysimpaction (**caveat:** risk for rectal injuries)

BTX: Botulinum toxin; COMPASS-31: Composite Autonomic Symptom Score.

[1] https://link.springer.com/article/10.1007/s10072-021-05041-4
[2] https://www.aafp.org/pubs/afp/issues/2015/0915/p500.html#afp20150915p500-b13
[3] https://www.cochranelibrary.com/cdsr/doi/10.1002/14651858.CD002115.pub4/full
[4] https://www.sciencedirect.com/science/article/pii/S0020748909000108?via%3Dihub
[5] https://www.ncbi.nlm.nih.gov/pmc/articles/PMC5673021/

RBD

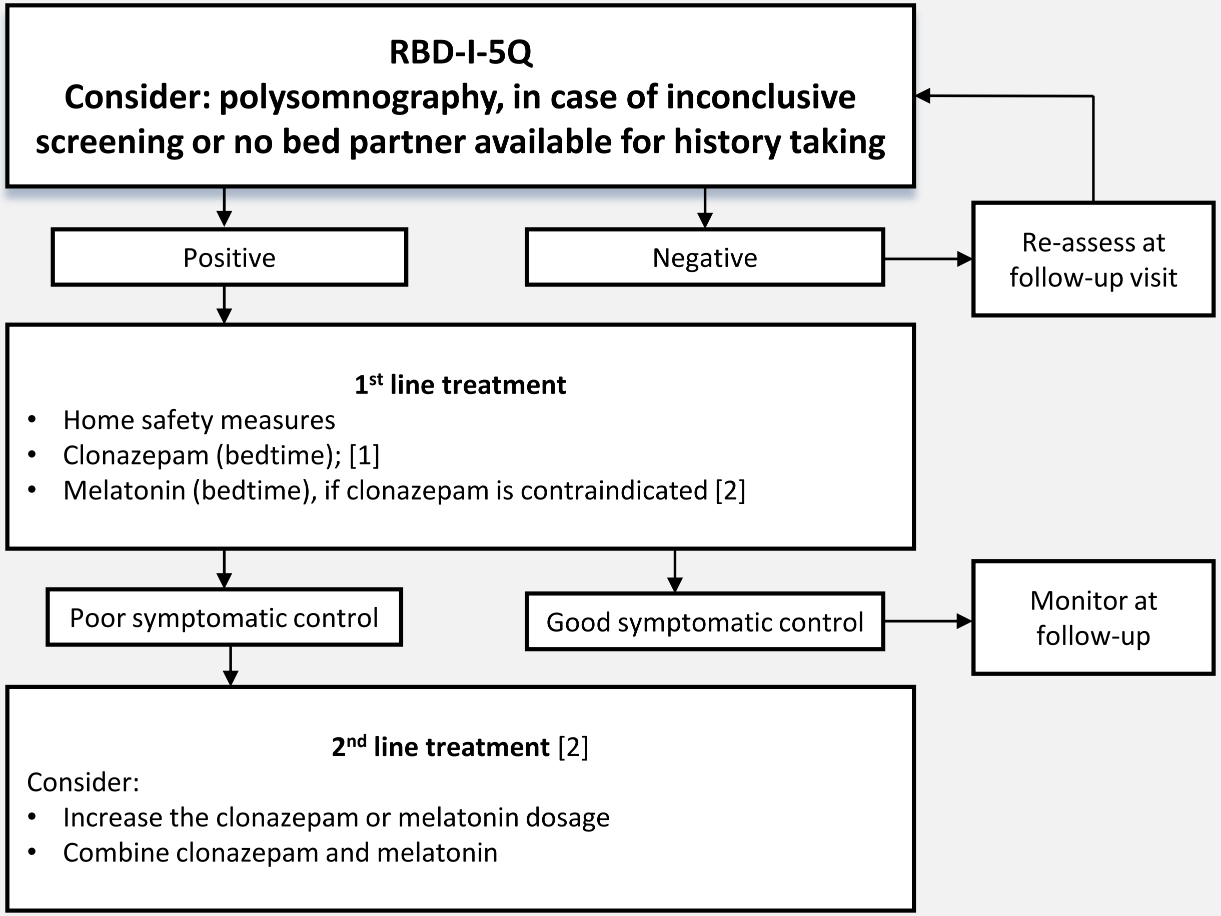

RBD-I-5Q: Innsbruck REM sleep behaviour inventory- 5 questions.

[1] https://jcsm.aasm.org/doi/10.5664/jcsm.10424
[2] https://www.ncbi.nlm.nih.gov/pmc/articles/PMC10125958/

Insomnia

ISI

Present → 1st line treatment

Absent → Re-assess at follow-up visit → ISI

1st line treatment

- Check for aggravating factors (e.g. pain, nocturia)
- Optimize treatment of other sleep disorders (e.g. RDB, OSA, RLS)
- CBT-I: [1] suggest to increase daily activity (when possible) and daytime napping restriction
- Consider psychological counselling - see psychological protocol [2] on modular interventions in case of depression or anxiety.

Poor symptomatic control → 2nd line treatment

Good symptomatic control → Re-assess at follow-up visit

2nd line treatment

Consider:

- Low dose antidepressants at bedtime: trazodone [3], mirtazapine [4][5]
- Neuroleptics in case of concomitant hallucinations, delusions [4]
- Sedative-hypnotics (low-dose clonazepam, melatonin in case of RBD) [6][7]
- Cannabidiol (at bedtime)

CBT-I: Cognitive Behavioral Therapy for Insomnia; ISI: Insomnia Severity Index.

[1] https://www.acpjournals.org/doi/full/10.7326/M15-2175?rfr_dat=cr_pub++0pubmed&url_ver=Z39.88-2003&rfr_id=ori%3Arid%3Acrossref.org
[2] https://www.ncbi.nlm.nih.gov/pmc/articles/PMC2576317/
[3] Safety and efficacy of melatonin, clonazepam, and trazodone in patients with Parkinson's disease and sleep disorders: a randomized, double-blind trial | Cochrane Library
[4] https://journals.sagepub.com/doi/10.1177/0269881116681399?url_ver=Z39.88-2003&rfr_id=ori:rid:crossref.org&rfr_dat=cr_pub%20%200pubmed
[5] https://academic.oup.com/sleep/article/43/11/zsaa092/5837058?login=true
[6] https://www.sciencedirect.com/science/article/pii/S1389945705000961?via%3Dihub
[7] https://www.ncbi.nlm.nih.gov/pmc/articles/PMC7111581/

OSA

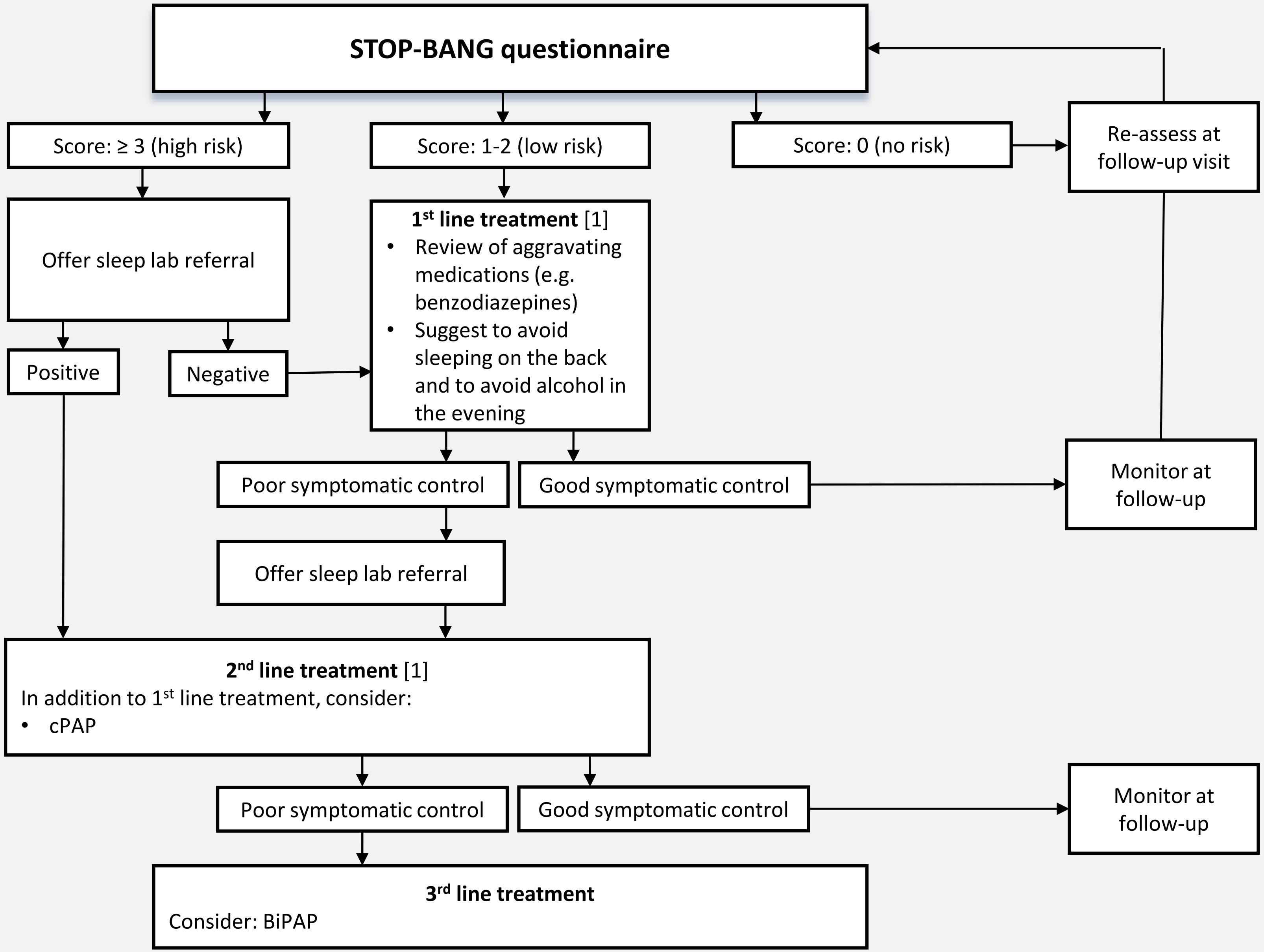

[1] https://onlinelibrary.wiley.com/doi/10.1002/alr.23079 BiPAP: Bifasic Positive Active Pressure; CPAP: continuous Positive Active Pressure; OSA: Obstructive Sleep Apnoea.

Stridor

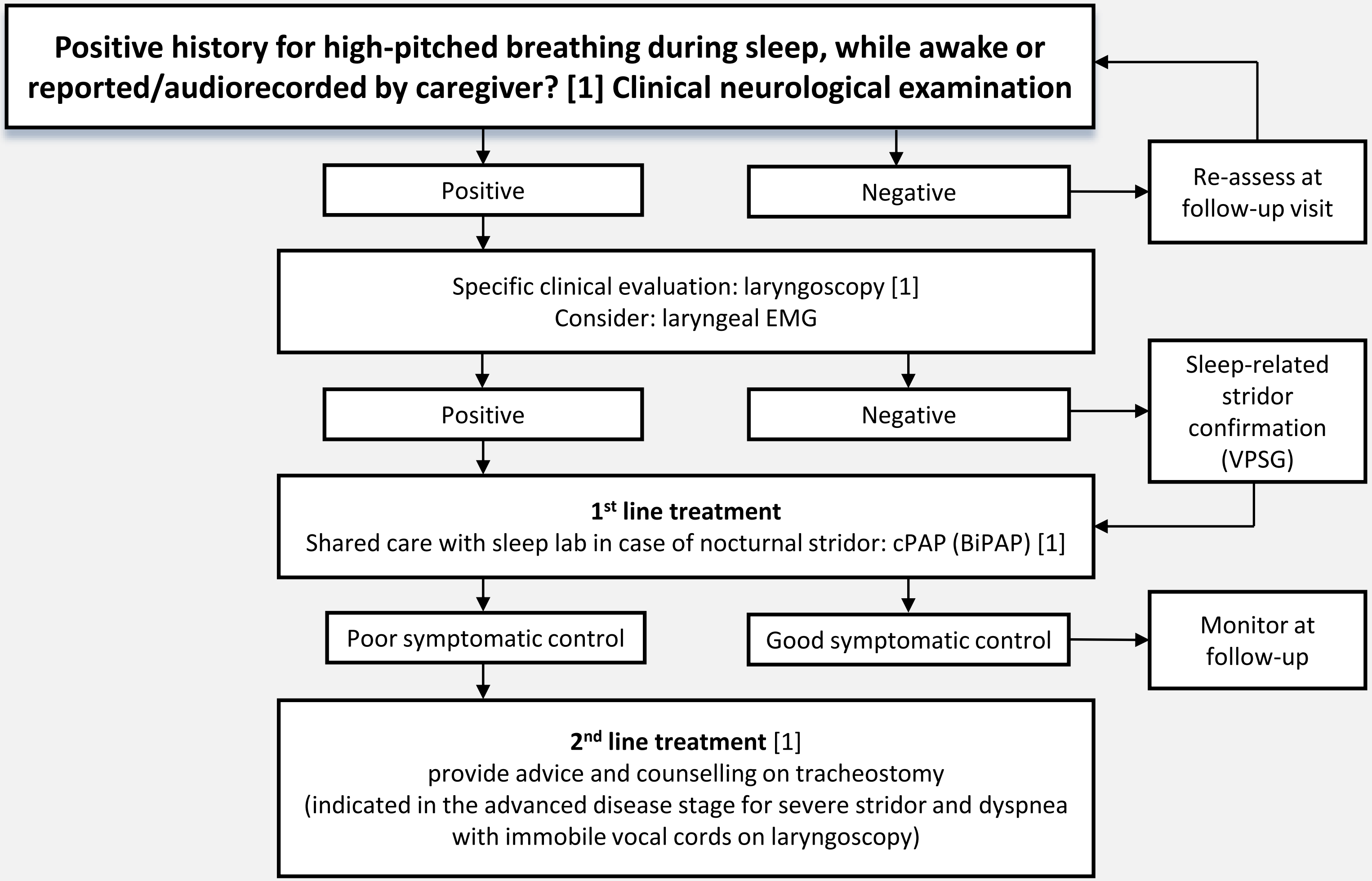

CPAP: continuous positive airway preassure; EMG: electromyography; VPSG: video polysomnography.
[1] https://n.neurology.org/content/93/14/630.long

Sialorrhea

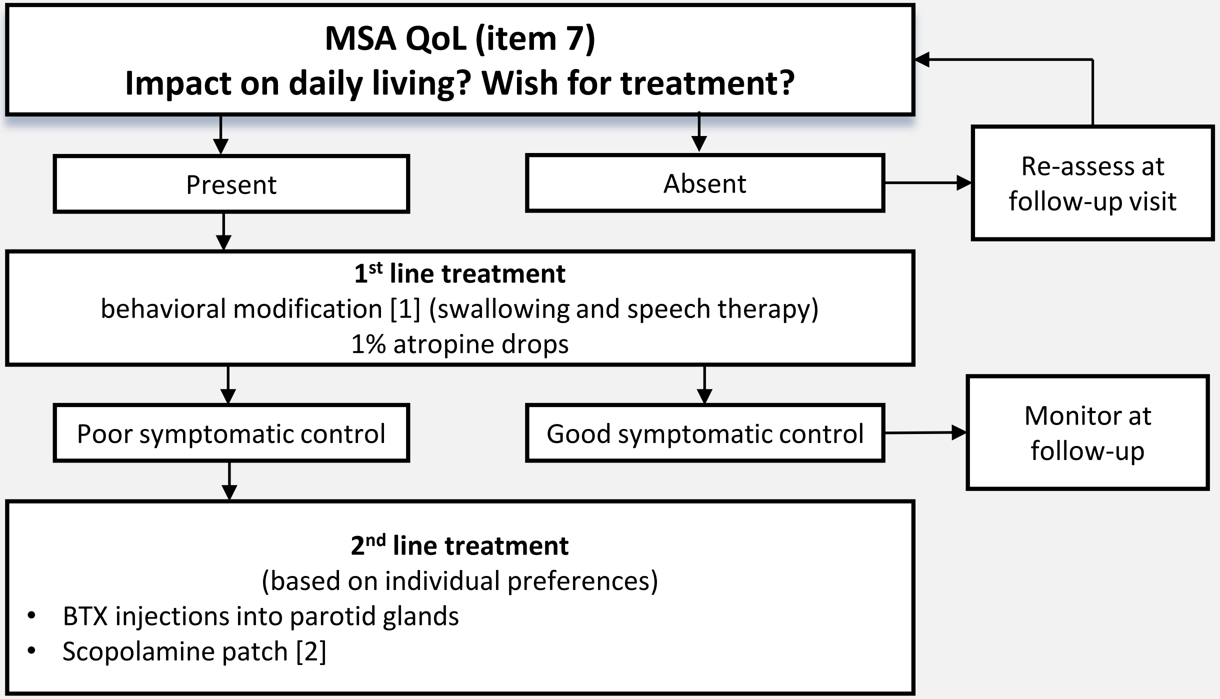

BTX: Botulinum toxin.

[1] https://journals.sagepub.com/doi/10.1177/0145445506298723?url_ver=Z39.88-2003&rfr_id=ori:rid:crossref.org&rfr_dat=cr_pub%20%200pubmed

[2] https://onlinelibrary.wiley.com/doi/10.1111/jocn.15009

Dysphagia

UMSARS I item 2>=1

MUCSS

No deficits
on the functional level of swallowing, saliva/secretions, food and liquids

No specific dysphagia therapy needed, swallowing and nutrition recommendations

Re-assess at follow-up visit

Deficits
on the functional level of swallowing, saliva/secretions, food and liquids

MDT-PD

Self estimated swallowing problems in daily living situations

No self estimated swallowing problems in daily living situations

No specific dyphagia therapy needed, swallowing and nutrition recommendations

Re-assess at follow-up visit

Laringoscopy with FEES

1st line treatment

Shared care with speech therapists for dysphagia training [1]

- Adoption of aids/assistance: eating and drinking aids, compensation strategies
- Nutritional (dietary) adaptations (texture modification of liquids and food IDDSI 0-7),
- Causal Therapy: relearning the physiologic swallowing act: training of the facial, tongue and chewing muscles, stimulation and mobilization of the oral cavity (thermic, proprioceptive and vibratory stimuli)
- Restorative therapy: posture modification, swallowing maneuvers (e.g. „chin down")
- Teaching swallowing-, breathing and coughing techniques
- Advising the caregivers to accompany eating

Poor symptomatic control

Good symptomatic control

Monitor at follow-up

2nd line treatment

Consider: PEG for severe dysphagia and esophageal dysmotility [1]

FEES: Flexible Endoscopic Evaluation of Swallowing.
IDDSI: International Dysphagia Diet Standardisation Initiative.
PEG: Percutaneous Endoscopic Gastrostomy.
UMSARS: Unified Multiple System Atrophy Rating Assessment.
MDT-PD: Munich Dysphagia Test- Parkinson Disease.
MUCSS: Munich- Copenhaghen Swallowing Screen.

[1] https://www.sciencedirect.com/science/article/pii/S1353802021001206?via%3Dihub

Dysarthria: speech therapists approach

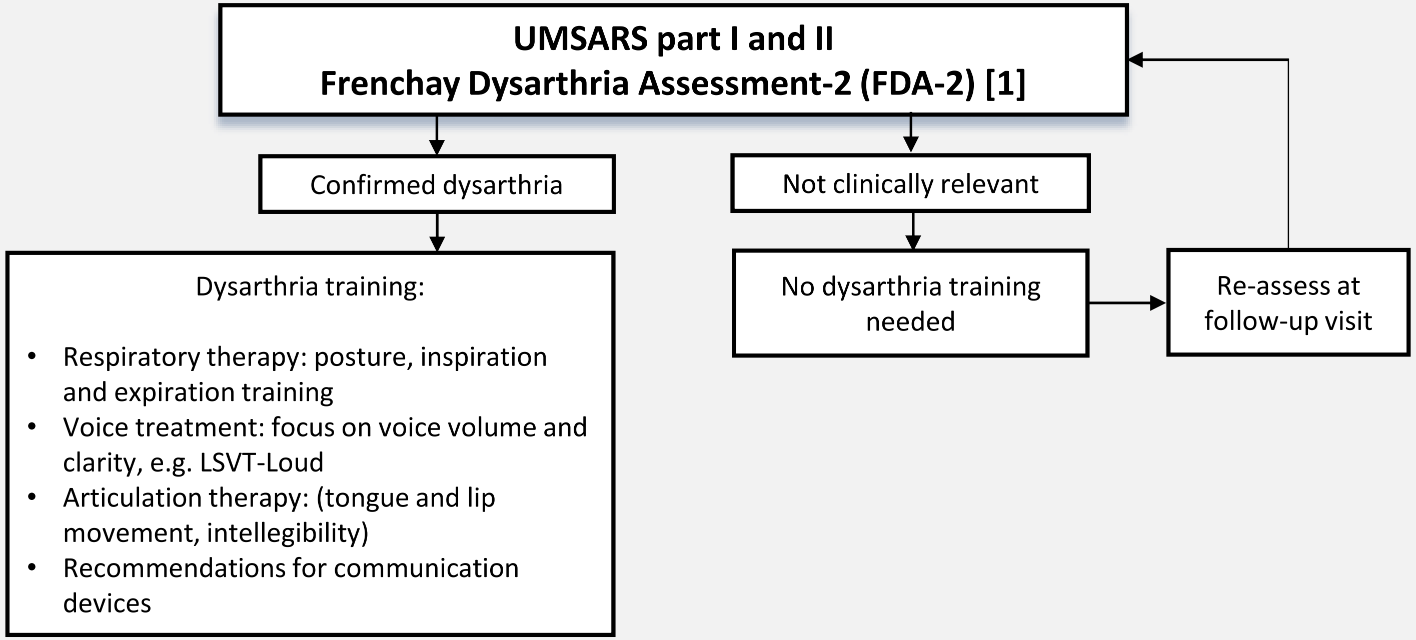

FDA-2: Frenchay Dysarthria Assessment- 2.
LSVT: Lee Silverman Voice Treatment.
[STANDARD ASSESSMENTS AND OUTCOME MEASURES FOR PATIENTS WITH DYSARTHRIA: A SCOPING REVIEWS STUDY | INTERNATIONAL JOURNAL OF ALLIED HEALTH SCIENCES (iium.edu.my)]

Cognition

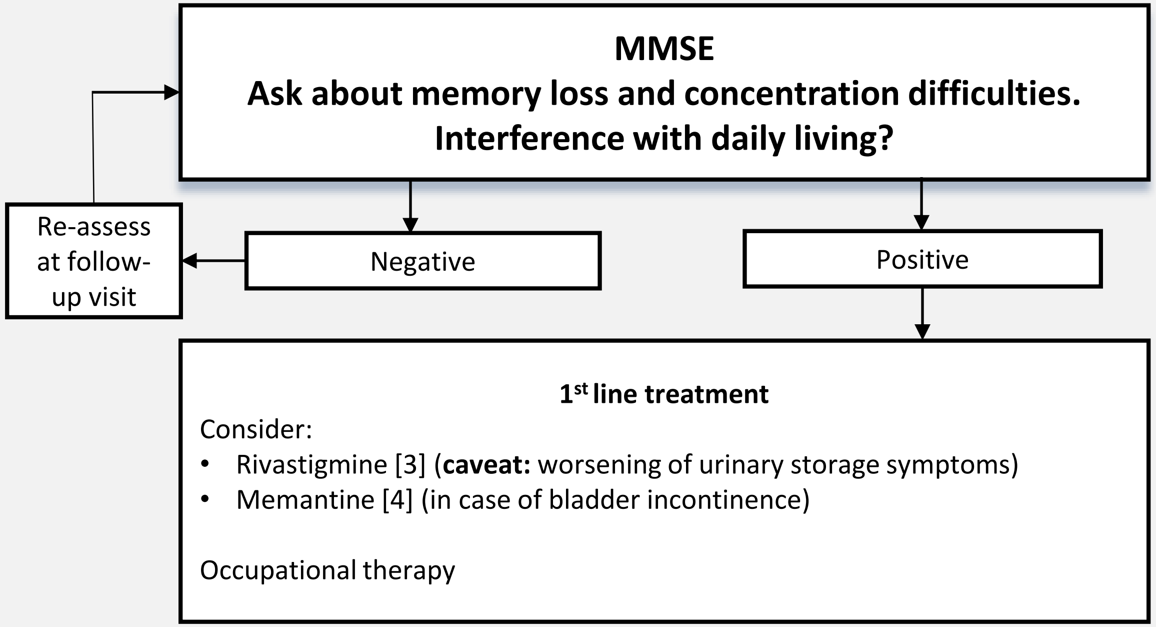

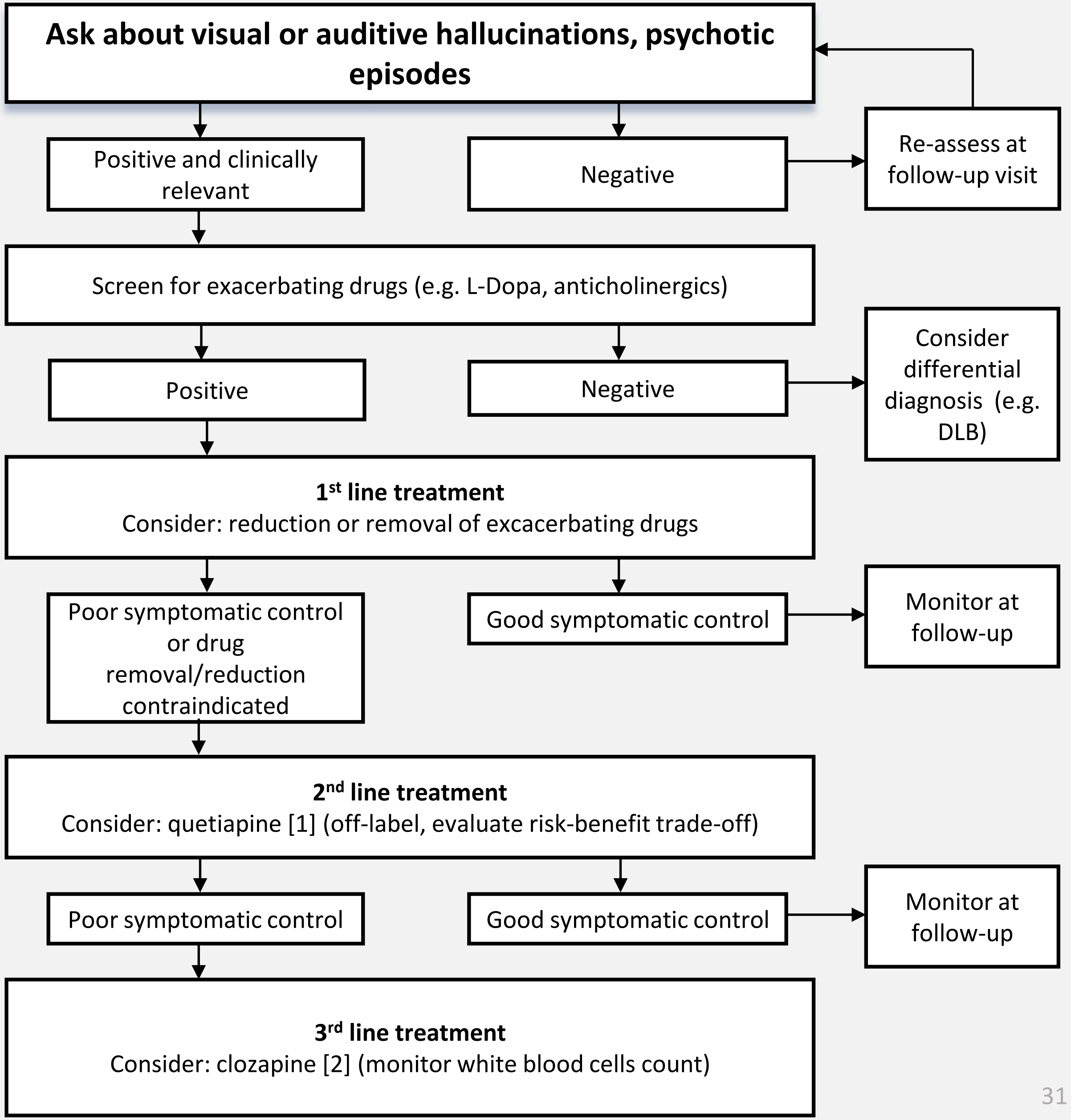

MMSE: Mini Mental State Examination.

[1] https://movementdisorders.onlinelibrary.wiley.com/doi/10.1002/mds.10374
[2] https://www.nejm.org/doi/full/10.1056/nejm199903113401003
[3] https://www.nejm.org/doi/full/10.1056/nejmoa041470
[4] https://www.sciencedirect.com/science/article/pii/S1474442209701462

Depression and Anxiety

HADS
Impact on daily living?

Score <11

Psychological counselling wished?

No → Re-assess at follow-up visit

Yes →

Psychological counselling, modular approach for tailored interventions:
1. Psychoeducation
2. Emotion regulation
3. Stress management
4. Support in adapting to change
5. Social support
6. Dealing with death and dying
7. Grief work
8. Existential issues

Good symptomatic control → Monitor at follow-up

Poor symptomatic control/new issues due to disease progression → 1st line treatment

Score ≥11

Multimodal treatment clinically indicated and wished?

No → Re-assess at follow-up visit

Yes →

1st line treatment
Shared care with psychiatrist

For depression:
- venlafaxin [1] (**caveat:** risk of worsening OH, supine hypertension and bladder disturbances)
- SSRI [1]

For anxiety:
- SSRI
- Consider: short-acting benzodiazepines (e.g. alprazolam on demand; **caveat**: monitor for worsening of stridor, sleep disordered breathing and OH)

Psychological counselling, modular approach for tailored interventions (see left panel)

Good symptomatic control → Monitor at follow-up

Poor symptomatic control/new issues due to disease progression →

2nd line treatment
Shared-care with psychiatrists for pharmacological adjustments, as needed
Psychologists: Introduction of new modules, based on individual needs

HADS: Hospital Anxiety and Depression Scale.
SSRI: Selective Serotonine Reuptake Inhibitors.
[1] https://www.ncbi.nlm.nih.gov/pmc/articles/PMC3324323/

Pain 1/2

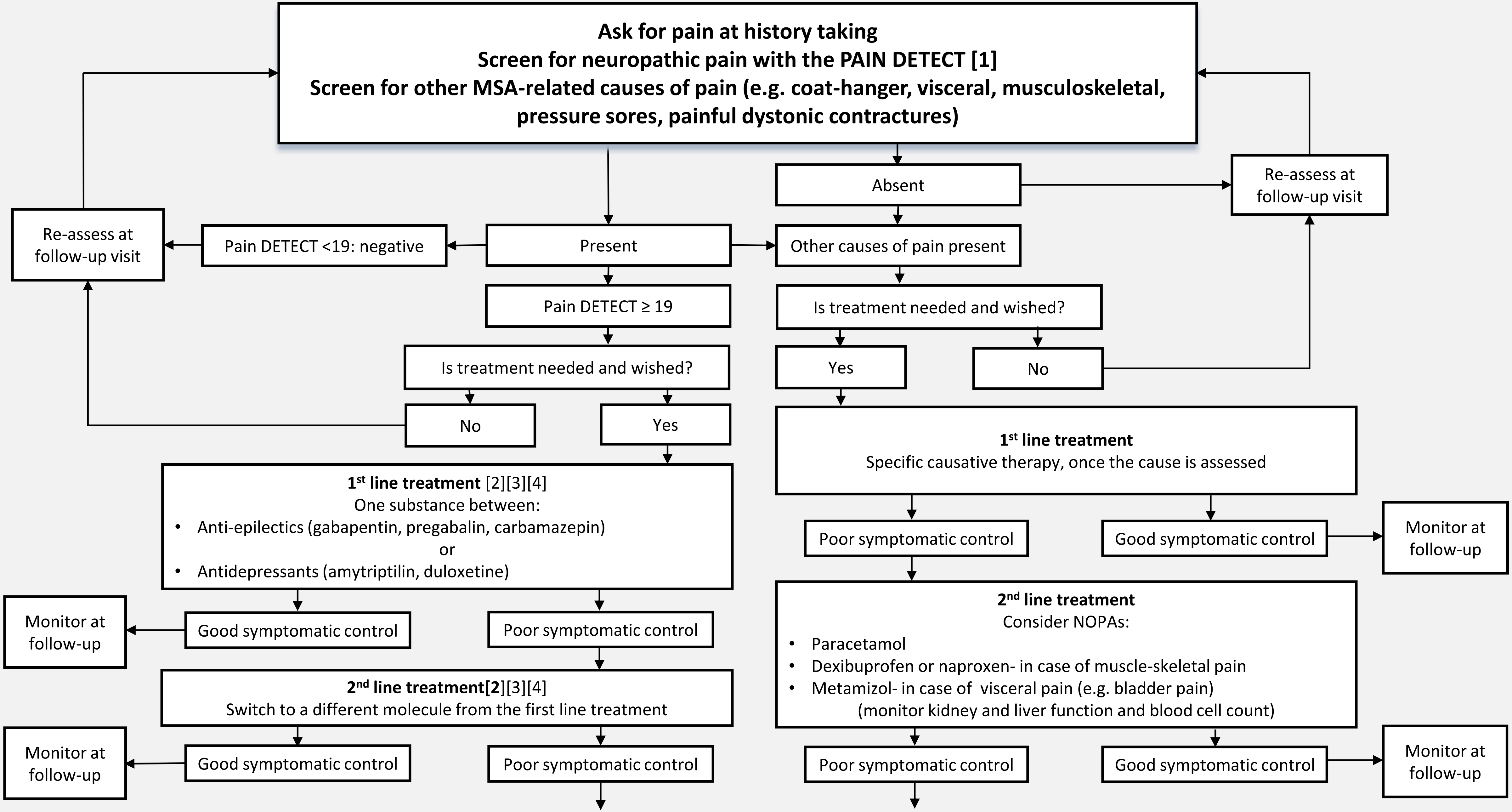
Ask for pain at history taking
Screen for neuropathic pain with the PAIN DETECT [1]
Screen for other MSA-related causes of pain (e.g. coat-hanger, visceral, musculoskeletal, pressure sores, painful dystonic contractures)
Absent
Re-assess at follow-up visit
Re-assess at follow-up visit
Pain DETECT <19: negative
Present
Other causes of pain present
Pain DETECT ≥ 19
Is treatment needed and wished?
Is treatment needed and wished?
Yes
No
No
Yes
1st line treatment
Specific causative therapy, once the cause is assessed
1st line treatment [2][3][4]
One substance between:
• Anti-epilectics (gabapentin, pregabalin, carbamazepin)
or
• Antidepressants (amytriptilin, duloxetine)
Poor symptomatic control
Good symptomatic control
Monitor at follow-up
Monitor at follow-up
Good symptomatic control
Poor symptomatic control
2nd line treatment
Consider NOPAs:
• Paracetamol
• Dexibuprofen or naproxen- in case of muscle-skeletal pain
• Metamizol- in case of visceral pain (e.g. bladder pain)
(monitor kidney and liver function and blood cell count)
2nd line treatment[2][3][4]
Switch to a different molecule from the first line treatment
Monitor at follow-up
Good symptomatic control
Poor symptomatic control
Poor symptomatic control
Good symptomatic control
Monitor at follow-up

Pain 2/2

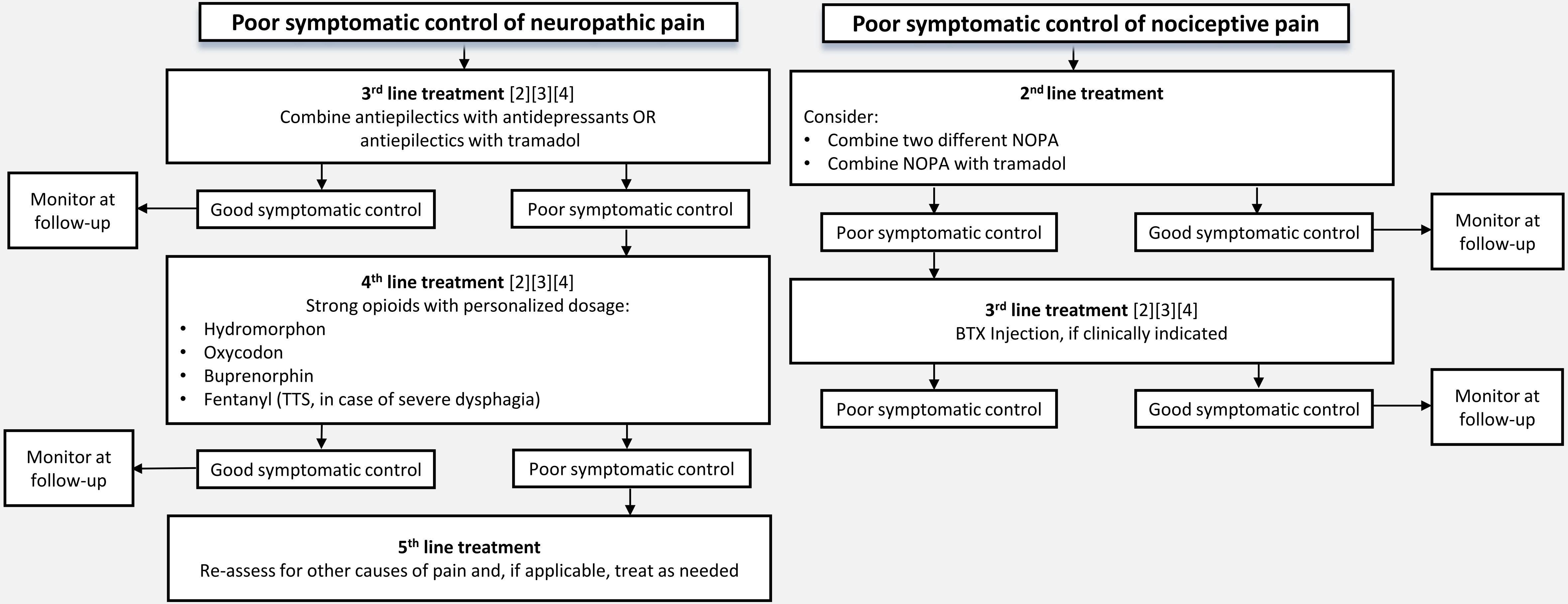

NOPA: Non Opiod Analgetic drugs.

[1] https://www.tandfonline.com/doi/abs/10.1185/030079906X132488
[2] https://journals.lww.com/pain/Fulltext/2015/04001/Pharmacotherapy_of_neuropathic_pain__which_drugs,.14.aspx
[3] https://academic.oup.com/painmedicine/article/20/Supplement_1/S2/5509427?login=true
[4] https://journals.lww.com/pain/Fulltext/2005/12050/Algorithm_for_neuropathic_pain_treatment__An.3.aspx

Palliative care 1/2

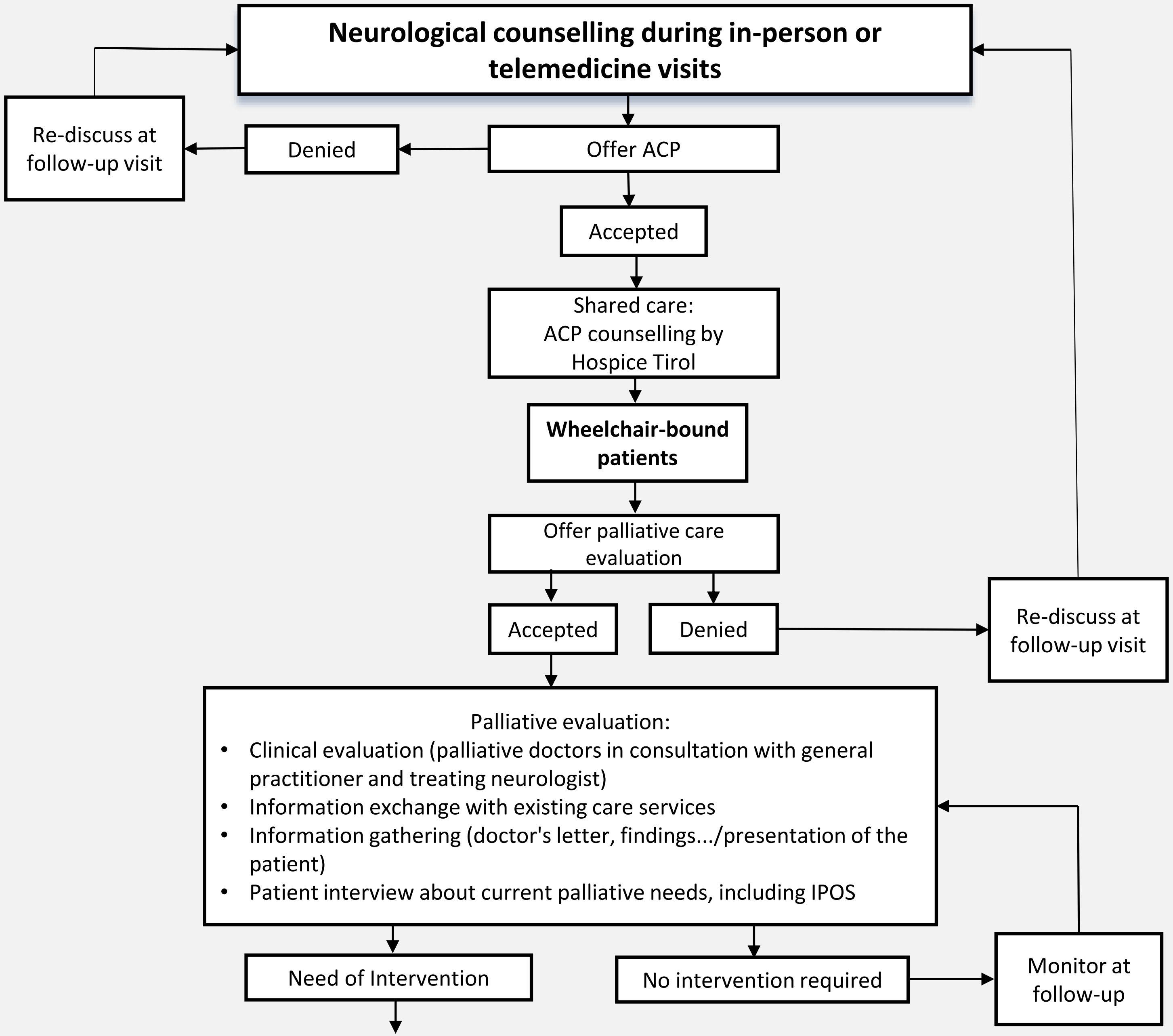

MPT: Mobile Palliative care Team.
IPOS: Integrated Palliative Outcome Scale.
ACP: Advance care planning.

Palliative care 2/2

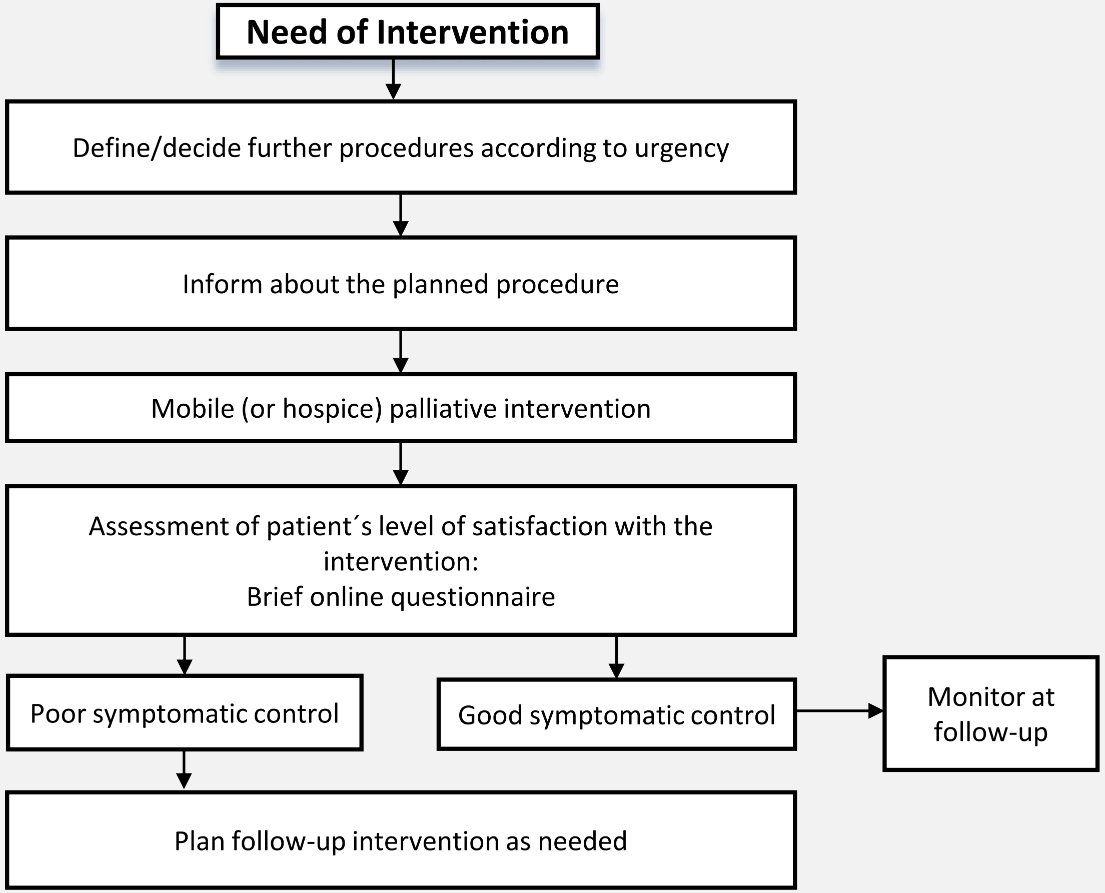

MPT: Mobile Palliative care Team.
SAPS: Short Assessment of Patient Satisfaction.

Social workers assistance

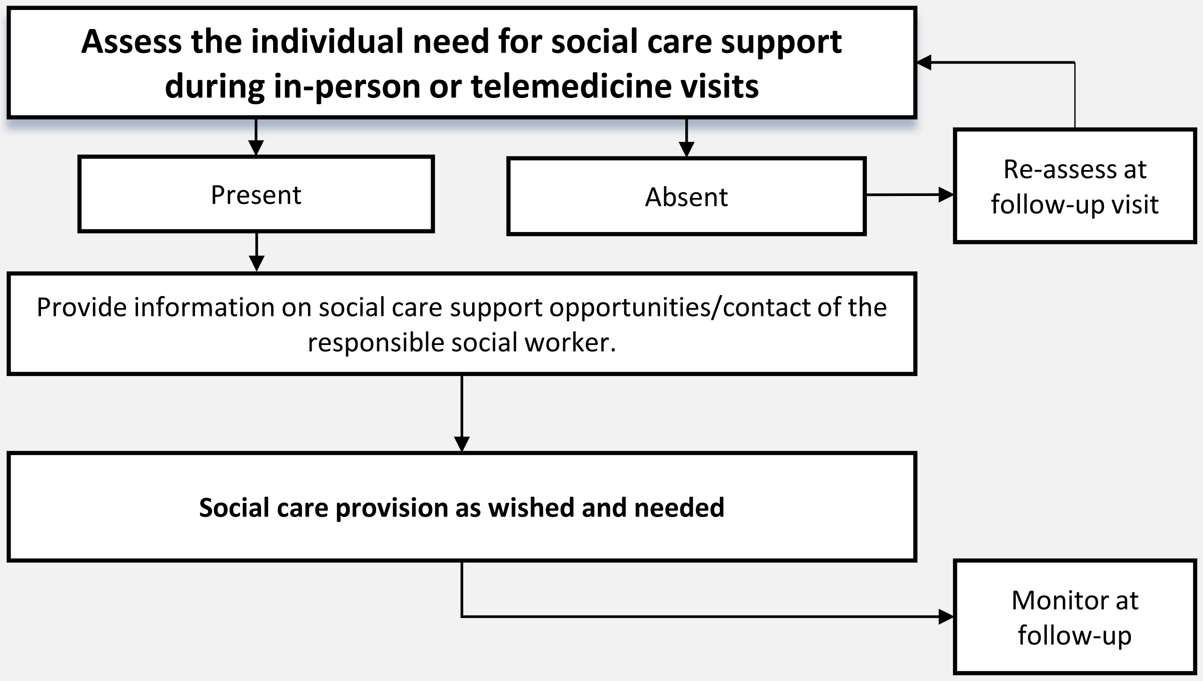

Abbreviations

ADL: Activities of daily living.

BBS: Berg Balance Scale.

BBT: Box and Blocks Test.

Bi PAP: Bifasic Positive Active Pressure.

BTX: Botulinum toxin.

CBT-I: Cognitive Behavioral Treatment- Insomnia.

COMPASS-31: Composite Autonomic Symptom Score-31.

CPAP: Continuous Positive Active Pressure.

ED: Erectile dysfunction.

EMG: Electromyography.

FAC: Functional Ambulation Categories.

FDA-2: Frenchay Dysarthria Assessment -2.

FEES: Flexible Endoscopic Evaluation of Swallowing.

HADS: Hospital Anxiety and Depression Scale.

IPOS: Integrated Palliative Outcome Scale.

ISI: Insomnia Severity Index.

MSA QoL: Multiple System Atrophy Quality of Life.

MDT-P: Munich Dysphagia Test- Parkinson Disease.

MMSE: Mini Mental State Examination.

MPT: Mobile Palliative care Team.

MUCSS: Munich- Copenhaghen Swallowing Screen.

NHPT: Nine Hole Pipe Test.

NOPAs: Non Opiod Analgetic drugs.

OH: Orthostatic Hypotension.

OSA: Obstructive Sleep Apnoea.

OSA-DLS: Occupational Self Assessment- Daily Living Scale.

PEG: Percutaneous Endoscopic Gastrostomy.

Q-SART: Quantitative Sudomotor Axon Reflex Testing.

RBD-I- 5Q: Innsbruck REM sleep behaviour inventory- 5 questions.

SAPS: Short Assessment of Patients Satisfaction.

SARA: Scale of the Assessment and Rating of Ataxia.

SSRI: Selective Serotonin Re-uptake Inhibitors.

TCT: Trunk Control Test.

TUG: Timed Up & Go.

UMSARS: Unified Multiple System Atrophy Rating Assessment.

VPSG: Video polysomnography.

10MWT: 10 Meters Walking Test.

24h- ABPM: 24 hours Ambulatory Blood Pressure Monitoring.
