# Supplementary material for: MeDeMSA care study protocol: developing personalized best medical care with integrated mobile palliative and telemedicine support for individuals with multiple system atrophy
Source: J Neural Transm (Vienna). 2025 May 24;133(5):903–17. doi: 10.1007/s00702-025-02933-z (PMC13216168; doi:10.1007/s00702-025-02933-z)
Supplement: Supplementary file 2 — Supplementary file2 (PDF 4748 KB) [file 702_2025_2933_MOESM2_ESM.pdf]

MeDeMSA Care study protocol – Supplementary Material 2

Physiotherapy Operational Protocol

Table 1 MeDeMSA Care Physiotherapy Operational Protocol Level 1 consists of basic physiotherapy exercises designed for individuals with greater disability. Level 2 includes more difficult exercises intended for individuals with lower levels of disability, as determined by the functional assessment during the in-person visits. An illustrated exercise catalogue is provided hereinafter.

	Level	Test results	Therapy content
**1. Transfer**	Level 1	FAC < 3 P.<br>SARA ≥ 1 P.<br>TCT ≤ 50 P.<br>BBS ≤ 45 P.	Transfer in bed<br>- Slide upwards while lying on the back<br>- Roll from the back to the side position<br>- Sit up to the edge of the bed from a lying position<br>- Slide forward at the edge of the bed<br>- Slide sideways at the edge of the bed<br>- Stand up from the bed (lean body forward, if necessary, with cues), using hands (e.g., on knees or a chair/table)
	Level 2	FAC ≥ 3 P.<br>SARA 0 P.<br>TCT > 50 P.<br>BBS > 45 P.	Transfer from chair or floor<br>- Stand up without using hands, with cues<br>- Stand up from lower & softer surfaces (e.g., sofa)<br>- Transfer from chair to chair (including getting in/out of a car)<br>- Turn around while standing<br>- Lie down & get up from the floor using hands (this exercise may require assistance)

	Level	Test results	Therapy content
2. Movement & Posture	Level 1	FAC < 3 P.<br>TUAG ≥ 20 sec.<br>BBS ≤ 45 P.<br>TCT ≤ 50 P.	While lying in bed<br>- Lie flat on the back, optionally with arms resting to the side or stretched above the head<br>- Lie flat on the stomach, optionally stretching the neck and back, and positioning the hands under the chin<br>- Lie flat on the back, bend knees in the supine position and rotate to the side<br><br>While sitting<br>- Stretch the back by alternating bending downwards to touch the ankles and stretching arms to the ceiling<br>- Large arm swings while sitting<br>- Rotate the back with arm swings to the side<br>- Stretch the shoulder-neck area by tilting head sideways
	Level 2	FAC ≥ 3 P.<br>TUAG < 20 sec.<br>BBS > 45 P.<br>TCT > 50 P.	Standing and lying<br>- Stretch chest muscles against a door frame or wall<br>- Lean back, head, and hips against the wall, and try to raise arms overhead while keeping them against the wall (avoiding an arched back)<br>- Lie flat on the stomach, then push up as high as possible with the arms into a back extension
3. Circulation	Level 1	Severe orthostatic intolerance	Within the bed<br>- Ankle pumps<br>- Bend and stretch legs<br>- Single leg cycling<br>- Flex and extend wrists<br>- Stretch arms toward the ceiling and make circular movements

	Level	Test results	Therapy content
3. Circulation	Level 2	Mild orthostatic intolerance	While sitting and standing:<br>- Large arm swing movements<br>- Air boxing forward<br>- Sit-to-stand exercises<br>- Short walks
4. Balance	Level 1	FAC < 3 P.<br>SARA ≥ 1 P.<br>TCT ≤ 50 P.<br>BBS ≤ 45 P.	While sitting<br>- Free sitting with/without the use of hands<br>- Free sitting, reaching far targets left & right<br><br>While standing<br>- Standing with hand support (holding onto kitchen worktop, railing, windowsill, sink), optionally with a wider stance
	Level 2	FAC ≥ 3 P.<br>SARA 0 P.<br>TCT > 50 P.<br>BBS > 45 P.	Dynamic standing<br>- Standing without hand support (near holding options) under supervision<br>- Move an object (e.g., water bottle) across a table left & right<br>- Pick up an object from the floor<br>- Move an object up & down from a shelf<br>- Step forward/sideways/backward

	Level	Test results	Therapy content
**5. Strength**	Level 1	Significant general strength deficit present	3x5 repetitions<br>- Back: lie on the back, bend knees, and lift pelvis<br>- Abdomen: lie on the back, slide hands along thighs up & down toward knees<br>- Legs: squats (with support, optionally sitting down)<br>- Arms: stretch water bottles toward the ceiling (starting at shoulder height)
	Level 2	Minor general strength deficit present	3x15 repetitions<br>- Back: lie on the back, bend knees, and lift pelvis<br>- Abdomen: lie on the back, slide hands along the thighs toward the knees and backward<br>- Legs: squats (with support, optionally sitting down)<br>- Arms: stretch water bottles toward the ceiling (starting at shoulder height)
**6. Gait**	Level 1	FAC < 3 P.<br>TUAG ≥ 20 sec.<br>10 MWT ≥ 12,5 sec.<br>SARA 12-26 P.	Assisted walk<br>- Few variations, short distances, more breaks<br>- Step on the place forward & backward with support and assistance<br>- Short walks with assistance and walking aids<br>- Short walks with regular sitting breaks<br>- Count steps on daily routes (e.g., bed to WC) and try to reduce the number of steps required (take larger steps + RAS)<br>- Record the maximum walking time before a sitting break is needed<br>- Freezing-of-gait training with visual and auditory cues

	Level	Test results	Therapy content
6. Gait	Level 1	FAC < 3 P.<br>TUAG ≥ 20 sec.<br>10 MWT ≥ 12,5 sec.<br>SARA 12-26 P.	Minimal assistance<br>- Walk under close supervision with aids<br>- Walk short distances without assistance (e.g., along a wall)<br>- Walk longer distances with standing breaks or fewer sitting breaks<br>- Focus on step width/length/height, posture, arm swing, and speed
	Level 2	FAC ≥ 3 P.<br>TUAG < 20 sec.<br>10 MWT < 12,5 sec.<br>SARA 1-11 P.	Without assistance: introduce more variations, bigger distances, less breaks<br>- Walk with start-stop commands (with cues)<br>- Walk around objects (with cues)<br>- Walk on various surfaces (grass, forest ground, cobblestones, slopes)<br>- Walk over obstacles<br>- Walk with dual tasks (counting, reciting, calculating)<br>- Walk forward, backward & sideways along a line<br>- Long walks & outdoor walks

FAC = Functional Ambuliation Categories; SARA = Scale of the Assessment and Rating of Ataxia; TCT = Trunk Control Test; BBS = Berg Balance Scale; TUG = Timed-Up-and-Go-Test; 10MWT = 10-meter walk test.

MeDeMSA Care illustrated Physiotherapy exercise catalogue

1. Transfer (Level 1)....9
Slide up lying down....9
Exercise transfer in the supine position....10
Exercise transfer in lateral position with visual cue....11
Sit up to the edge of the bed....12
Slide forward to the edge of the bed....13
Sliding sideways along the edge of the bed....14
Standing up from the bed using a support....15
Standing up from the bed without support....16
1. Transfer (Level 2)....17
Standing up from the chair....17
Turning around while standing....18
Lay on the floor....19
2. Movement & Posture (Level 1)....20
Stretched supine position....20
Stretched prone position....21
Lower body rotation while lying down....22
Flexion and extension of the back....23
Large swings....24

Upper body rotation with lateral arm swing 25
Stretching the shoulder/neck muscles 26
2. Movement & Posture (Level 2) 27
Stretching chest muscles on the wall 27
Stretching against the wall 28
Back extension from prone position 29
3. Circulation (Level 1) 30
Foot tapping 30
Pull up and stretch legs 31
Single leg cycling 32
Bend & stretch wrists 33
Stretch your arms to the ceiling 34
3. Circulation (Level 2) 35
Arm swings in seated position 35
Boxing in the air 36
Stand up and sit down 37
4. Balance (Level 1) 38
Sit without support 38
Sit without support while moving the arms 39
Standing with support 40

4. Balance (Level 2) 41
Standing without support 41
Standing without support while moving your arms 42
Stand up while lifting an object from the floor 43
Steps forwards/sideways/backwards with support 44
5. Strength (Level 1) 45
Back muscles 45
Abdominal muscles 46
Leg muscles 47
Arm muscles 48
5. Strength (Level 2) 49
Back muscles 49
Abdominal muscles 50
Leg muscles 51
Arm muscles 52
6. Gait (Level 1) 53
Walking with assistance 53
6. Gait (Level 2) 54
Walking without assistance 54
7. General recommendations 55

1. Transfer (Level 1)

Slide up lying down

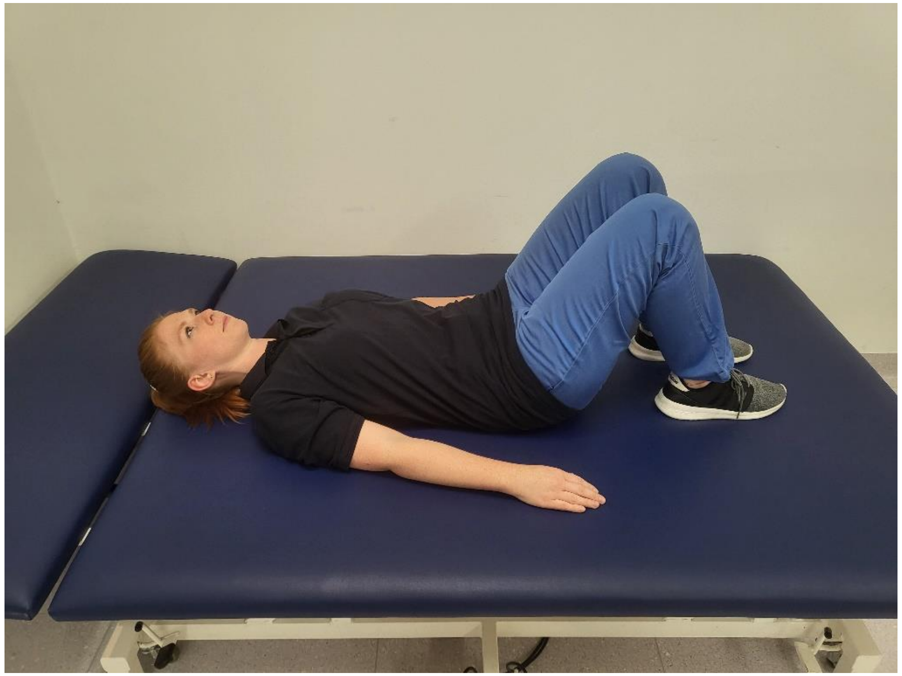

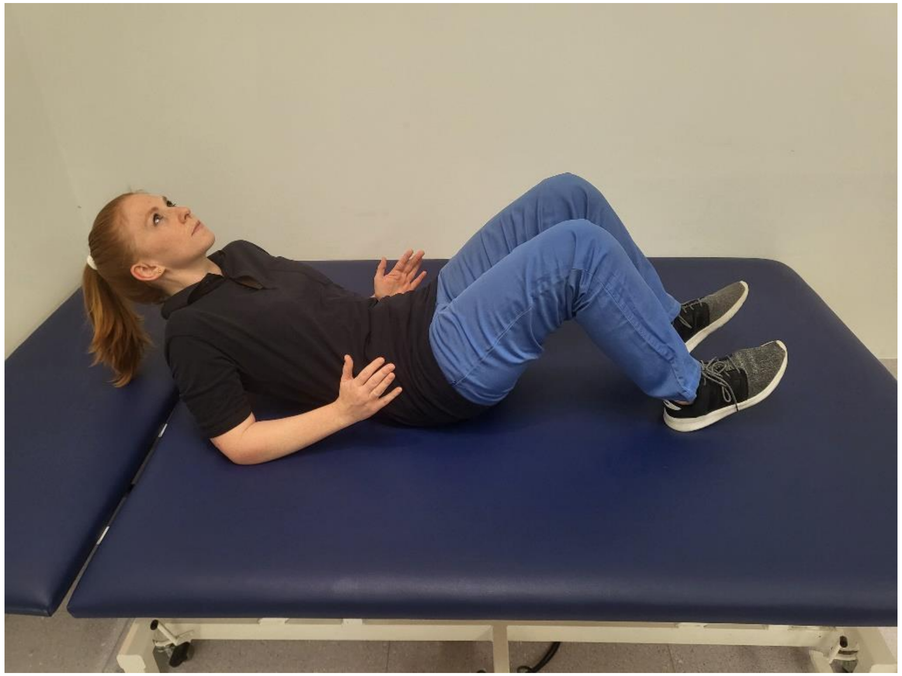

While lying down (e.g., bed or sofa), raise your head:

- While lying on the back, pull up your legs and bend your knees
- Press your elbows and heels firmly into the support
- Raise head
- Lift your buttocks and push your body upwards

Slide to the side:

- Same procedure (see left)
- Lift the buttocks and place them to the side
- And move body to the side

1. Transfer (Level 1)

Exercise transfer in the supine position

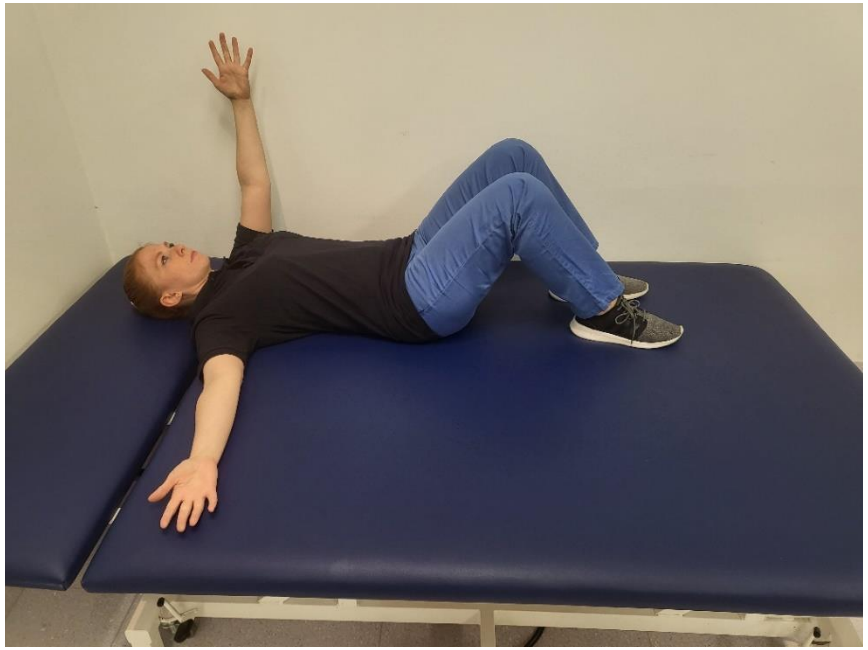

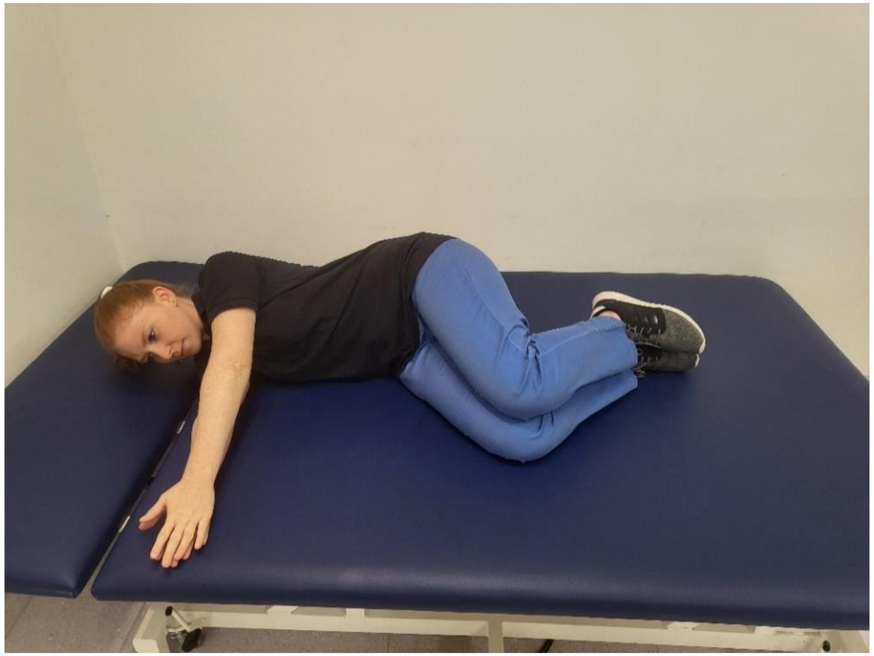

Turn onto your side while lying down:

- While lying on the back, pull up your legs and bend your knees
- Stretch your arms out to the side of your body
- Turn the knee to the desired side
- Bring both hands together and turn them to the side

1. Transfer (Level 1)

Exercise transfer in lateral position with visual cue

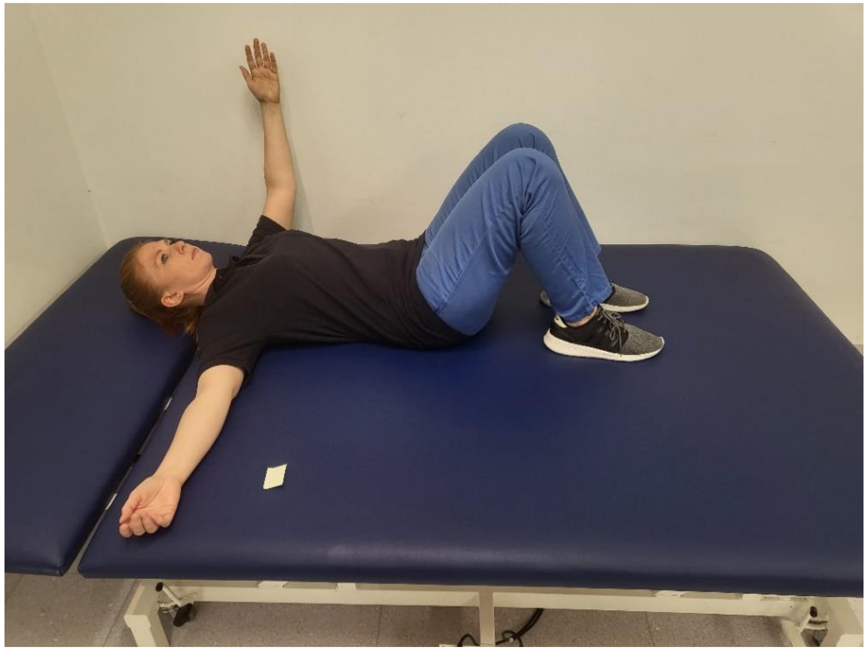

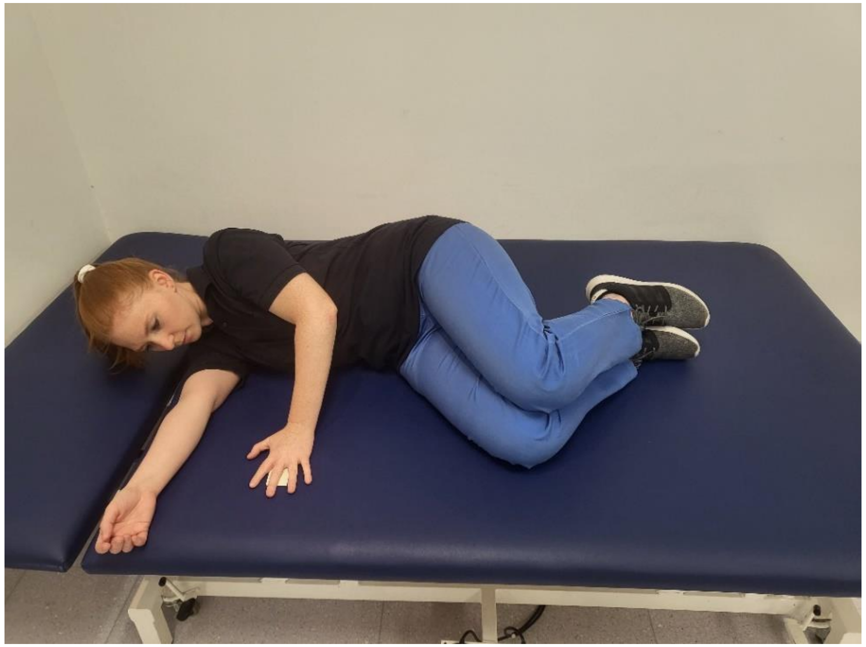

Turn on your side while lying down with a visible cue:

- Place a visible cue on the desired side (handkerchief, smartphone, sock: the more striking the color - the better)
- Pull up your legs, bend your knees
- Stretch your arms out to the side of your body
- Turn the knee to the desired side
- Move the upper hand to the cue and turn it to the side

Variation: count out loud and turn over by 3: 1, 2, 3 turn over

1. Transfer (Level 1)

Sit up to the edge of the bed

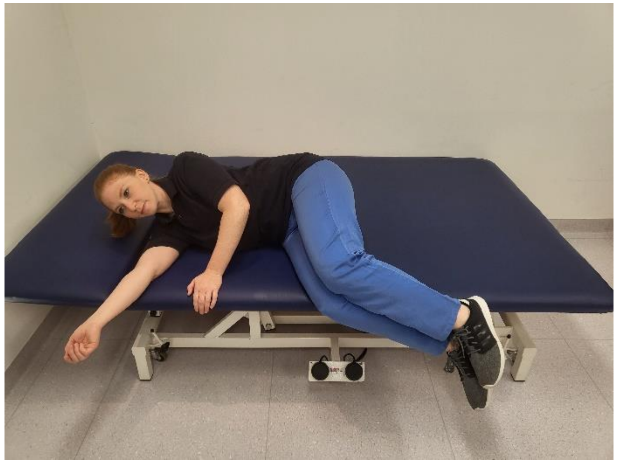
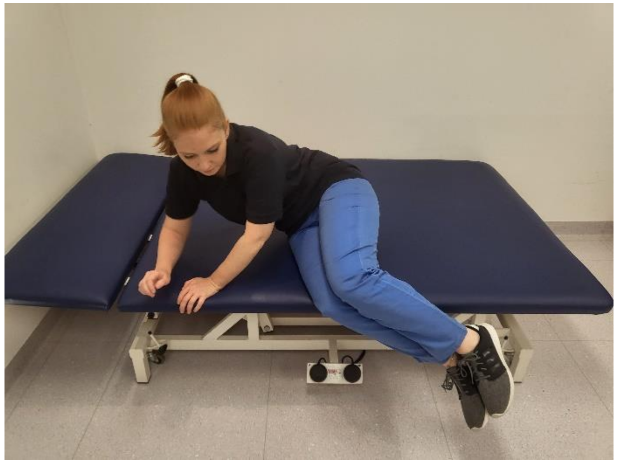
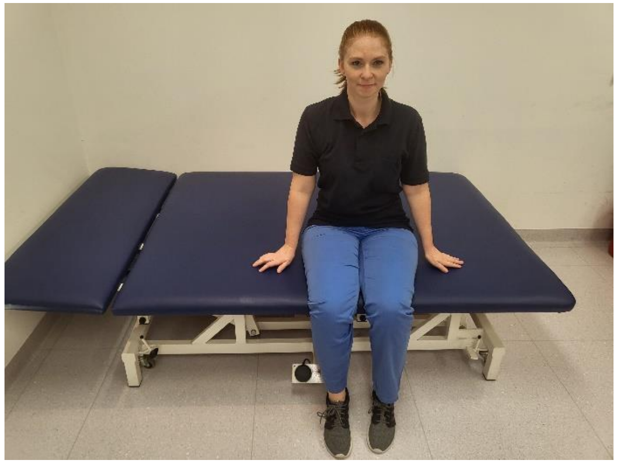

Sit up from a lying position:

- Lying on your side, pull your legs and knees up and push your feet over the edge of the bed
- Support yourself with the edge of the bed with your lower elbow and upper hand
- Lean from the lower elbow onto the hand to raise your trunk and sit up

Tip: If you feel dizzy or have blood pressure problems when changing your body position, please perform the exercise slowly and remain seated for a while, then lay down again if necessary.

1. Transfer (Level 1)

Slide forward to the edge of the bed

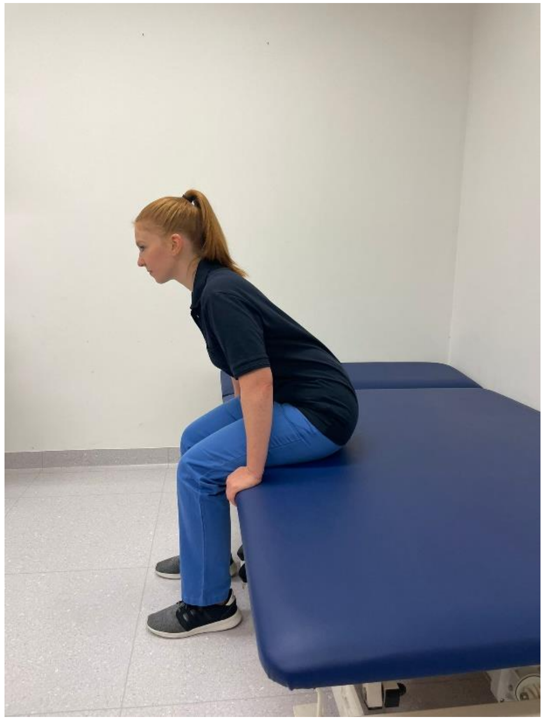

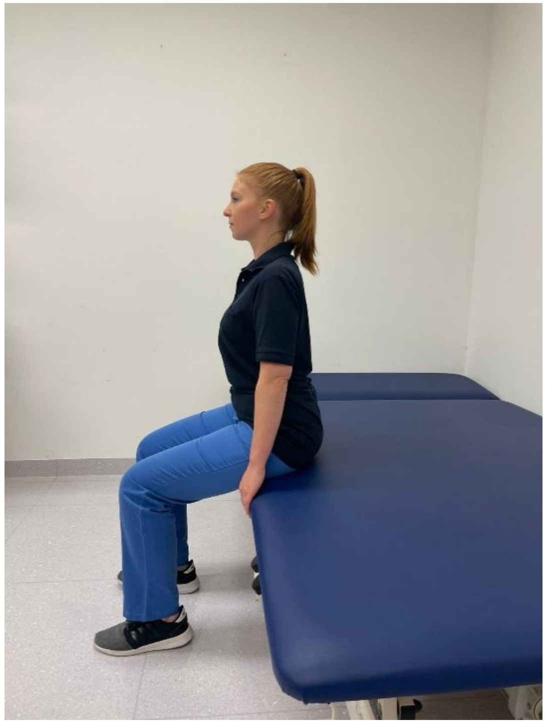

Slide to the edge of the bed while sitting:

- Hold on to the edge of the bed with your hands
- Bend your upper body forward and raise your buttocks
- Push the buttocks forward
- If necessary, repeat several times until the desired sitting position is reached

1. Transfer (Level 1)

Sliding sideways along the edge of the bed

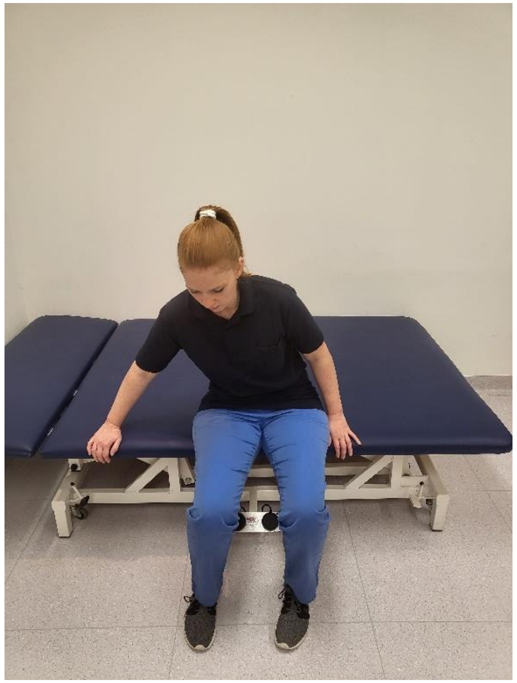

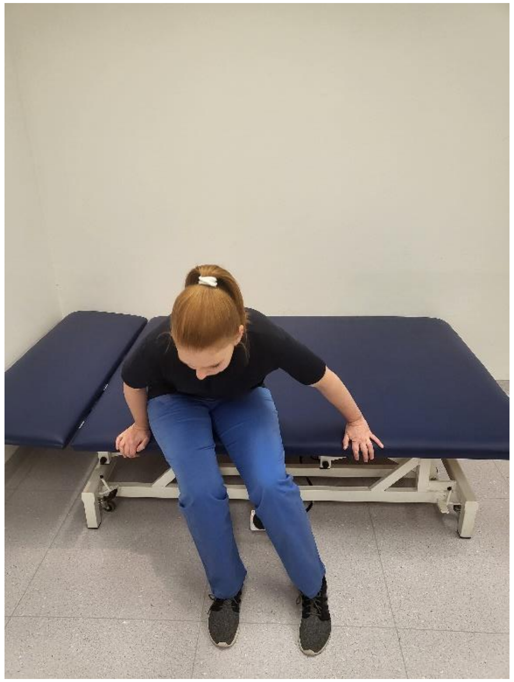

Slide to the left and right along the edge of the bed:

- Hold on to the edge of the bed with your hands and move one hand sideward to the desired side
- Bend your upper body forward and raise your buttocks
- Move your buttocks to the side
- If necessary, repeat several times until the desired sitting position is reached

1. Transfer (Level 1)

Standing up from the bed using a support

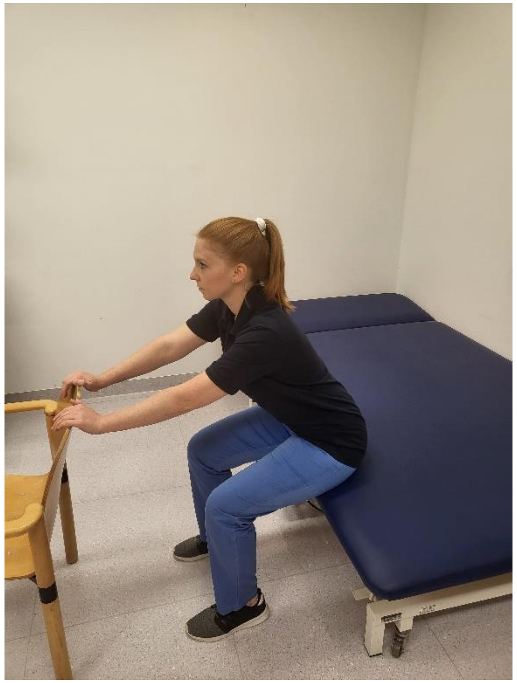
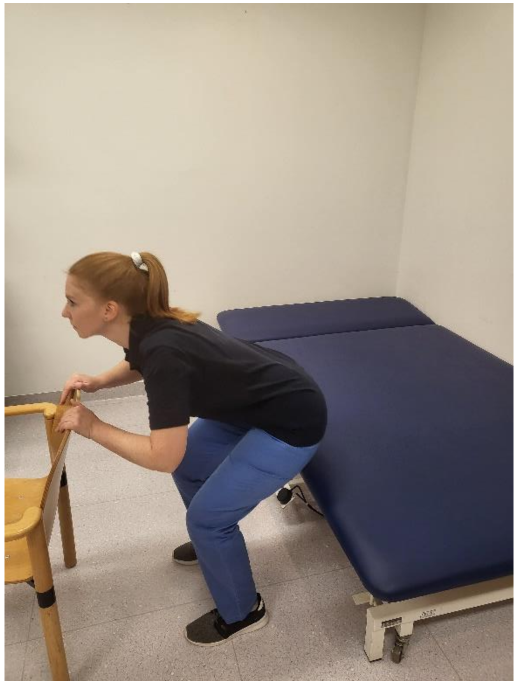
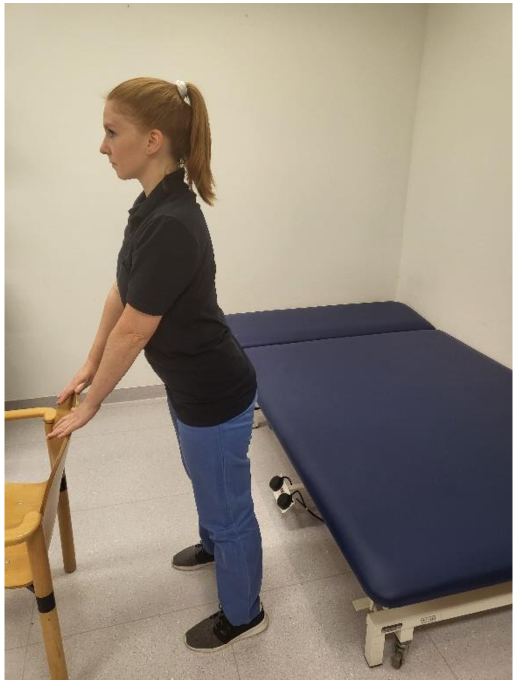

Stand up:

- Sit on the edge of the bed and hold on to a fixed object in front of you with your hands (chair, table, windowsill, walker)
- Place your feet wide apart and place your heels far back (as close as possible to the bed)
- Bring the upper body forward with momentum, lift the buttocks and straighten the body
- If necessary, try several times until the upper body is far enough forward
- Repeat at least 5-10 times

Tip: If you feel dizzy or have blood pressure problems when changing your body position, please perform the exercise slowly, hold on tight and stand still for a while, then sit down again if necessary.

1. Transfer (Level 1)

Standing up from the bed without support

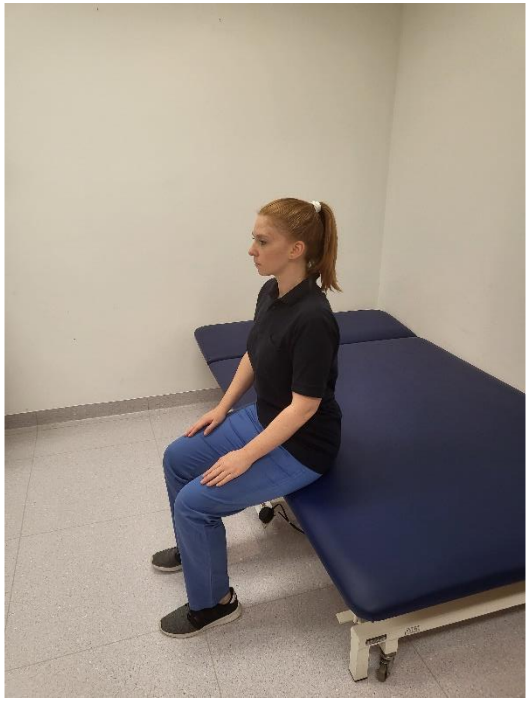

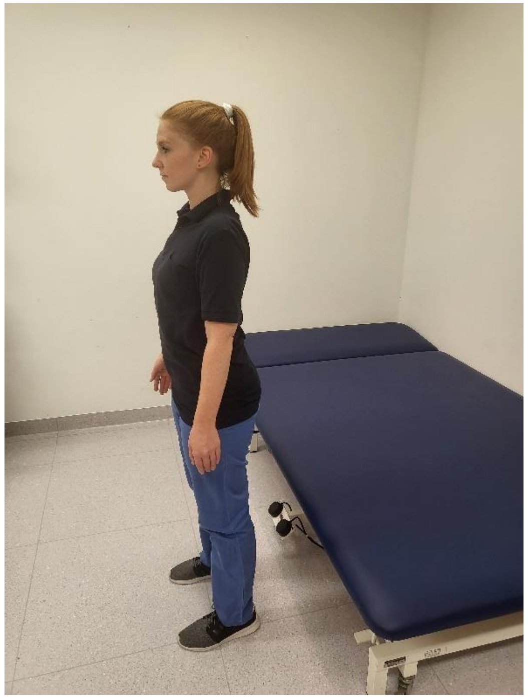

Stand up:

- Sit on the edge of the bed, place hands on your thighs, if necessary
- Keep your feet wide apart and place your heels far back (as close as possible to the bed)
- Bring the upper body forward with momentum, lift the buttocks and straighten the body
- If necessary, try several times until the upper body is far enough forward
- Repeat at least 5-10 times

Tip: If you feel dizzy or have blood pressure problems when changing your body position, please perform the exercise slowly and remain standing for a while, then sit down again if necessary.

1. Transfer (Level 2)

Standing up from the chair

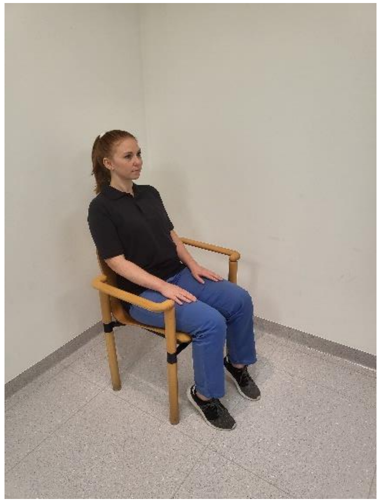
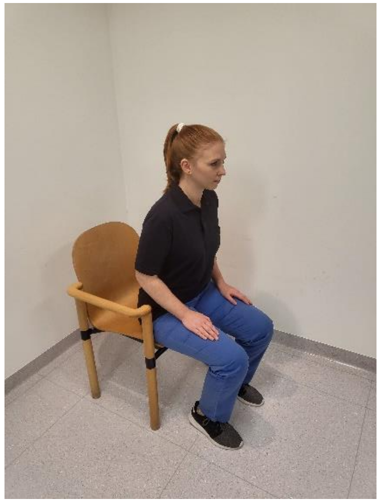
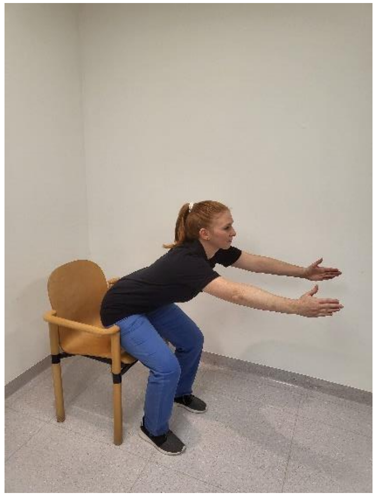
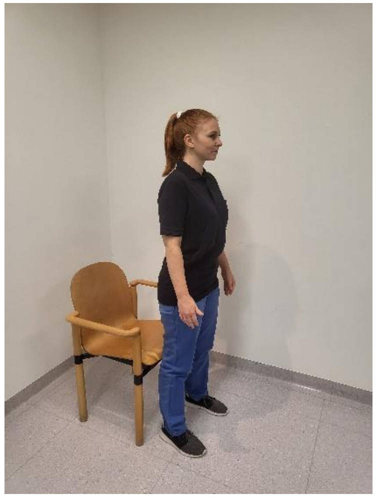

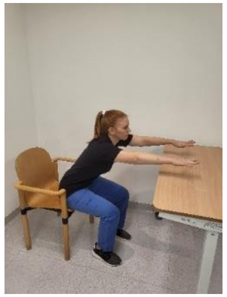

Stand up:

- Slide forward on the chair, place hands on your thighs if necessary
- Keep your feet wide apart and place your heels far back under the chair
- Bring the upper body forward with momentum, lift the buttocks and straighten the body
- If necessary, stretch your hands far forward and try several times until your upper body is far enough forward
- Repeat at least 5-10 times

Tip: If you feel dizzy or have blood pressure problems when changing your body position, please perform the exercise slowly and remain standing for a while, then sit down again if necessary.

Variant: Standing up in front of table/chair back for safety; standing up from lower & softer seating surfaces (sofa); getting in/out of car; changing from chair to chair.

1. Transfer (Level 2)

Turning around while standing

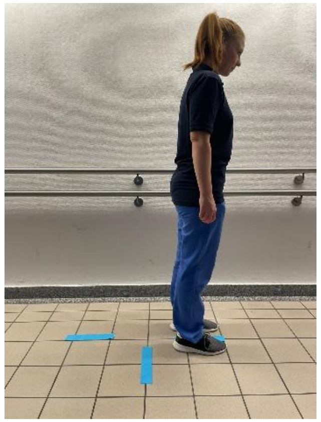
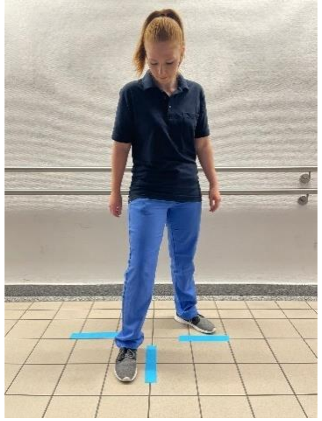
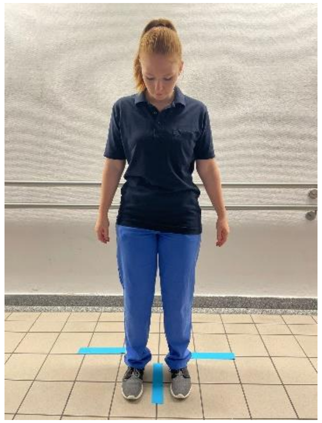
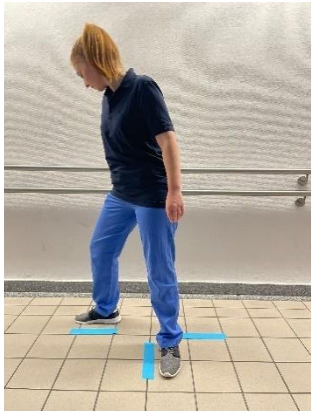
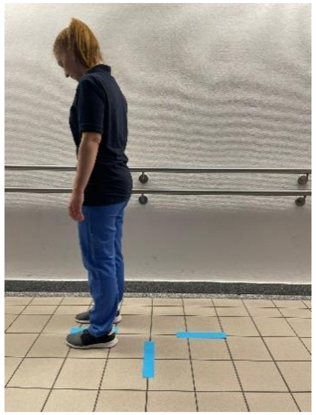

Turn around while standing:

- Take partial steps to the side until you reach the desired position
- Lift your feet well into the air, avoid dragging your feet
- A few big steps are better than many small ones
- Repeat at least 3-5 times per side

Variant: Turn around with 6 large steps while counting loud

- Climb over or onto visible signs at every step (tile joints, colored adhesive strips)

Attention: Safety first, please practice under the supervision of another person.

1. Transfer (Level 2)

Lay on the floor

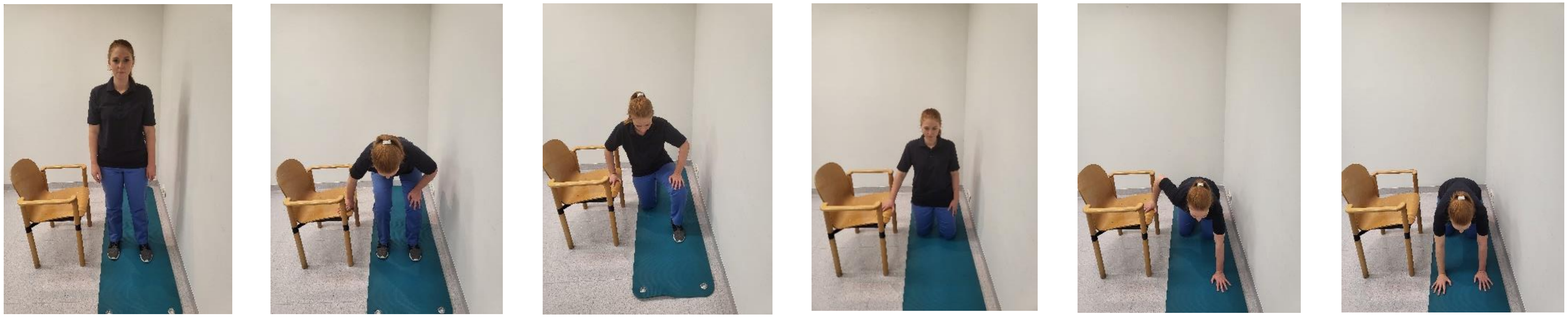

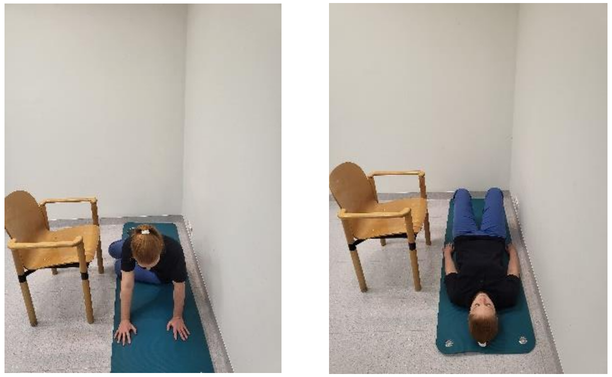

Lay on the floor:

- Stand next to an object to hold on to (chair, bed, table)
- Bend your upper body down and bend one knee backwards and kneel on it
- Move the other knee next to it
- Support yourself on the floor with one hand, the other follows from the chair (etc.) to the floor
- Tilt your buttocks to one side towards the floor and sit sideways
- Support yourself with your hand and slowly turn onto your back
- Follow the instructions backwards to get up from the floor

Attention: Safety first, please practice under the supervision of another person, this exercise requires strength and agility.

2. Movement & Posture (Level 1)

Stretched supine position

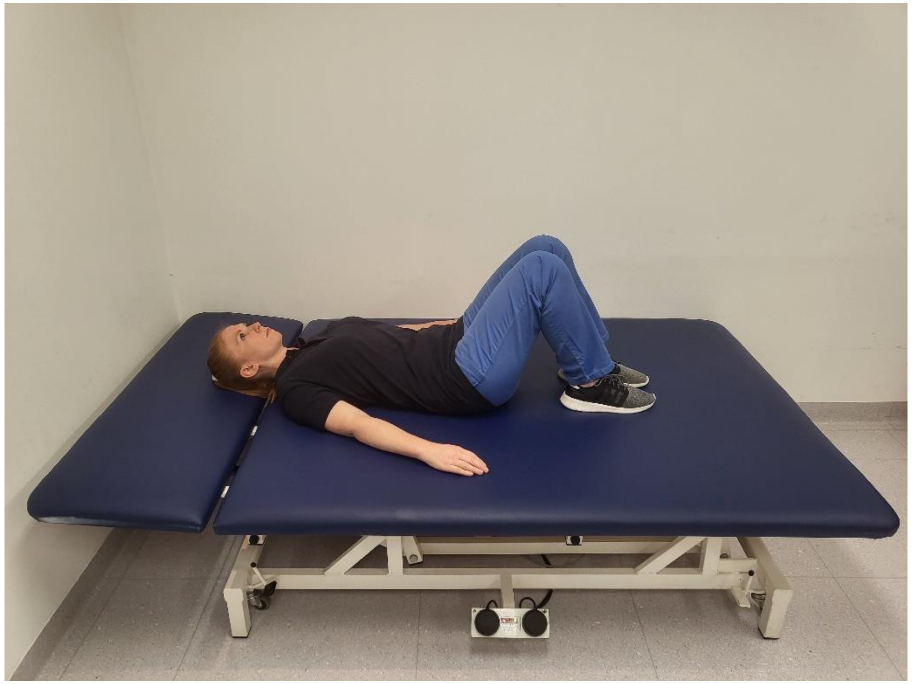

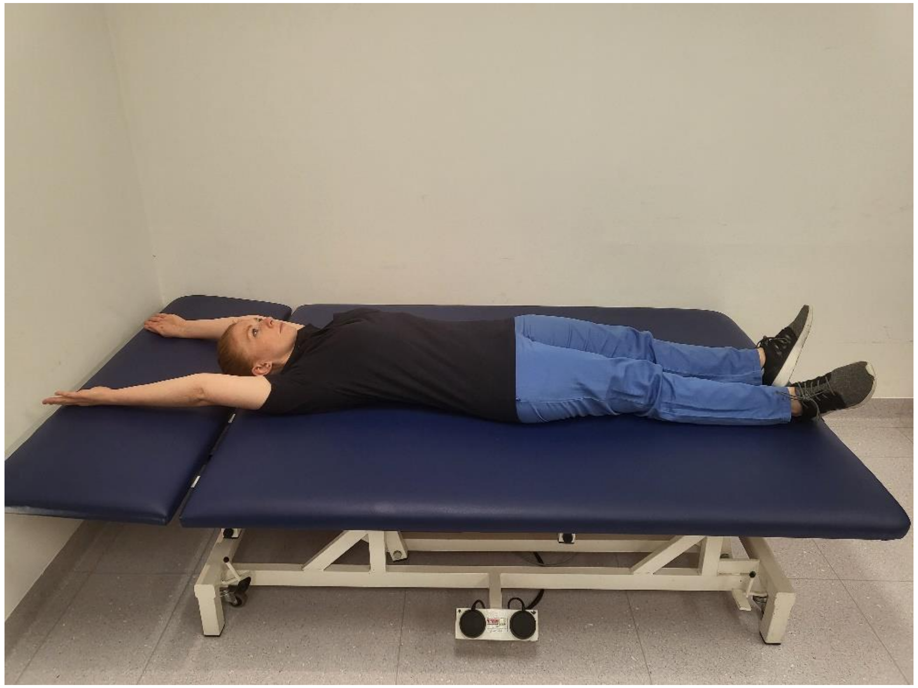

Stretching in supine position:

- Lie in the middle of the bed on your back
- Stretch out your arms and legs as far as possible
- Hold the stretching position for few seconds, repeat 3 times

Variation: Stretch your arms to the side or, if possible, above your head; if necessary, support your arms or the back of your knees with pillows.

2. Movement & Posture (Level 1)

Stretched prone position

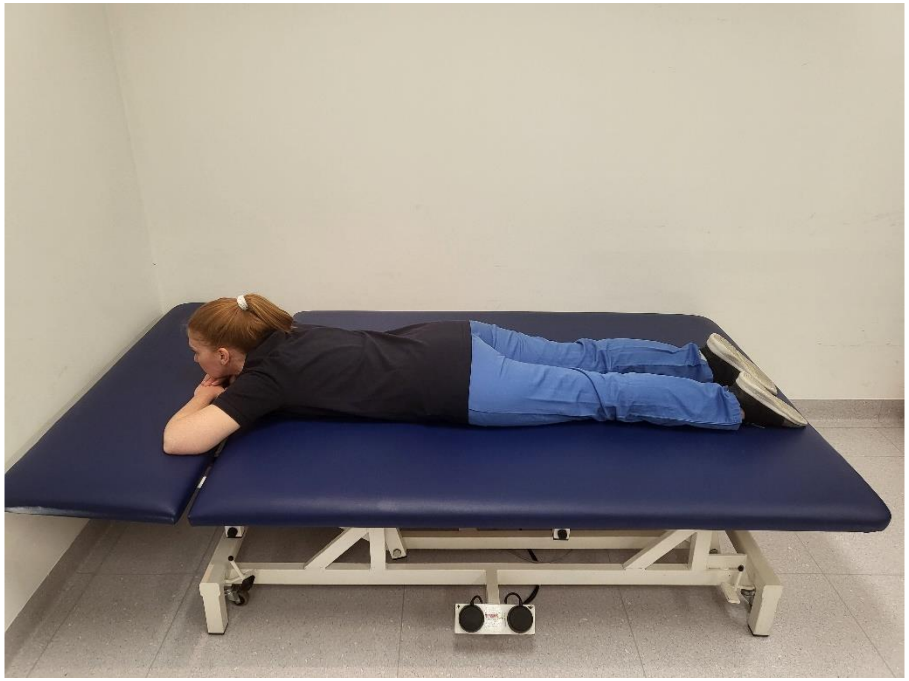

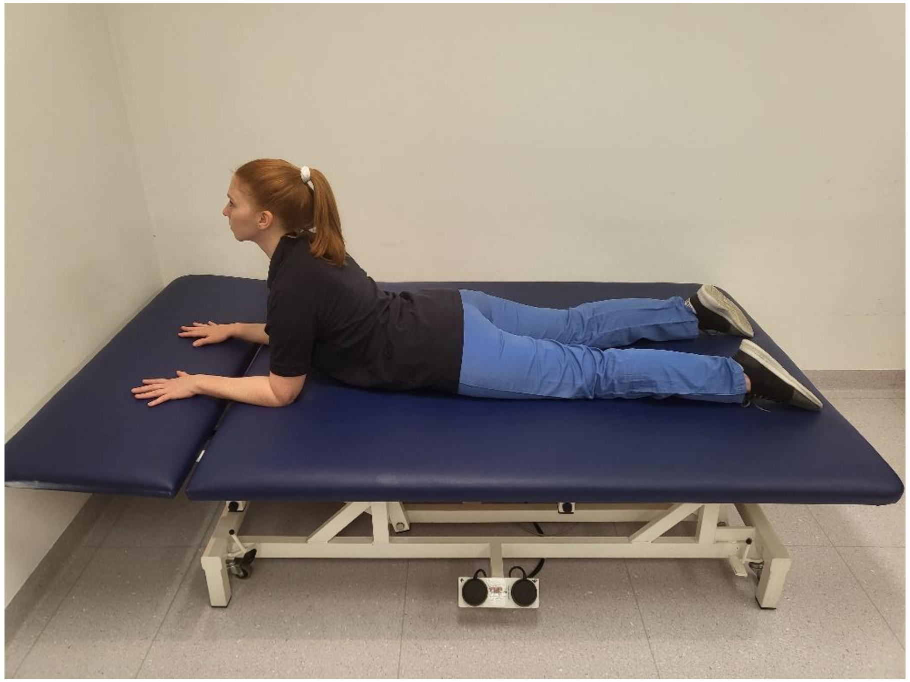

Stretching in prone position:

- Lie in the middle of the bed on your stomach
- Place your forehead or, if possible, chin on your hands
- Stretch out your toes and feet
- If possible, lean on your forearms and raise your head and upper body
- Hold this stretched position for few seconds, repeat 3 times

2. Movement & Posture (Level 1)

Lower body rotation while lying down

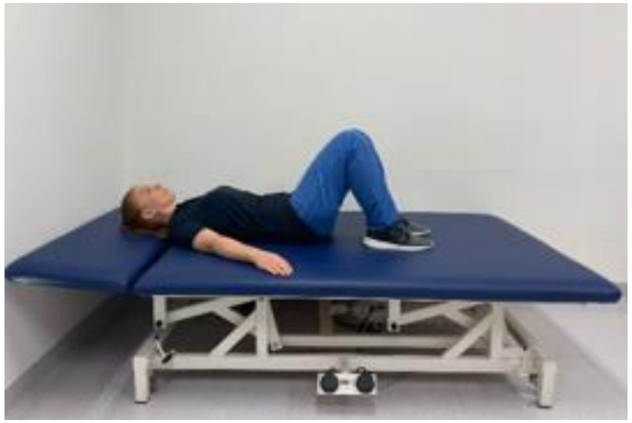
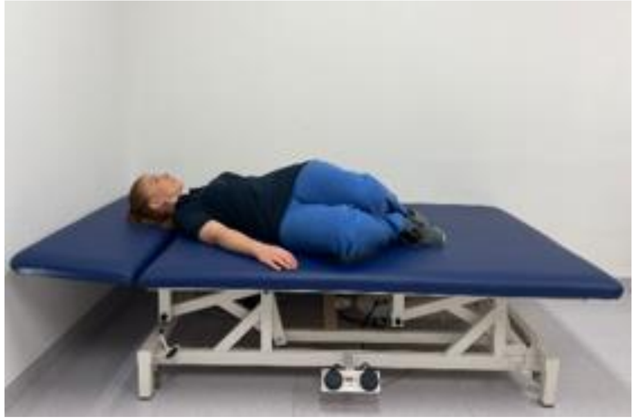
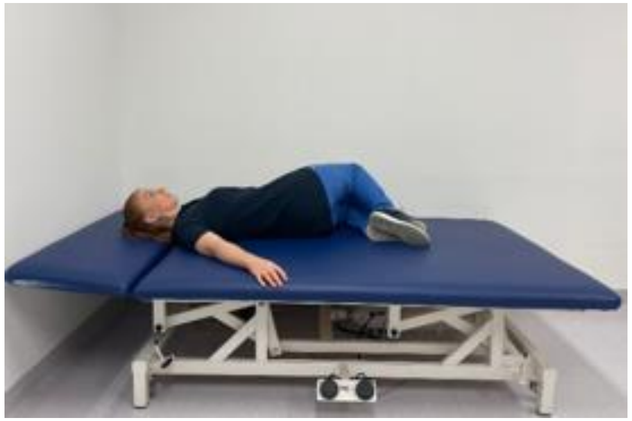

Rotation in the supine position:

- Lie in the middle of the bed on your back
- Pull up your legs and bend your knees, stretch your arms out to the side
- Turn both knees to the left and right at the same time without turning the upper part of your body
- Repeat at least 5-10 times

Tip: This exercise can reduce back pain.

2. Movement & Posture (Level 1)

Flexion and extension of the back

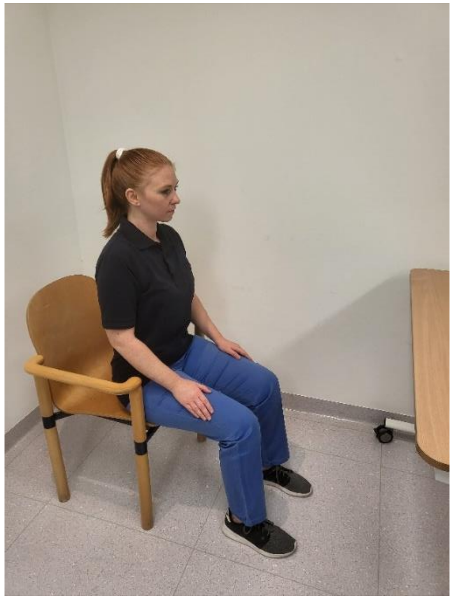
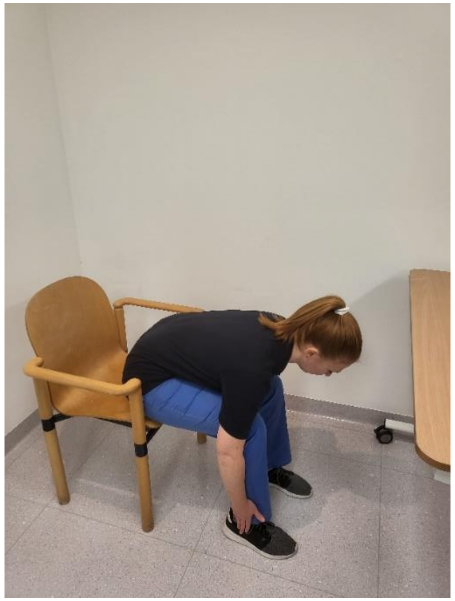
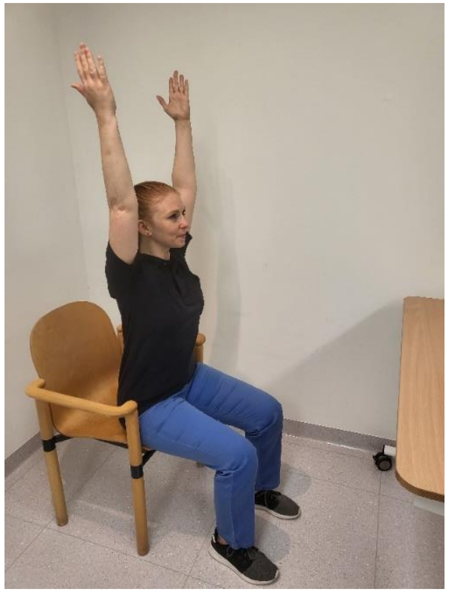

Flexion and extension of the back:

- Sit on the edge of a chair
- Bend down to the feet, touching the outer ankles on both sides (if not possible: touch the shins)
- Then slowly straighten your upper body and stretch your arms as high as possible towards the ceiling
- Repeat at least 5-10 times

Caution: this exercise can cause dizziness when bending down. If you feel dizzy or have blood pressure problems when changing your body position, please perform the exercise slowly and under supervision.

2. Movement & Posture (Level 1)

Large swings

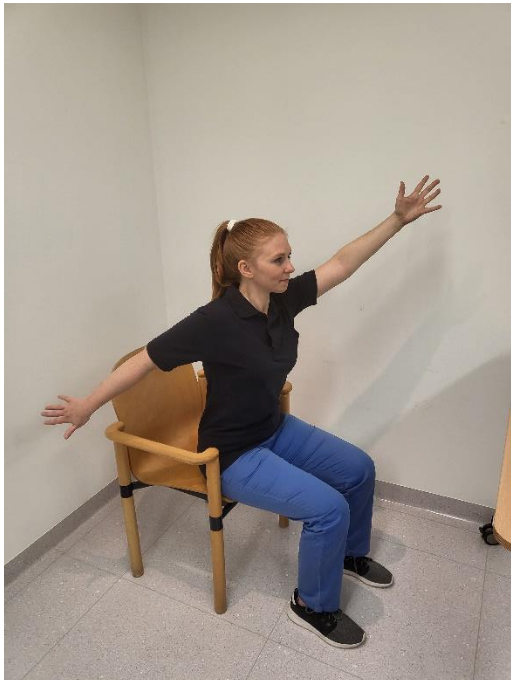

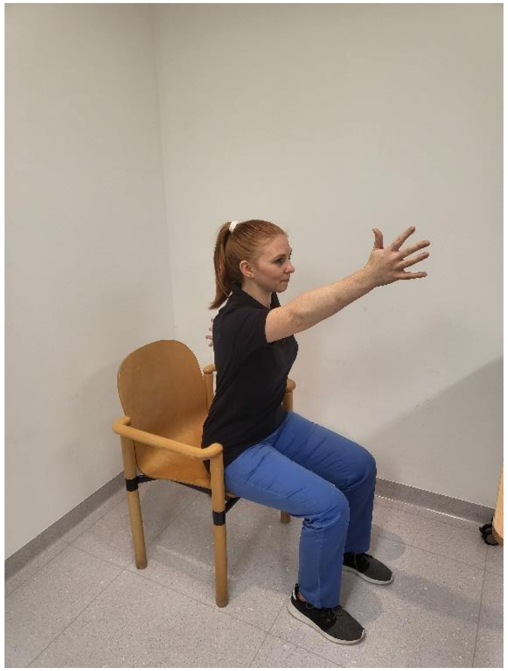

Large arm swing in the seated position:

- Sit on the edge of a chair
- Straighten your upper body
- Stretch your arms and perform large arm swings back and forth in opposite directions
- Repeat at least 5-10 times

Variation: Count out loud or listen to music and move following the rhythm.

2. Movement & Posture (Level 1)

Upper body rotation with lateral arm swing

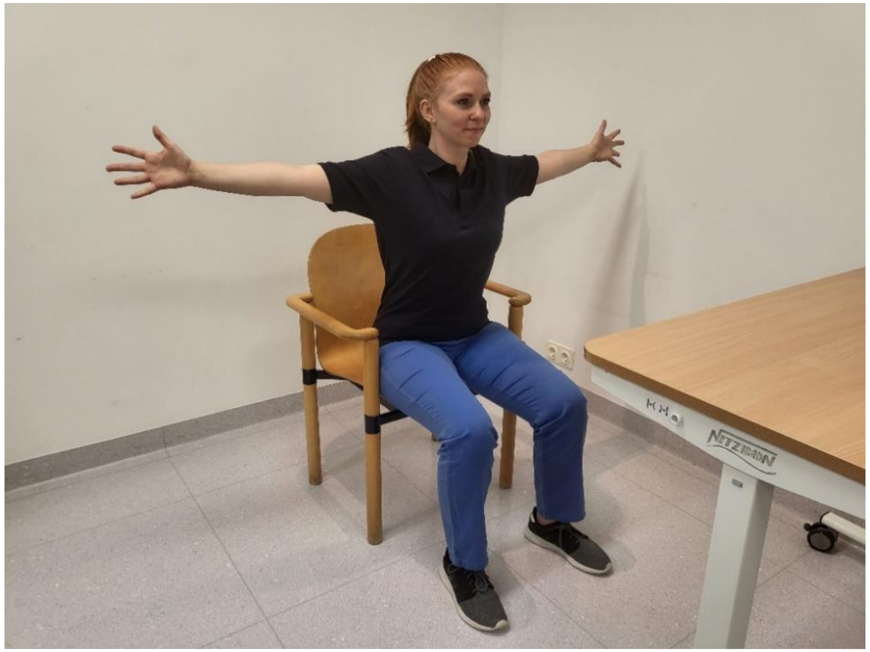

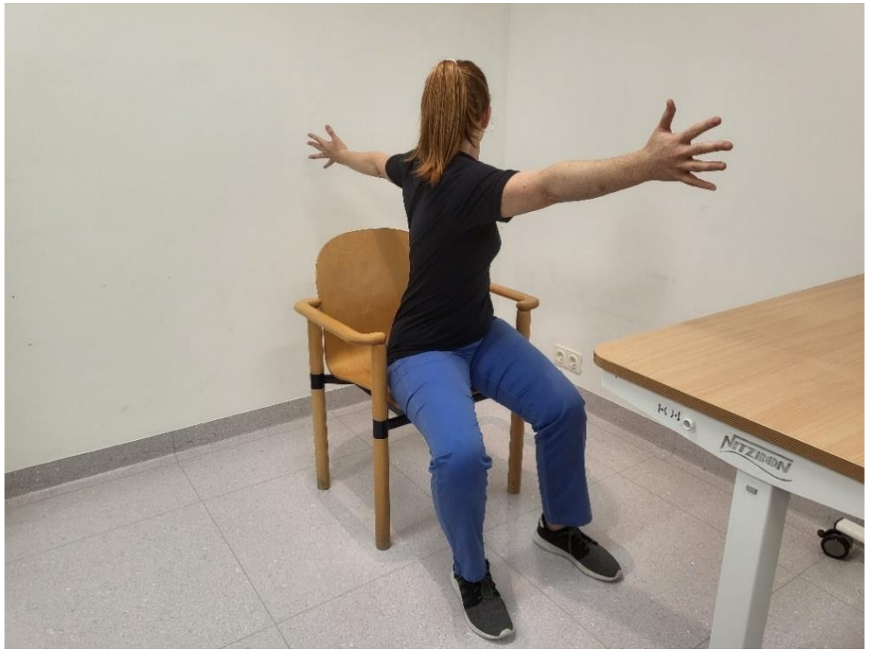

Upper body rotation in the seated position:

- Sit on the edge of a chair
- Straighten your upper body
- Stretch your arms out to the side and stretch your fingers wide apart
- Turn your upper body sideways and move your arms accordingly
- Repeat at least 5-10 times

Variation:

- Look at your left hand when turning to the left (see photo) and the same on the right:

Attention: Turning the head can cause dizziness. If you feel dizzy when turning your head, please perform the exercise slowly and under supervision.

2. Movement & Posture (Level 1)

Stretching the shoulder/neck muscles

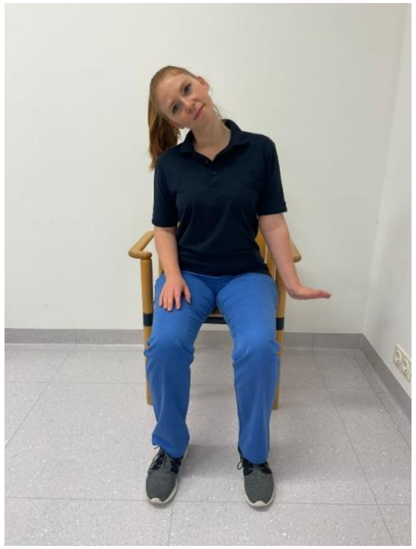 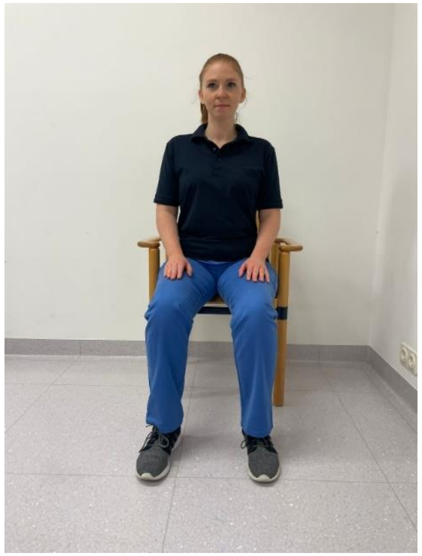

Stretching the shoulder and neck muscles:

- Sit on the edge of a chair
- Tilt your head to the right (right ear to the right shoulder; be careful not to turn your nose downwards)
- The left arm is stretched and pulls down towards the floor (this will increase the stretch)
- Hold this stretched position for few seconds
- Repeat the exercise to the other side, perform it altogether 3 times on each side

Tip: Perform this exercise in front of a mirror to prevent unintentional compensatory changes in posture.
This exercise can reduce shoulder and neck pain

2. Movement & Posture (Level 2)

Stretching chest muscles on the wall

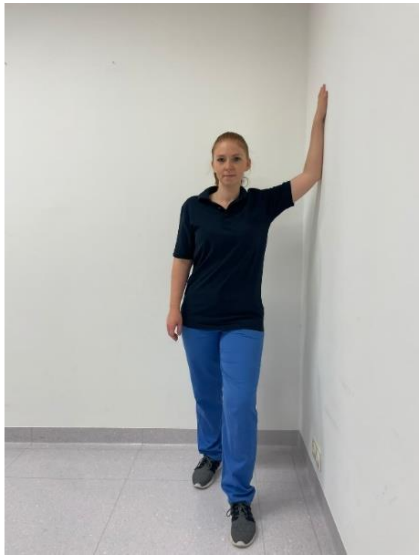

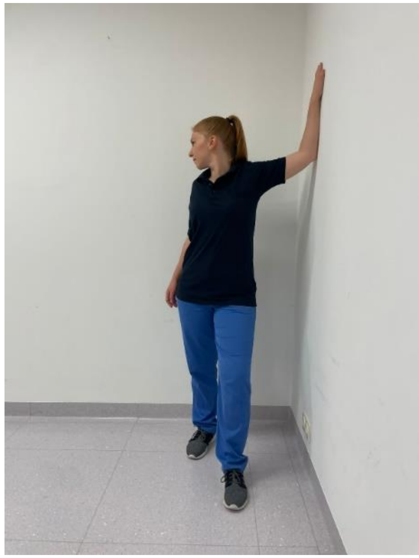

Stretching the chest muscles:

- Stand against the wall or door frame, adopt a wide stance with one foot forward, if necessary
- Brace your left forearm against the wall (elbow slightly above shoulder height)
- Turn your upper body far to the right and look to the right
- Hold this stretching position for few seconds
- Repeat the exercise to the other side, perform it altogether 3 times on each side

Attention: Safety first, please practice under the supervision of another person, if you feel unstable.

2. Movement & Posture (Level 2)

Stretching against the wall

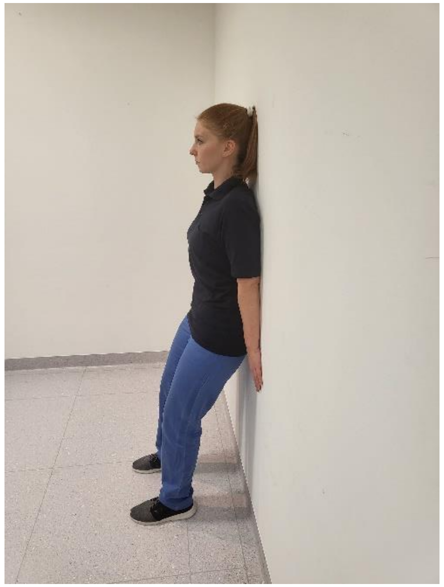

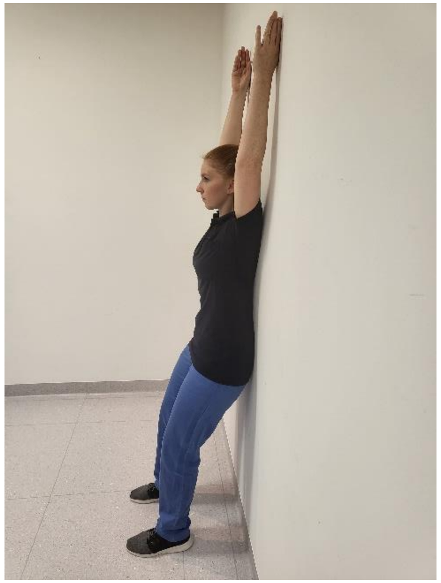

Stretching on the wall:

- Wide-legged stance one foot away from the wall
- Lean against the wall with your buttocks, back and head
- Try to raise your arms over your head. The arms should also lean against the wall to avoid forming a hollow back
- Hold this stretched position for few seconds, repeat 3 times

Variation: Lean your arms stretched to the side against the wall.

Attention: Safety first, please practice under the supervision of another person, if you feel unstable.

2. Movement & Posture (Level 2)

Back extension from prone position

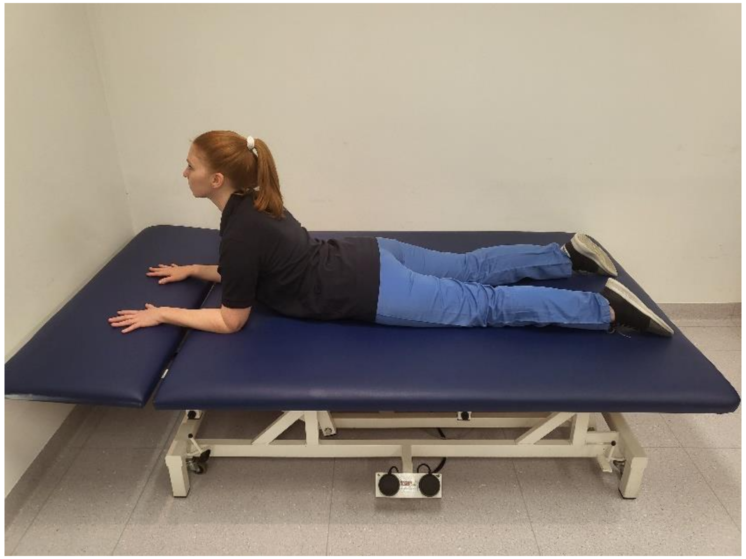

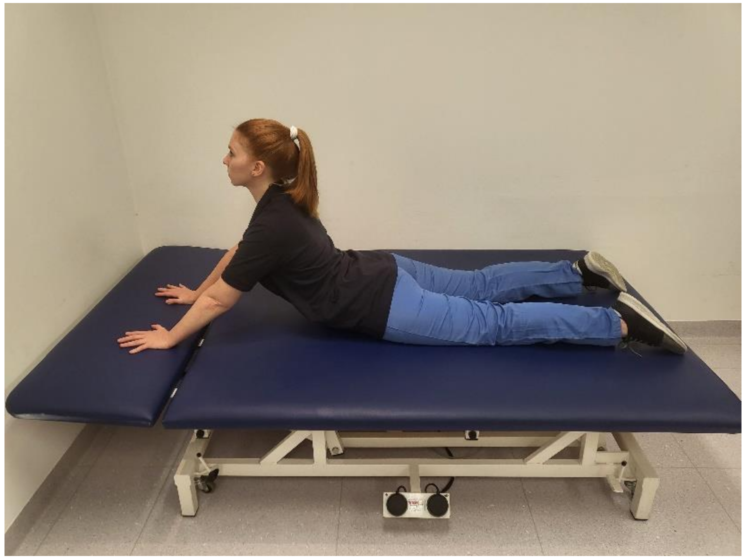

Stretching in prone position:

- Lie in the middle of the bed on your stomach and stretch out your toes and feet
- Lean on your forearms and raise your head and chest
- Lean on your hands, straighten your elbows and raise your upper body and head
- Hold this position for few seconds, repeat 3 times

3. Circulation (Level 1)

Foot tapping

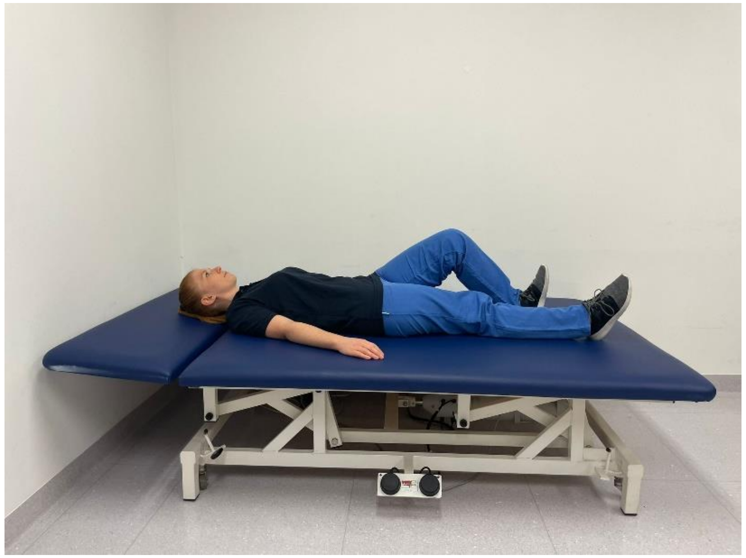

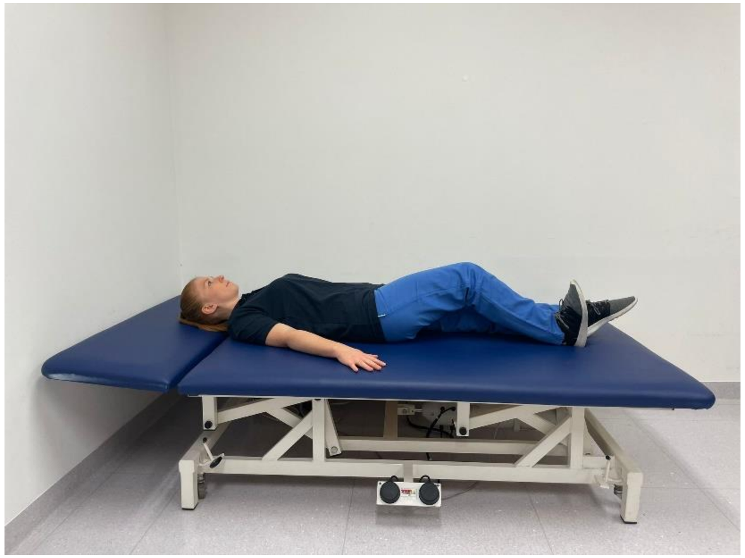

Foot tapping in the supine position:

- Lie in the middle of the bed on your back and stretch out your legs
- Alternately pull your foot and knee towards your nose and then stretch them out again
- Repeat this movement 10-20 times

3. Circulation (Level 1)

Pull up and stretch legs

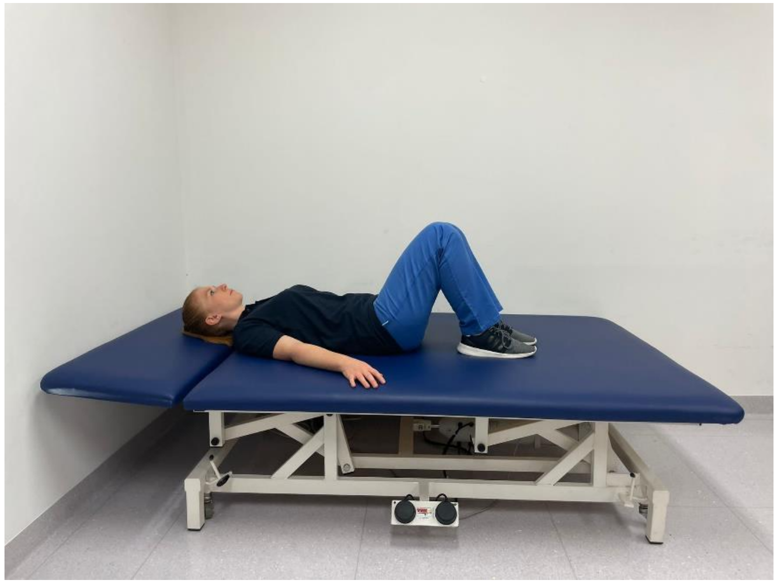

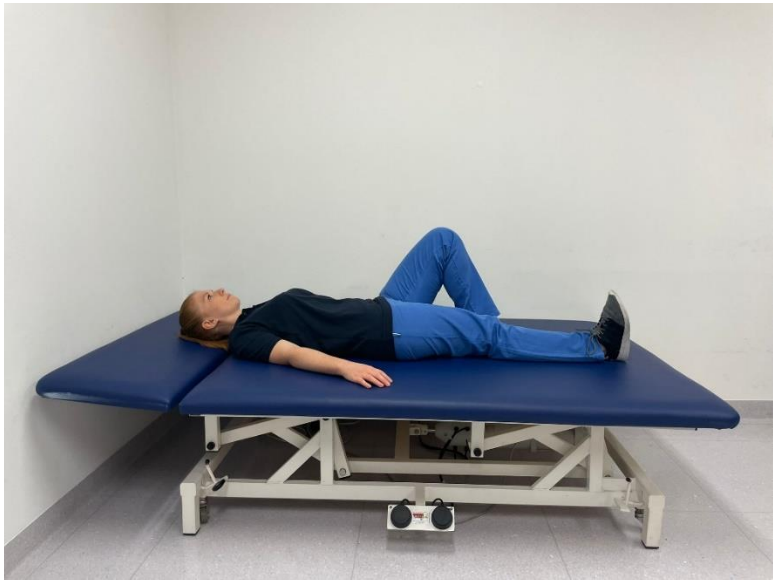

Pull your legs up and bend your knees while lying down:

- Lie in the middle of the bed on your back and pull both legs up, with knees bent
- Alternately stretch out one leg and put it back in place
- Repeat this movement 10-20 times

3. Circulation (Level 1)

Single leg cycling

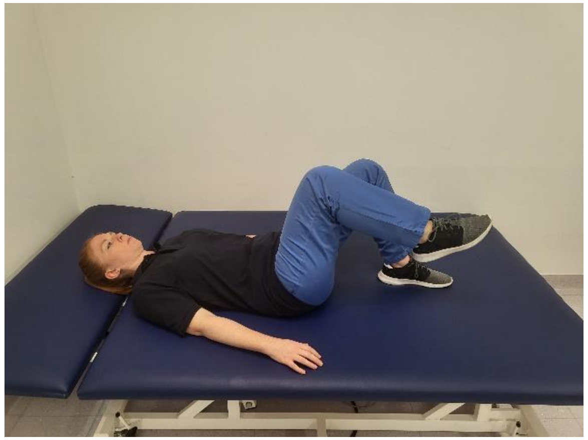
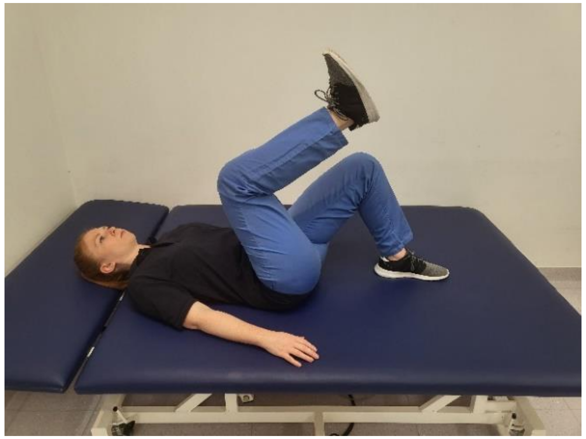
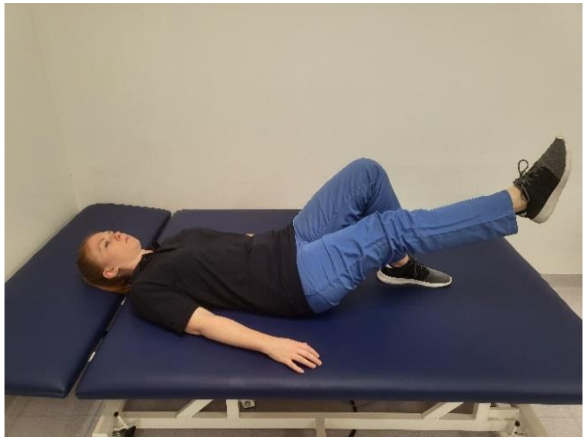

Cycling on one leg while lying down:

- Lie in the middle of the bed on your back and pull both legs up with your knees bent
- Raise one leg in the air and perform cycling movements
- Repeat this movement 10-20 times, then change the leg
- Repeat altogether 3 times per side

Tip: The wider the leg movement is, the more difficult the exercise is. Lower abdominal muscles are also trained.

3. Circulation (Level 1)

Bend & stretch wrists

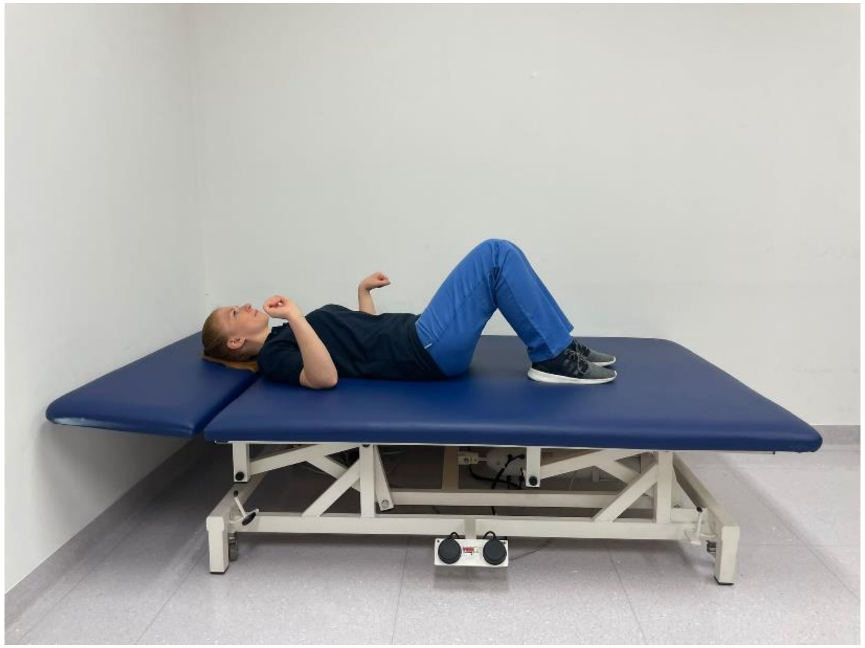

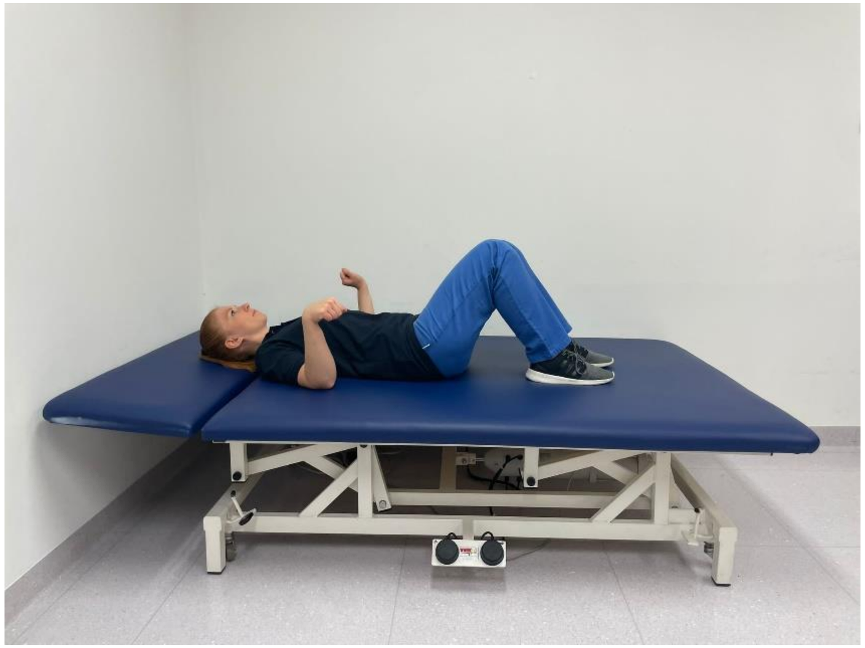

Move wrists while lying down:

- Lie in the middle of the bed on your back and pull both legs up with knees bent.
- Bend your elbows so that your hands are in the air
- Alternately bend one wrist and stretch the other
- Repeat this movement 10-20 times

3. Circulation (Level 1)

Stretch your arms to the ceiling

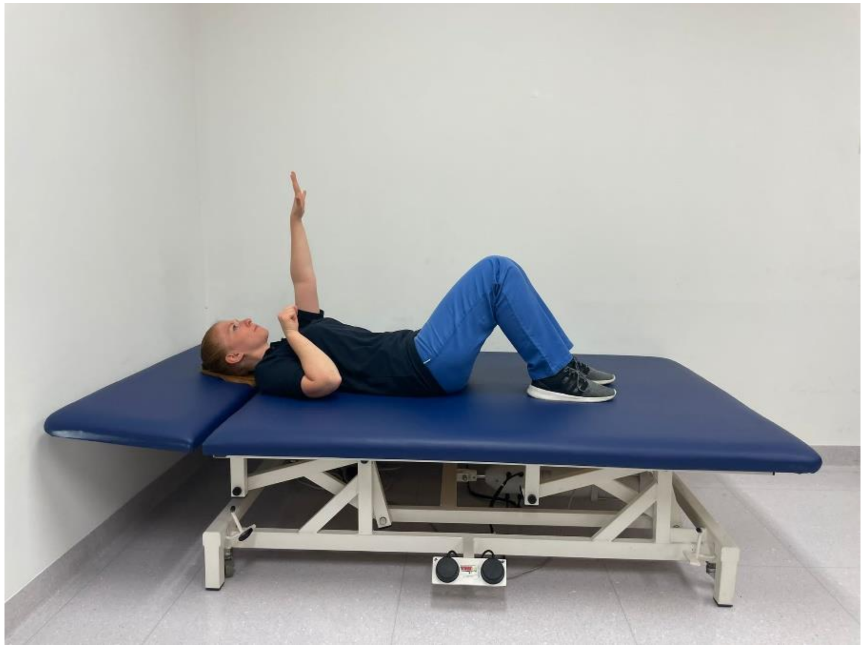

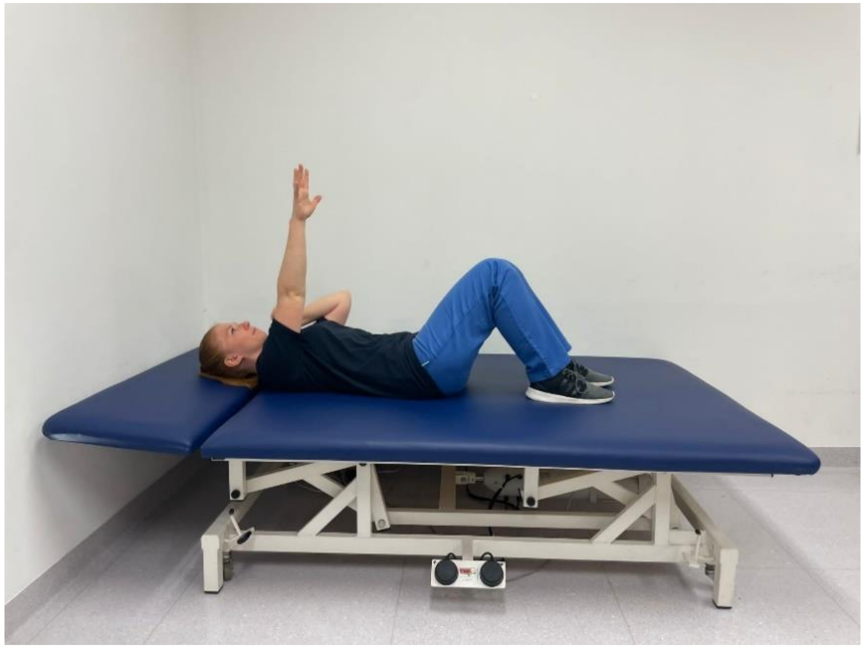

Stretch your arms towards the ceiling:

- Lie in the middle of the bed on your back and pull both legs up with knees bent
- Alternately stretch arms as far as possible towards the ceiling
- Stretch your fingers out wide and spread them apart, forming a fist as you bring them down (like picking apples from a tree)
- Repeat this movement 10-20 times

3. Circulation (Level 2)

Arm swings in seated position

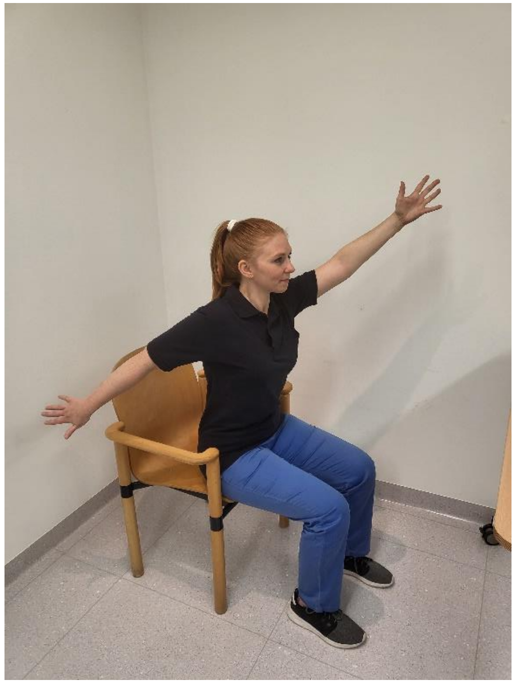

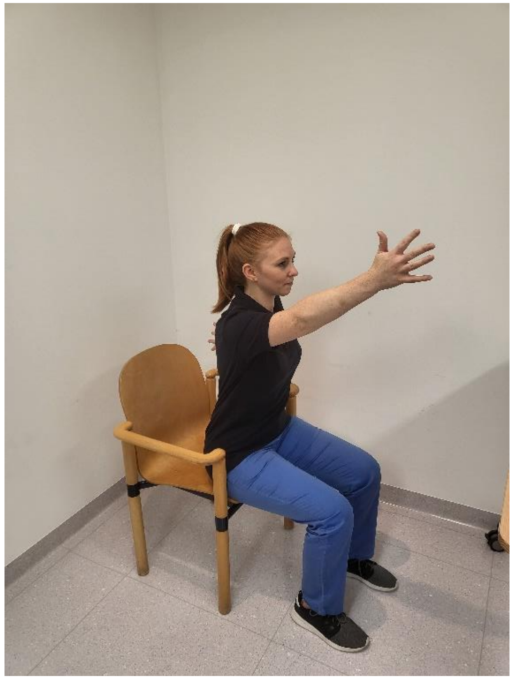

Arm swings while seated:

- Sit on the edge of a chair
- Straighten your upper body
- Stretch your arms and perform large arm swings back and forth in opposite directions
- Repeat this movement 10-20 times

3. Circulation (Level 2)

Boxing in the air

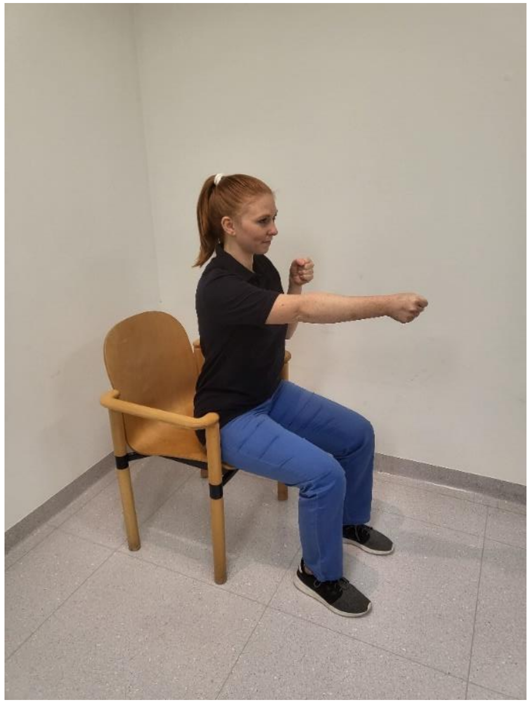

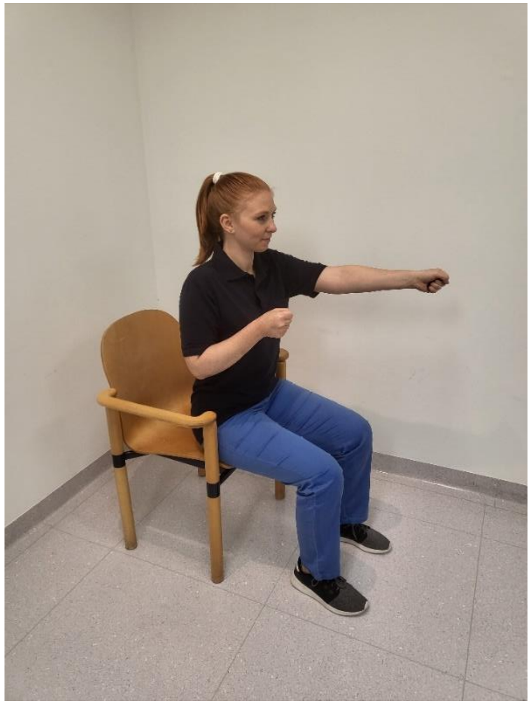

Boxing in the air while seated:

- Sit on the edge of a chair
- Straighten your upper body
- Form fists and alternately punch forward with large, quick movements
- Repeat this movement 10-20 times

3. Circulation (Level 2)

Stand up and sit down

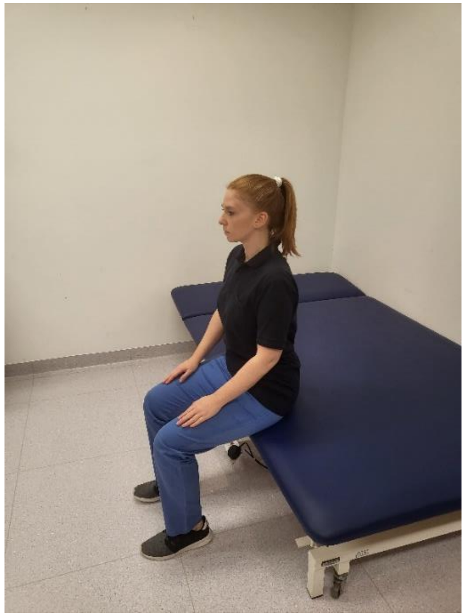

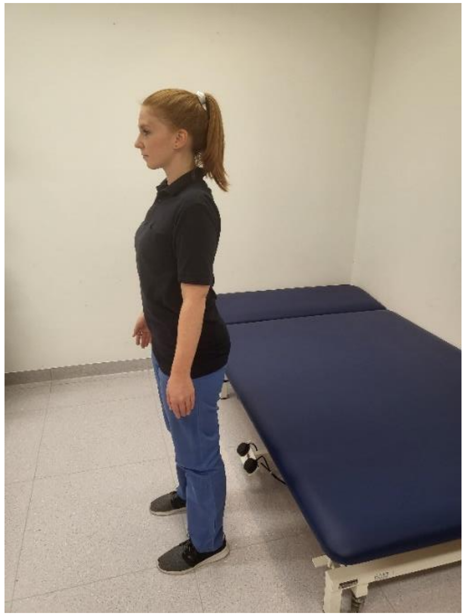

Stand up and sit down:

- Sit on the edge of the bed or chair, if necessary, place your hands on your thighs
- Place your feet wide apart and place your heels far back, as close as possible to the bed or chair
- Bring the upper body forward with momentum, lift the buttocks and straighten the body
- If necessary, try several times until the upper body is far enough forward
- Repeat at least 10 times

Tip: If you feel dizzy or have blood pressure problems, please perform the exercise slowly and remain standing for a while, then sit down again, if necessary.

Attention: Safety first, please practice under the supervision of another person, if feeling unstable.

4. Balance (Level 1)

Sit without support

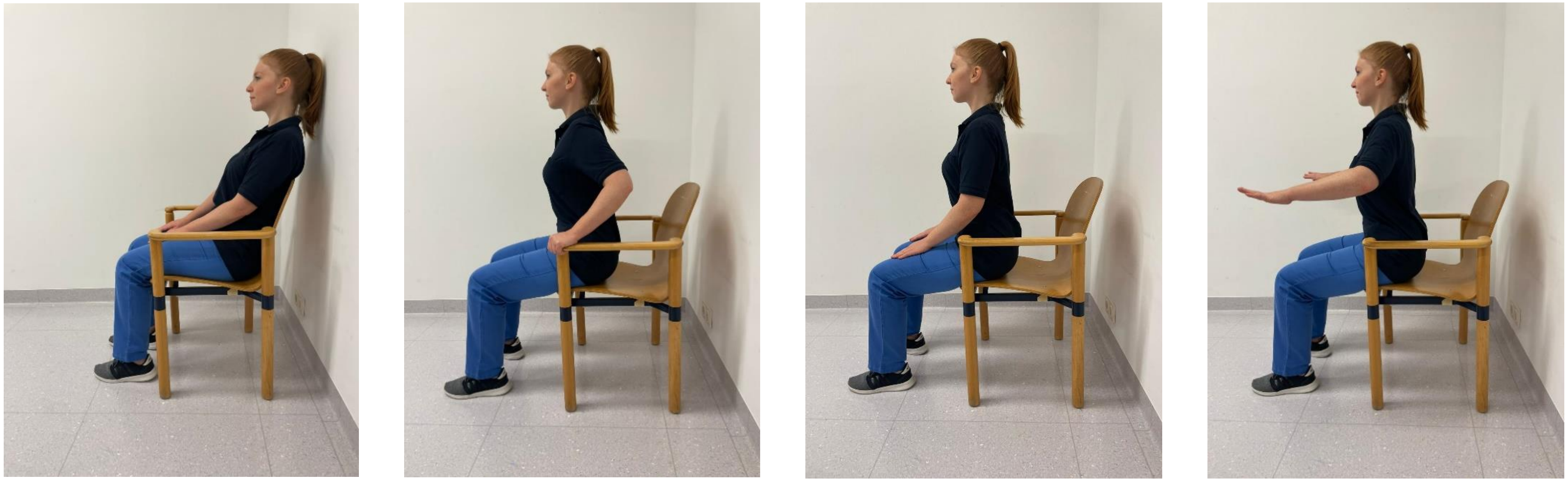

Sit without support:

- Sit on the edge of the bed or chair
- Place your feet wide apart and straighten your upper body
- Hold on to the armrests with your hands or place your hands on your thighs
- Raise both hands in the air and try to keep your upper body upright
- Hold this position for few seconds, repeat 3 times

4. Balance (Level 1)

Sit without support while moving the arms

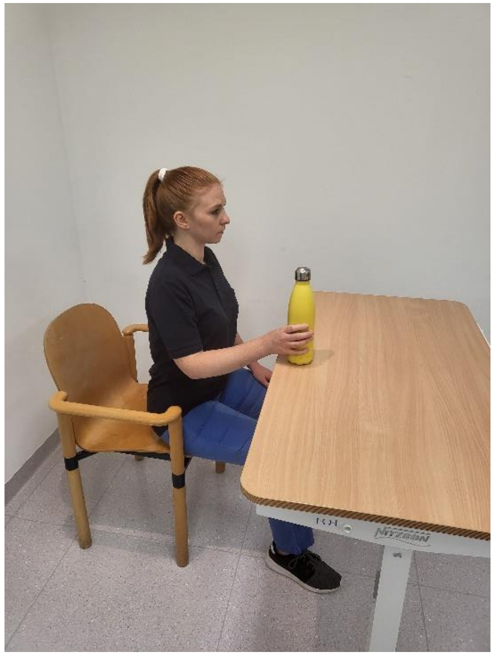
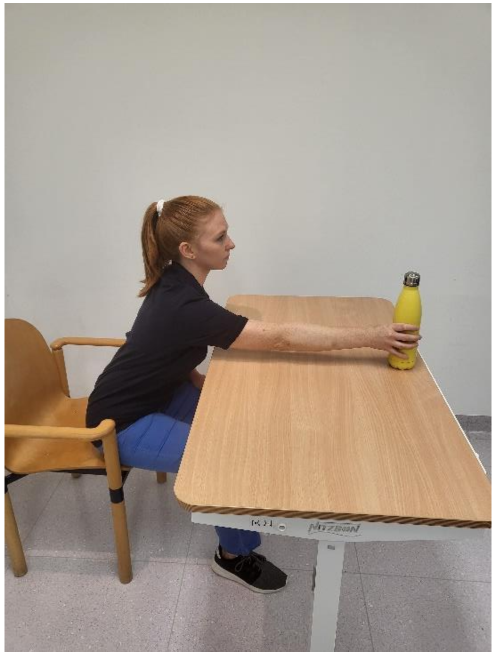
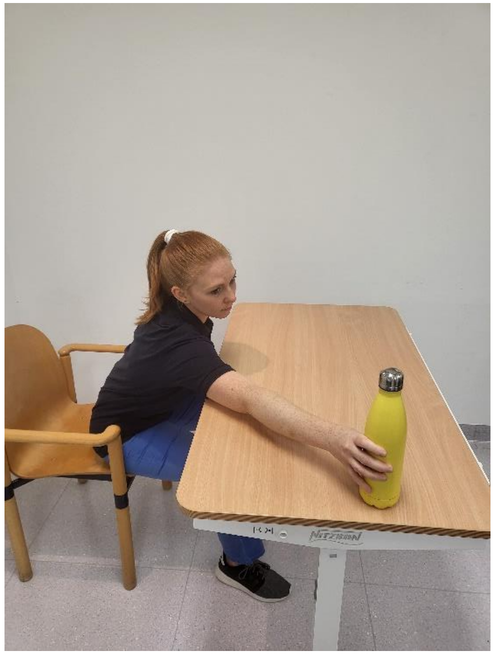

Sit without support while moving the arms:

- Sit on the edge of the bed or chair in front of a table (or similar)
- Feet are wide apart and the upper part of the body is straightened
- Grab an object and move it back and forth / left and right / diagonally as far as possible on the table
- Repeat this movement 10-20 times, then change arm. Repeat 3 times per side

Variation: Increase the weight of the object to increase difficulty (e.g., filled water bottle).

4. Balance (Level 1)

Standing with support

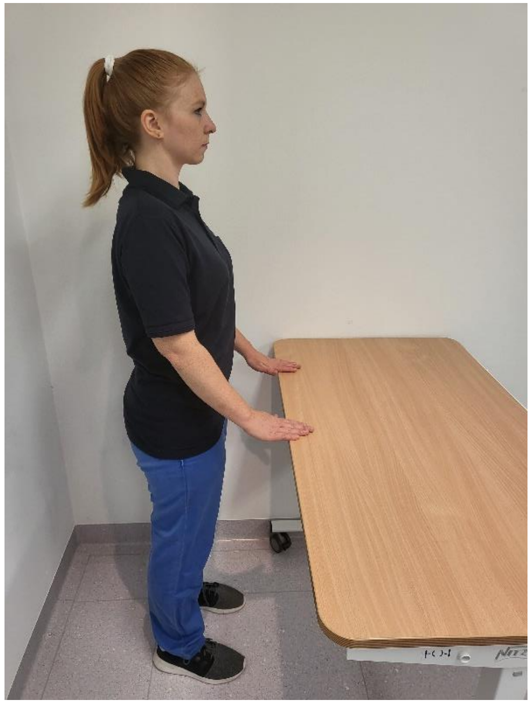

Standing with support:

- Stand with the feet wide apart and hold onto a table (alternatively windowsill, handrail, railing, kitchen counter, sink)
- Hold on with both hands and straighten your upper body

Variation: The exercise becomes more difficult if looking upwards/downwards and left/right or by adopting a narrower stance.

Attention: Safety first, please practice under the supervision of another person, if feeling unstable.

4. Balance (Level 2)

Standing without support

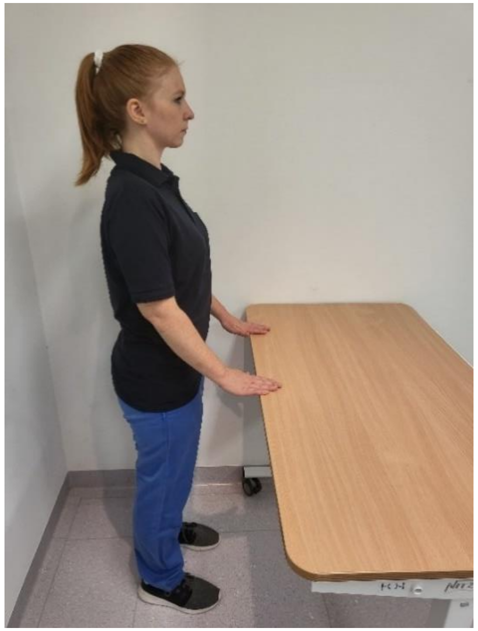

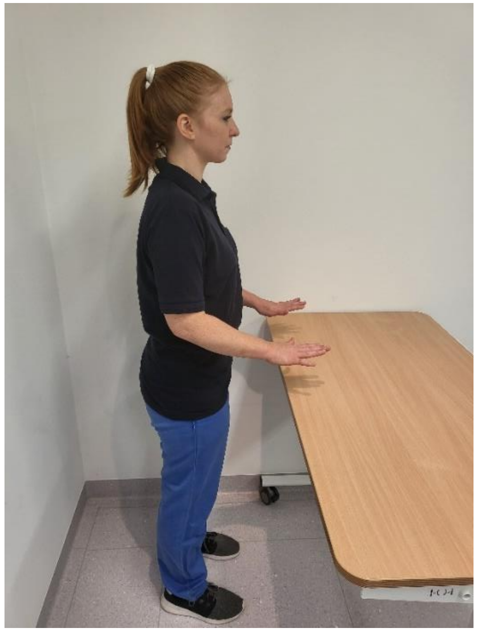

Standing without support:

- Stand with the feet wide apart in front of a table (alternatively window sill, handrail, railing, kitchen counter, sink)
- Hold on with both hands and straighten your upper body
- Lift both hands off the table for a few seconds and use them to remain balanced

Attention: Safety first, please practice under the supervision of another person, if feeling unstable.

4. Balance (Level 2)

Standing without support while moving your arms

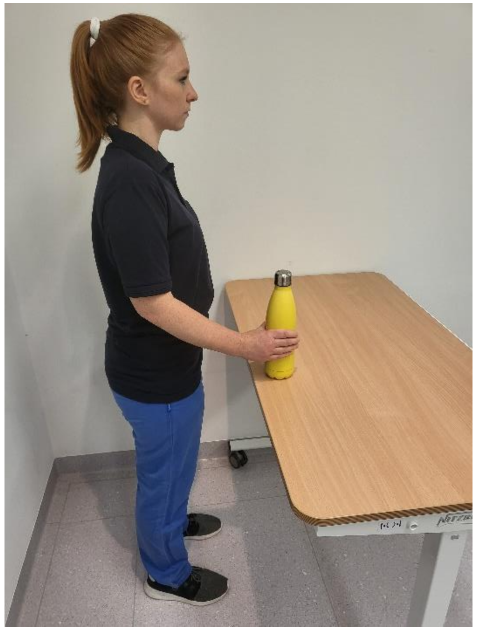

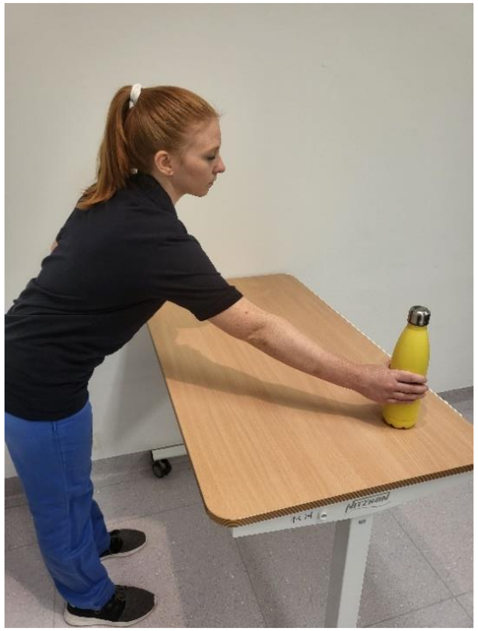

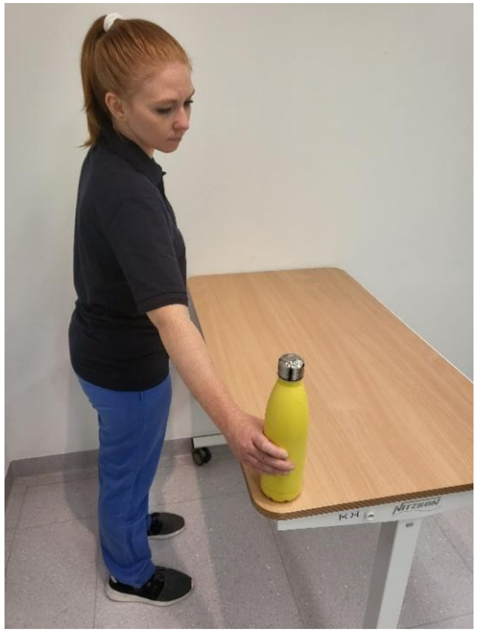

Standing without support while moving your arms:

- Stand with your feet wide apart in front of a table (or another supporting surface)
- Straighten your upper body
- Grab an object and move it back and forth / left and right / diagonally as far as possible on the table
- Repeat this movement 10-20 times, then change arm
- Repeat the exercise 3 times per side

Variation: Increase the weight of the object to increase difficulty (e.g., filled water bottle).

Attention: Safety first, please practice under the supervision of another person, if feeling unstable.

4. Balance (Level 2)

Stand up while lifting an object from the floor

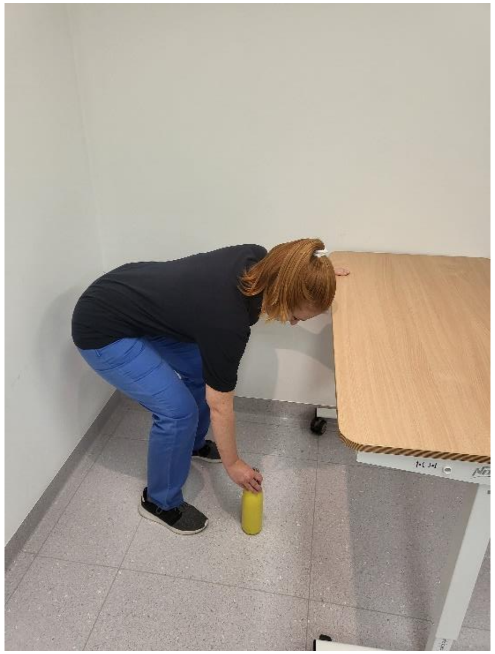

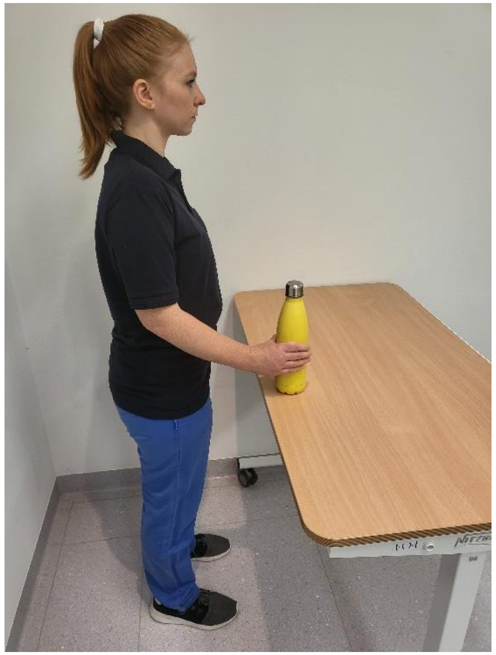

Stand up while lifting an object from the floor:

- Stand with your feet wide apart in front of a table (or another supporting surface)
- Straighten the upper body, if necessary, hold on to the table with one hand
- Grab an object and place it alternately on the floor and back on the table
- Repeat this movement 5-10x, 3x per side

Variation: Increase the weight of the object to increase difficulty (e.g., filled water bottle).

Attention: Safety first, please practice under the supervision of another person, if feeling unstable.

4. Balance (Level 2)

Steps forwards/sideways/backwards with support

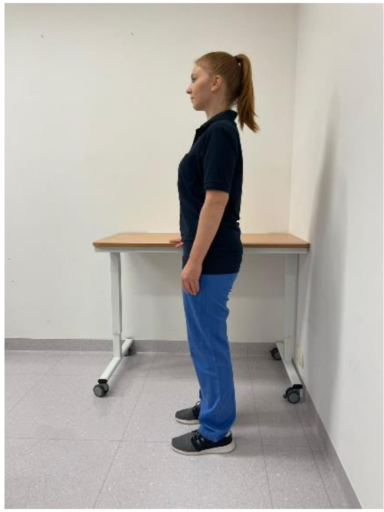
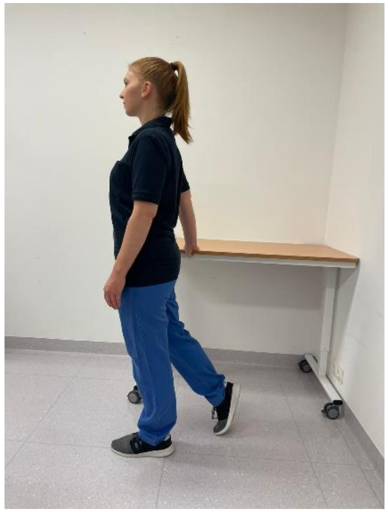
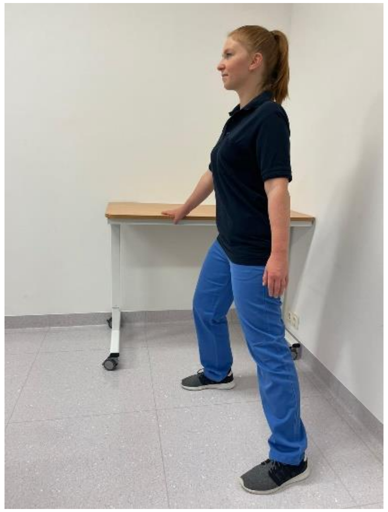
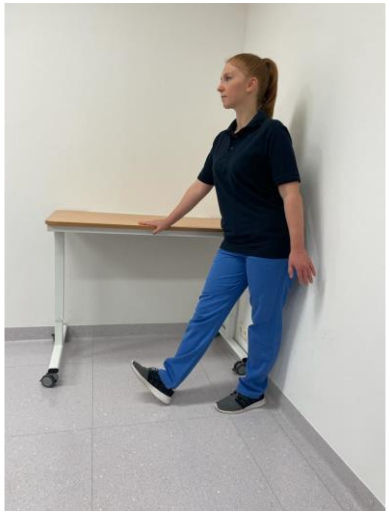

Stepping in different directions with support:

- Stand with your feet wide apart in front of a table (or another supporting surface)
- Hold on with one hand and straighten your upper body
- Alternately take a step forward, then sideways and then backwards, always putting your foot back to the initial position in between
- When walking forward, the heel should touch the ground first, when walking sideways the whole foot should touch the ground first and when walking backwards the toes should touch the ground first
- It is important not just to tap the foot on the floor, but to move most of the body weight to the stepping leg
- Repeat the movement sequence of step forward-step backward-step sideways-step backward-step backward 5-10 times per side
- Repeat the whole sequence 3 times per side

Variation: Instead of performing all movements one after the other, it is also possible to practice them separately (e.g., 10x step forward).

Attention: Safety first, please practice under the supervision of another person, if feeling unstable.

5. Strength (Level 1)

Back muscles

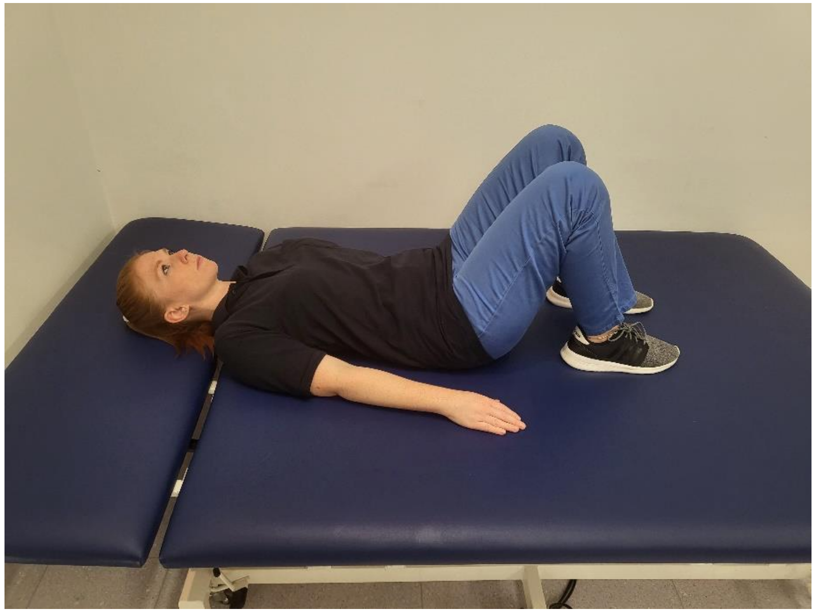 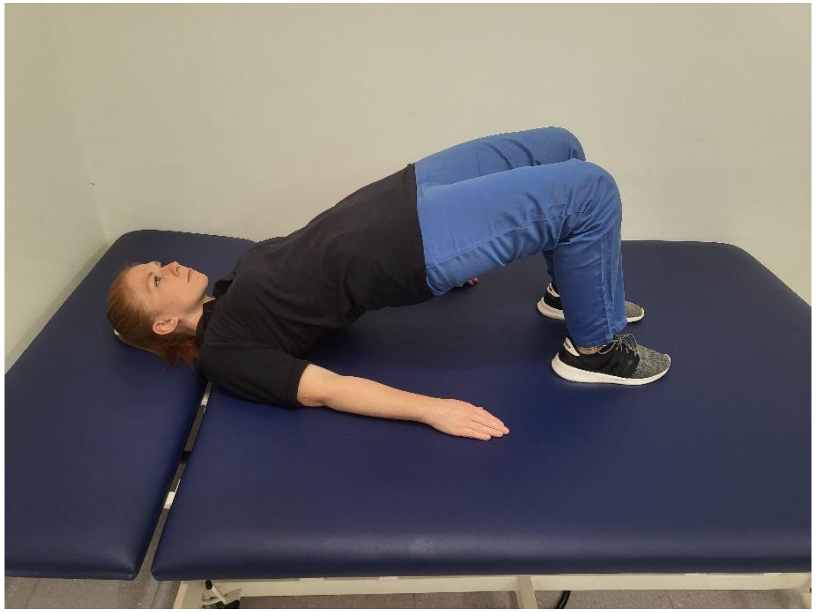

Back strength training:

- Lie flat on your back (use only a thin or no pillow), place your arms loosely next to your body and pull both legs up with your knees bent
- Lift your buttocks as high as possible from the surface and stretch your back
- Slowly lay down again and repeat
- Repeat the exercise 5 times, then take a break (feel free to move your knees to the left and right or stretch your legs)
- Repeat the sequence 3 times altogether

Caution: If the muscles are weak, the posterior thigh may quickly develop cramps; pressure may be felt in the lumbar spine. Taking breaks in between exercises helps to prevent or ease discomfort.

5. Strength (Level 1)

Abdominal muscles

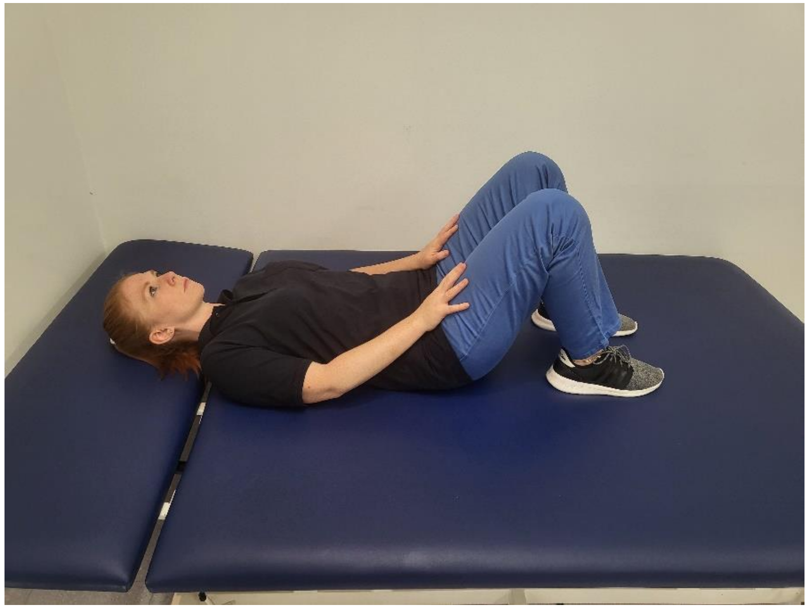

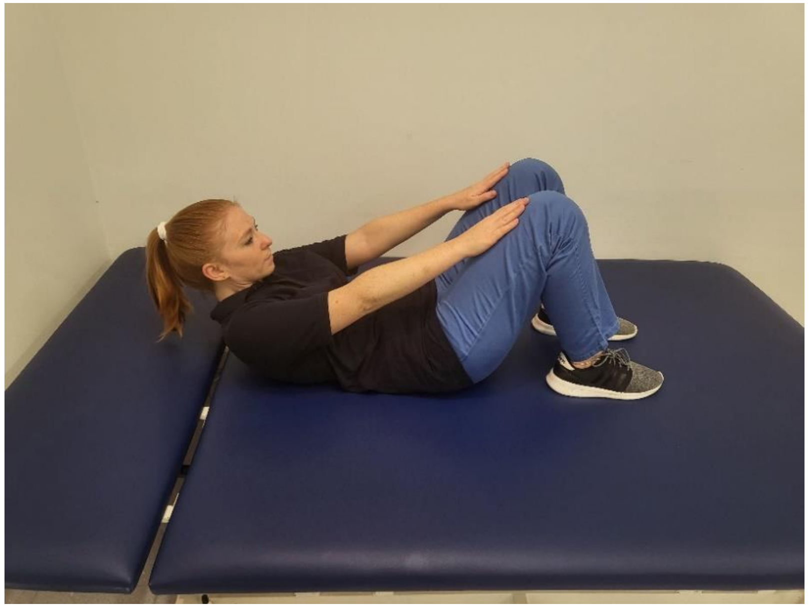

Abdominal strength training:

- Lie flat on your back, (use only a thin or no pillow)
- Place your arms loosely next to your body and pull both legs up with your knees bent
- Lift your head and push it up towards your knees with your hands, lifting your shoulders from the support
- Slowly lay down again and repeat
- Repeat the exercise 5 times, then take a break (stretch your legs, if you'd like)
- Repeat the sequence 3 times altogether

Caution: If muscles are weak, this exercise may cause tension in the neck or stomach area. Taking breaks in between exercises helps to prevent or ease discomfort.

5. Strength (Level 1)

Leg muscles

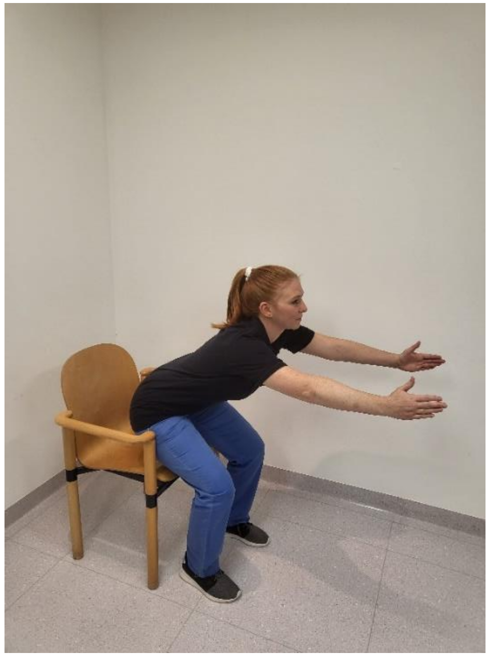

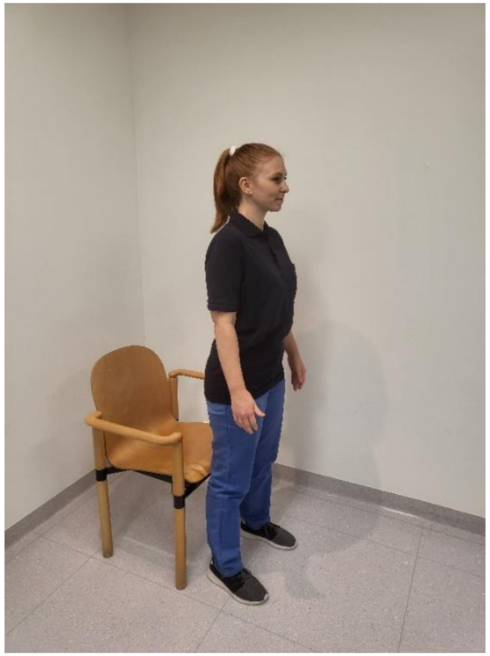

Leg strength training:

- Slide to the edge of the chair, the feet are wide apart and place your heels far back under the chair
- Stretch your hands forward and stand up with momentum, if necessary, try several times until your upper body is far enough forward
- Repeat the exercise 5 times, then take a short break in the seated position
- Repeat the sequence 3 times altogether

Variation: The exercise is easier, if the hands support onto the thighs.

Attention: Safety first, please practice under the supervision of another person, if feeling unstable.

5. Strength (Level 1)

Arm muscles

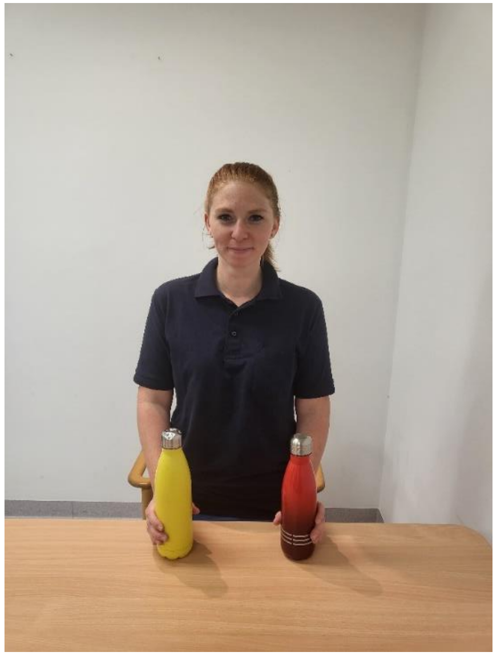

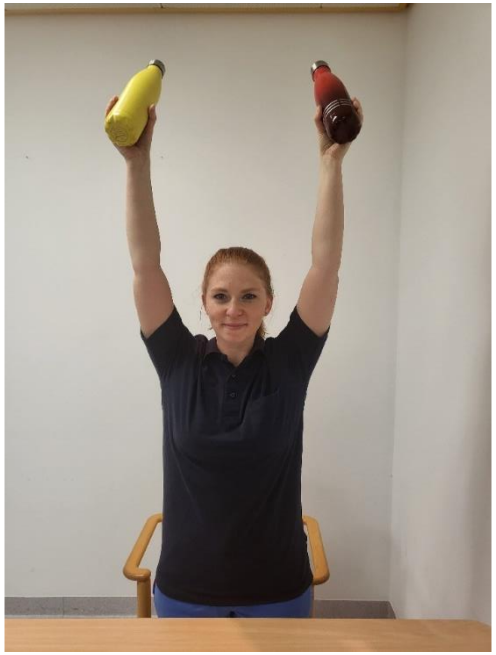

Arm strength training:

- Stand with feet wide apart and upper body straightened in front of a table (alternatively perform the exercise while sitting)
- Contemporarily lift two identical objects (e.g., bottles) towards the ceiling and place them back on the table
- Repeat the exercise 5 times, then take a short break
- Repeat the sequence 3 times altogether

Variation: Increase the weight of the object to increase difficulty (e.g., filled water bottle).

5. Strength (Level 2)

Back muscles

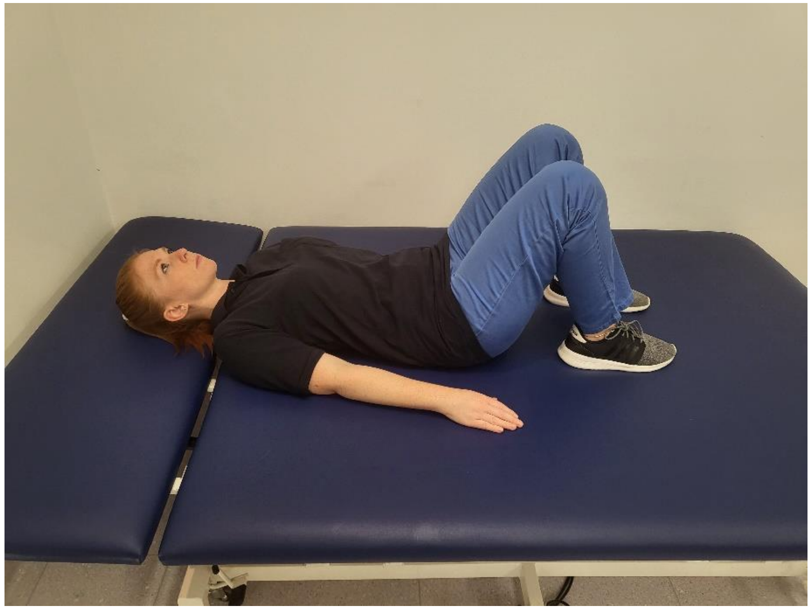

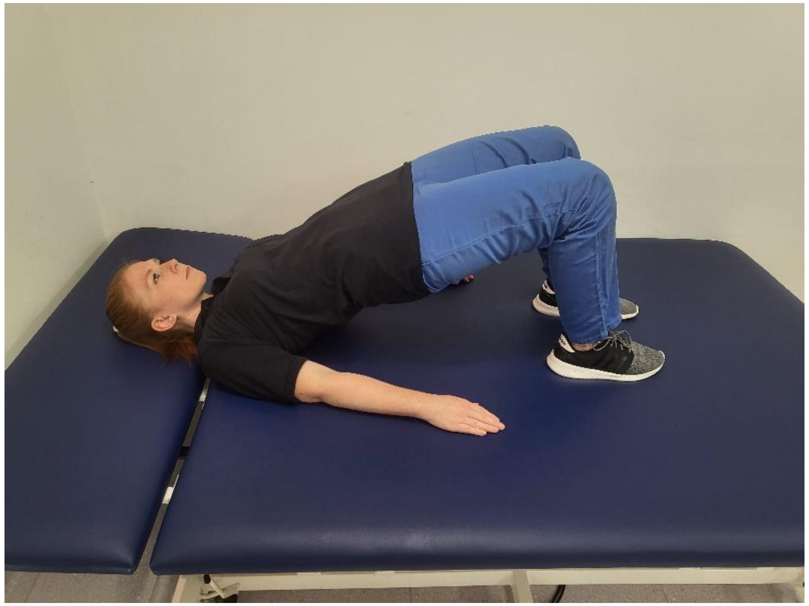

Back strength training:

- Lie flat on your back (use only a thin or no pillow), place your arms loosely next to your body and pull both legs up with your knees bent
- Lift your buttocks as high as possible from the surface and stretch your back
- Slowly lay down again and repeat
- Repeat the exercise 15 times, then take a break (during the break, feel free to stretch your legs or move them sideward). Repeat the sequence 3 times.

Caution: If muscles are weak, the posterior thigh can cramp quickly; pressure may be felt in the lumbar spine. Taking breaks in between exercises helps to prevent or ease discomfort.

5. Strength (Level 2)

Abdominal muscles

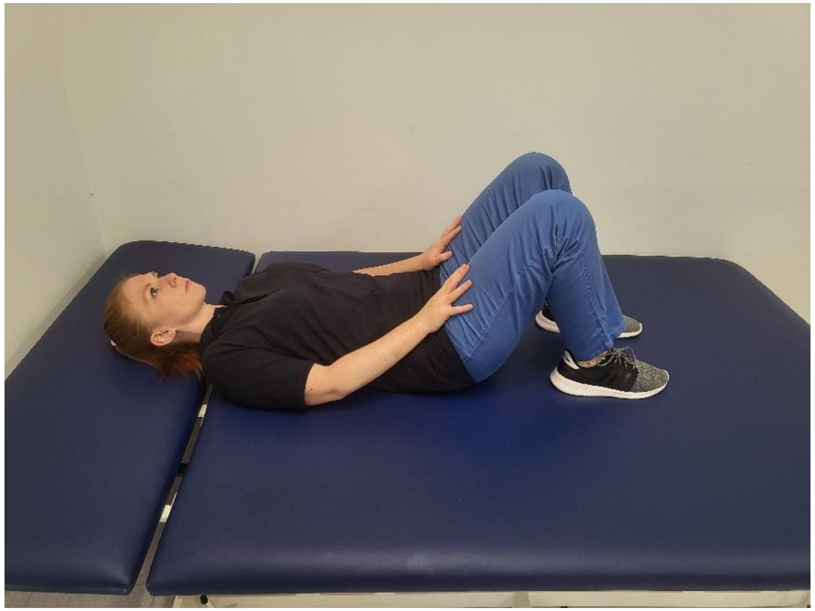

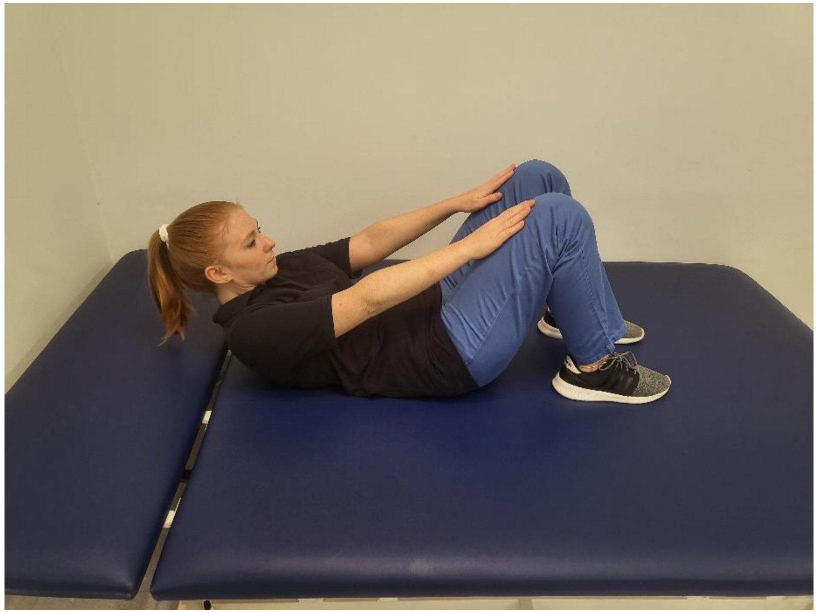

Abdominal strength training:

- Lie flat on your back (use only a thin or no pillow)
- Place your arms loosely next to your body and pull both legs up with your knees bent
- Lift your head and push it up towards your knees with your hands, lifting your shoulders from the support
- Slowly lay down again and repeat
- 3x 15 repetitions, take a break after 15 repetitions (feel free to stretch your legs during the break)

Caution: This exercise can cause tension in the neck and stomach area if the muscles are weak. Taking breaks in between exercises helps to prevent or ease discomfort.

5. Strength (Level 2)

Leg muscles

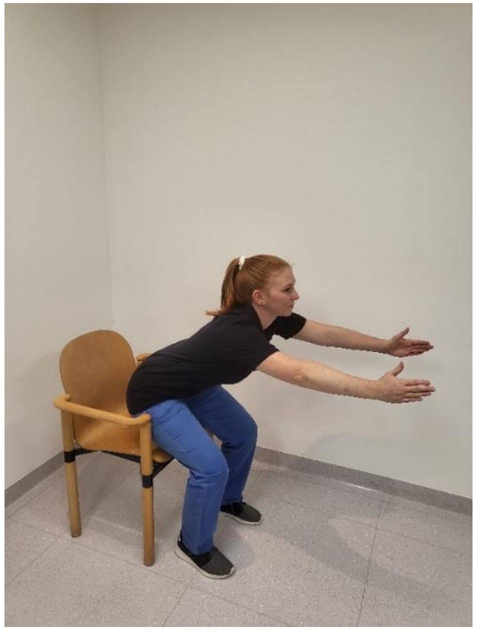

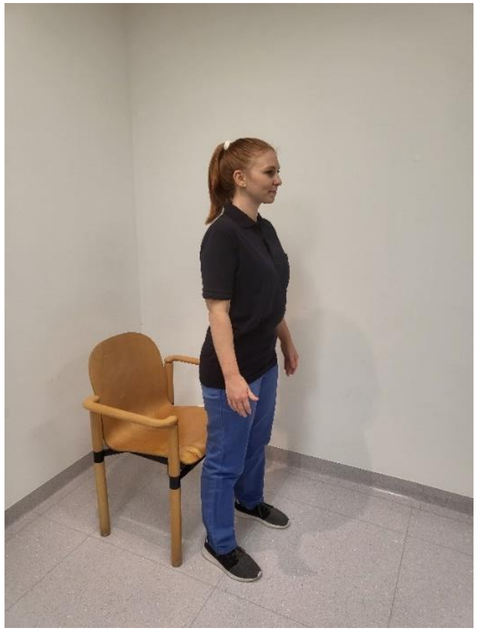

Strength training legs:

- Slide to the edge of the chair, the feet are wide apart
- Place your heels far back under the chair
- Stretch your hands forward and stand up with momentum, if necessary, try several times until your upper body is far enough forward
- Repeat the exercise 15 times, then take a short break in the seated position
- Repeat the sequence three times altogether

Variation: The exercise turns more difficult, if you do not sit down (i.e., perform squats instead).

Attention: Safety first, please practice under the supervision of another person, if feeling unstable.

5. Strength (Level 2)

Arm muscles

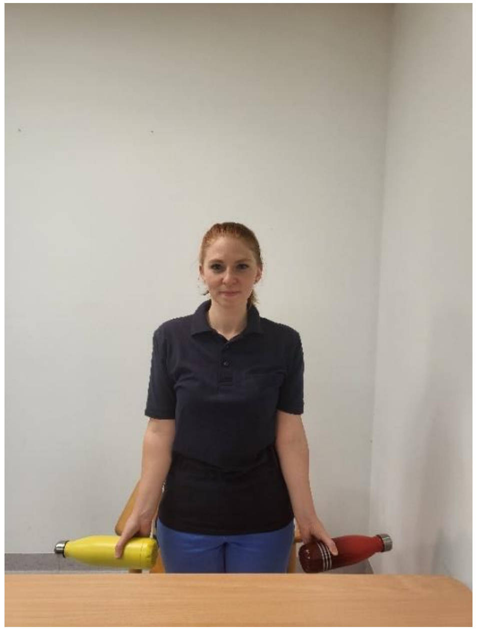
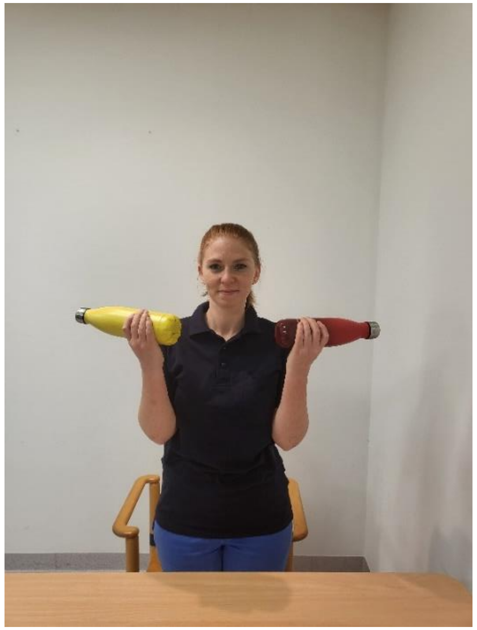
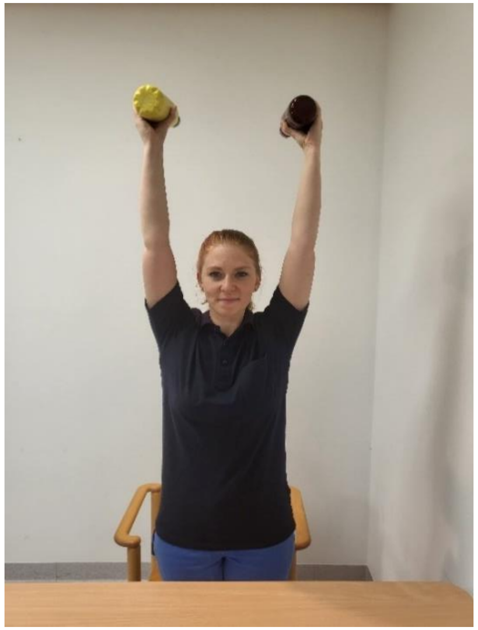

Strength training arms:

- Stand with feet wide apart and upper body straightened in front of a table (alternatively perform the exercise while sitting)
- Contemporarily lift two identical objects (e.g., bottles) towards the ceiling and place them back on the table
- Repeat the exercise 15 times, then take a short break. Repeat the sequence three times altogether

Variation: Increase the weight of the object to increase difficulty (e.g., filled water bottle).

6. Gait (Level 1)

Walking with assistance

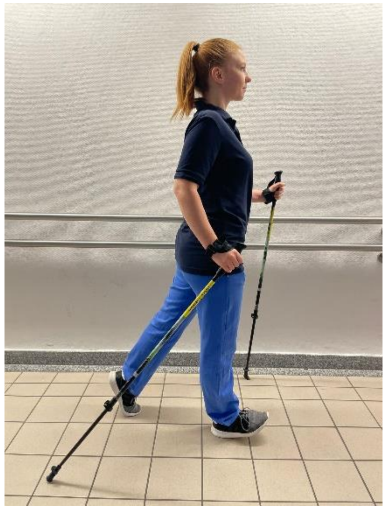 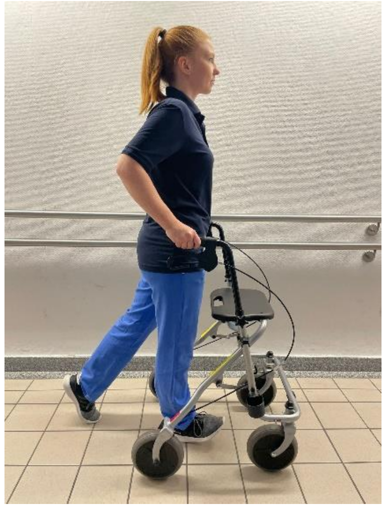 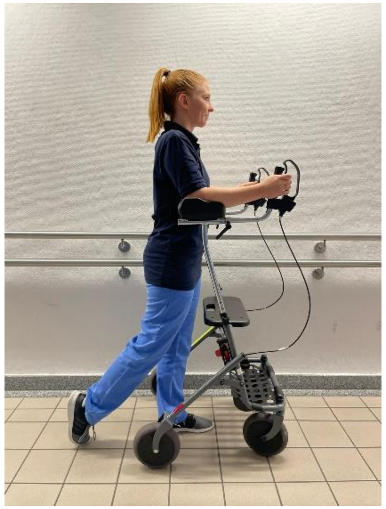

Gait training with assistance:

- Use gait aids such as one or two walking sticks, Nordic walking sticks, a walker or a forearm walker
- Walk with the help of another person (hooking or holding hands)
- Walk short distances with an assistant and walking aid with regular breaks to sit down
- Focus on stride width/size/height, proper heel rolling, posture, arm swing, and walking speed
- Count steps while carrying out daily activities, e.g., while walking from the bed to the toilet, and try to reduce the number of required steps (by taking larger ones)
- Monitor how long you can walk before a break is needed

Tip: If you develop small steps when trying to start walking or turn around (freezing of gait), either count 1-2-3 out loud and start walking on 3 (audible cue), or deliberately try to step over a tile joint or lines on the floor (visible cue).

Attention: Safety first, please practice under the supervision of another person, if feeling unstable.

6. Gait (Level 2)

Walking without assistance

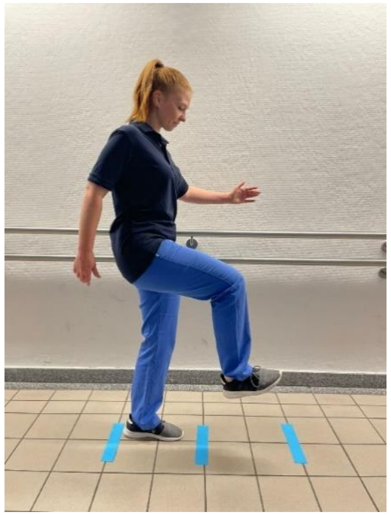

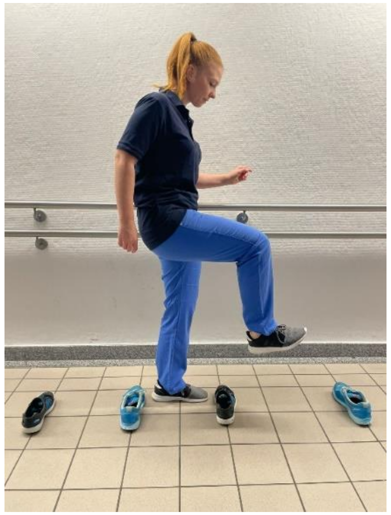

Gait training without assistance:

- Take long walks & walk outdoor
- Walk on different surfaces (meadow, forest ground, cobblestones, slopes)
- Walk with start-stop command
- Walk around objects (e.g., slalom around shoes) or walk over obstacles (e.g., adhesive strips, pens or shoes)
- Walk while performing additional tasks (counting or calculating number sequences, enumerations such as female first names/animals with certain initial letters/types of vegetables/car brands, memory tasks such as listing all the food eaten the previous day)
- Walk forwards, backwards & sideways (please keep close to a wall or an assistant to hold on to, if necessary)

Tip: If you develop small steps when trying to start walking or turn around (freezing of gait), either count 1-2-3 out loud and start walking on 3 (audible cue), or deliberately try to step over a tile joint or lines on the floor (visible cue).

Attention: Safety first, please practice under the supervision of another person, if feeling unstable.

7. General recommendations

**Orthostatic intolerance**	- Transfer slowly (e.g,. turn over in bed, sit up, stand up) & then wait a short time before moving on<br>- Perform circulation exercises at regular intervals<br>- Drink a large glass of water shortly before physical activities (e.g., getting up, walking, training)
**Pain**	- Identify painful activities<br>o Observe which part of the body hurts during which movement. Is there pain at rest?<br>- Adjust movements<br>o Is it better to perform movements more slowly or in a simpler way than not performing them at all?<br>- Perform stretching maneuvers in case of painful muscle contractions (e.g., shoulder/neck area)
**Freezing of gait**<br>(i.e., small steps on the spot without moving forward)	- Observe which situations lead to movement freezing, e.g.,<br>o Attempt to start walking<br>o Walking through narrow passages or pathways<br>o Turning around while walking or making tight turns<br>o Start-stop situations while walking (e.g., at the traffic light)<br>o Turning over in bed or getting up from a chair<br>o Poorer daily condition<br>o Stressful situations (often in public)<br>- Exploit audible cues<br>o Counting: count 1-2-3 out loud and start walking on 3<br>o Move to the metronome beat (metronome, metronome smartphone app, egg timer every third beat)<br>o Move to the rhythm of the music (play your favorite music and walk to the beat)<br>- Exploit visual cues

	○ Climb over something (shoe of an assistant, grouting strip of the tiles) to start<br>○ Objects that should be tapped when turning over & standing up (Post-It, handkerchief, smartphone, table)
**Other tips**	- Perform movements „large“, fast and rhythmically<br>- Avoid distraction in potentially dangerous situations, i.e., while walking, climbing stairs, in case of dizziness<br>- Find out which movements, leisure activities and sports you enjoy most and do them more often (table tennis, yoga, handcrafts, dancing, walking, gardening)<br>- Count out loud for movements that are difficult (e.g., count 1-2-3 out loud and start walking on 3)<br>- Stretch and practice regularly<br>- Maintain independence: do as much independently as possible under safe conditions<br>- Raise your feet well while walking<br>- Take breaks: take shorter and more frequent breaks rather than longer and less<br>- Breath control: inhale deeply through the nose and exhale slowly through the mouth
