# Supplementary material for: MeDeMSA care study protocol: developing personalized best medical care with integrated mobile palliative and telemedicine support for individuals with multiple system atrophy
Source: J Neural Transm (Vienna). 2025 May 24;133(5):903–17. doi: 10.1007/s00702-025-02933-z (PMC13216168; doi:10.1007/s00702-025-02933-z)
Supplement: Supplementary file 3 — Supplementary file3 (PDF 2986 KB) [file 702_2025_2933_MOESM3_ESM.pdf]

MeDeMSA Care study protocol – Supplementary Material 3

Occupational Therapy Operational Protocol

Table 1 MeDeMSA Care Occupational Therapy Operational Protocol Level 1 consists of basic occupational therapy exercises designed for individuals with greater disability. Level 2 includes more difficult exercises intended for individuals with lower levels of disability, as determined by a functional assessment. An illustrated exercise catalogue is provided hereinafter.

	Level	Test results	Therapy content
1. Training Activities of Daily Living	Level 1	**Test results OSA-Item:**<br>Difficult to very difficult and important to very important	- General recommendations on adapting the home environment to increase safety (e.g.: removing carpets, placing visual cues on the floor to prevent freezing, handholds, ...) and possible aids, including dressing aids
		"I am physically able to do what is important."	- Practice ADL sequences that cause difficulties: getting dressed (socks, shoes, clothes, fasteners)<br>- Develop and use dressing techniques/strategies (e.g.: use of cueing strategies in bADLs - cueing cards)
		"I take care of myself."<br>(personal hygiene)	- Practice personal hygiene routine (e.g.: brushing teeth, combing hair, showering)<br>- Discuss tips and tricks for self-care and personal hygiene
		"I pay attention to my basic needs."	- Tips and tricks: self-sufficiency like preparing food<br>- Advice on aids - dressing aids

	Level	Test results	Therapy content
1. Training Activities of Daily Living	Level 2	**Test results OSA-Item:**<br>Difficult to very difficult and important to very important	- General recommendations on adapting the home environment to increase safety (e.g.: removing carpets, placing visual cues on the floor to prevent freezing, handholds, ...) and possible aids, including dressing aids
		"I manage my finances."<br><br>"I recognize problems and can solve them."	- Encourage the use of external memory aids in everyday life: calendars, cell phone reminders, Post-It notes, tablet boxes<br>- Discuss tips and tricks for using memory aids successfully<br>- Training of activities of domestic life or assistive devices
		"I have a daily routine that suits me."	- Discuss tips and tricks for dealing with fatigue
		"I keep my household in order."	- Discuss tips and tricks for managing household activities
2. Writing Training	Level 1	**Test results OSA-DLS-Item:**<br>Difficult to very difficult and important to very important<br><br>"I am physically able to do what is necessary." (Writing)<br><br>NHPT out of the normal range	- Practice the signature<br>- Provide writing aids: large handle thickenings, special handles (try out different materials, forms and thicknesses)<br>- Discuss tips and tricks for writing and enhancing communication<br>- Encourage electronic writing aids when writing is no longer possible (cell phone signature, PC, voice recognition)<br>o Discuss tips and tricks for alternatives to handwriting and signatures<br>- Writing folder with lined paper and swing exercises

	Level	Test results	Therapy content
2. Writing Training	Level 2	**Test results OSA-DLS-Item:**<br>Difficult to very difficult and important to very important<br><br>"I am physically able to do what is necessary." (Writing)<br><br>NHPT out of the normal range	- Practice writing letters, emails, diaries, etc. (on paper, with cell phone, on laptop)<br>- Operating/changing the settings on the PC/mobile phone<br>- Writing aids: small thickened grips, special pens (ergonomic pens)<br>- Developing visual cueing strategies for writing, i.e., specified lines<br>- Tips and tricks: writing and communication, i.e., writing speed, posture, use of aids<br>- If of interest, offer a 5-weeks writing exercise program
3. Fine Motor Training	Level 1	BBT in normal range<br><br>NHPT out of the normal range<br>(> 50 seconds)	- Arm and hand training in connection with meaningful activities and meaningful context for the patient, if the disability degree permits it<br>- Fine motor training:<br>o Cross out six rows of zeros quickly<br>o Flip and collect coins: large coins<br>o Turning nuts onto screws: large nuts
	Level 2	BBT in normal range<br><br>NHPT out of the normal range<br>(> 50 seconds)	- Arm and hand training in connection with meaningful activities and meaningful context for the patients, if the disability degree permits it<br>- Fine motor training:<br>o Cross out zeros (different size and number)<br>o Flip and collect coins: different sized coins<br>o Turning nuts onto screws: nuts of different sizes

	Level	Test results	Therapy content
4. Upper Limb Gross Motor Training	Level 1	BBT out of the normal range<br>(≤ 20 cubes)<br><br>NHPT out of the normal range	- Motor training: transporting cups<br>- Bimanual exercises with towels, i.e., folding<br>- Targeted movements: small distances
	Level 2	BBTout of the normal range<br>(≤ 20 cubes)<br><br>NHPT out of the normal range	- Gross motor training: transporting a water bottle<br>- Bimanual exercises with towels, i.e., rolling<br>- Targeted movements: large distances
5. Upper Limb Strength Training	Level 1	Dynamometer < 10kg hand force	- Exercises with household sponges to strengthen hand muscles<br>- Exercises with an empty bottle to strengthen the arm muscles<br>- Exercise while standing: place an empty bottle on a shelf
	Level 2	Dynamometer ≥ 10kg manual force	- Exercises with therapy clay to strengthen the hand muscles<br>- Exercises filled bottle (0.5 liter) to strengthen the arm muscles<br>- Exercise while standing: place a bottle on a shelf (vary the filling of the bottle)

OSA DLS = Occupational Self-Assessment-Daily Living Scales; NHPT = Nine-hole-peg test; BBT = Box and Block Test; bADL = basic activities of daily living.

MeDeMSA Care illustrated occupational therapy exercise catalogue

1. Training Activities of Daily Living (Level 1) 7

For example: dressing with clue cards 7

1. Training Activities of Daily Living (Level 2) 8

Using memory aids 8

2. Writing Training (Level 1) 9

Writing with large grip thickening 9

2. Writing Training (Level 2) 10

Writing with small or without grip thickening 10

3. Fine Motor Training (Level 1) 11

Crossing out zeros 11

Flip large coins (or small discs) 12

Turning nuts onto screws 13

3. Fine Motor Training (Level 2) 14

Crossing out zeros 14

Flip and collect coins (or discs) of different sizes 15

Turning nuts onto screws 16

4. Upper Limb Gross Motor Training (Level 1) 17

Moving cups 17

Bimanual exercises with towel: folding 18

Targeted movement: small distances 19

4. Upper Limb Gross Motor Training (Level 2) 20

Moving a water bottle 20

Bimanual exercises with towel: rolling 21

Targeted movements: large distances 22

5. Upper Limb Strength Training (Level 1) 23

Exercises with a sponge 23

Exercises with an empty bottle 24

Upon standing place an empty bottle on a high shelf 25

5. Upper Limb Strength Training (Level 2) 26

Exercises with modeling clay 26

Exercises with modeling clay 27

Exercises with filled bottle 28

Upon standing place a bottle on a raised shelf 29

6. General Tips and tricks 30

1. Training Activities of Daily Living (Level 1)

For example: dressing with clue cards

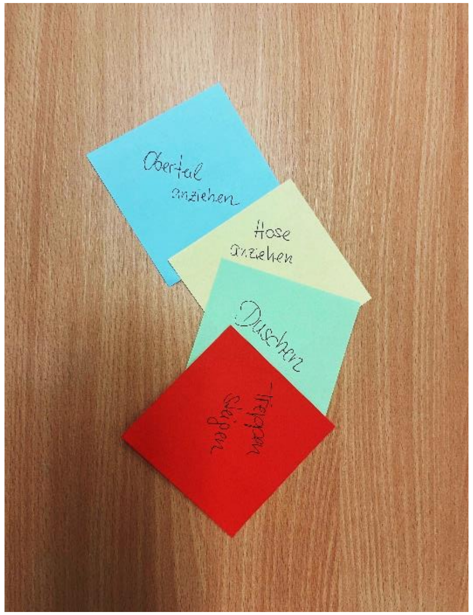

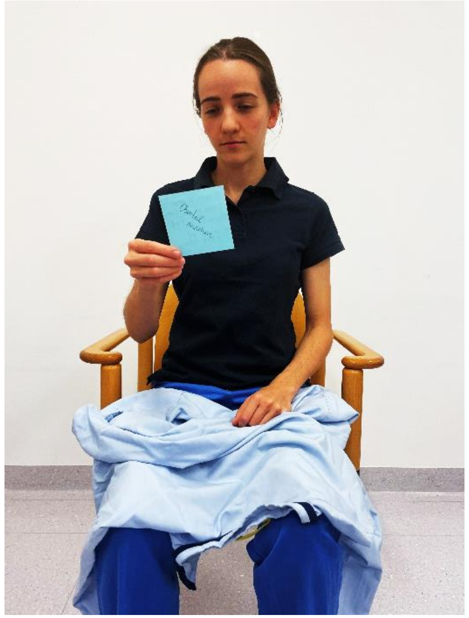

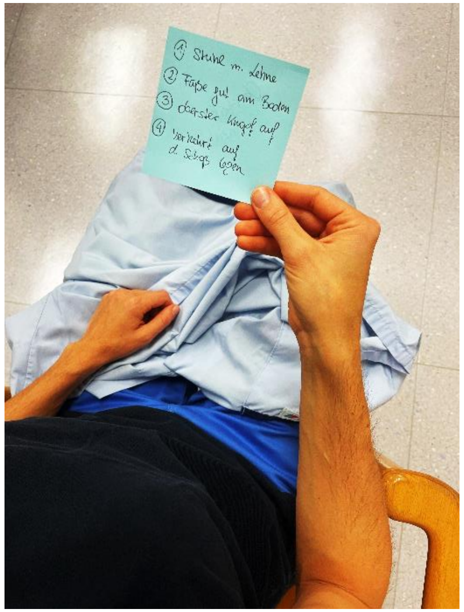

Everyday training:

- Practicing specific ADL sequences: dressing/personal hygiene/climb-up the stairs
- Use of dressing techniques or strategies for independent dressing, e.g., writing clue cards with sequential steps to wear trousers or shirts safely
- Discuss possible environmental adaptations to increase safety and comfort: e.g., dress in a safe sitting position, using a chair with back- and armrest

1. Training Activities of Daily Living (Level 2)

Using memory aids

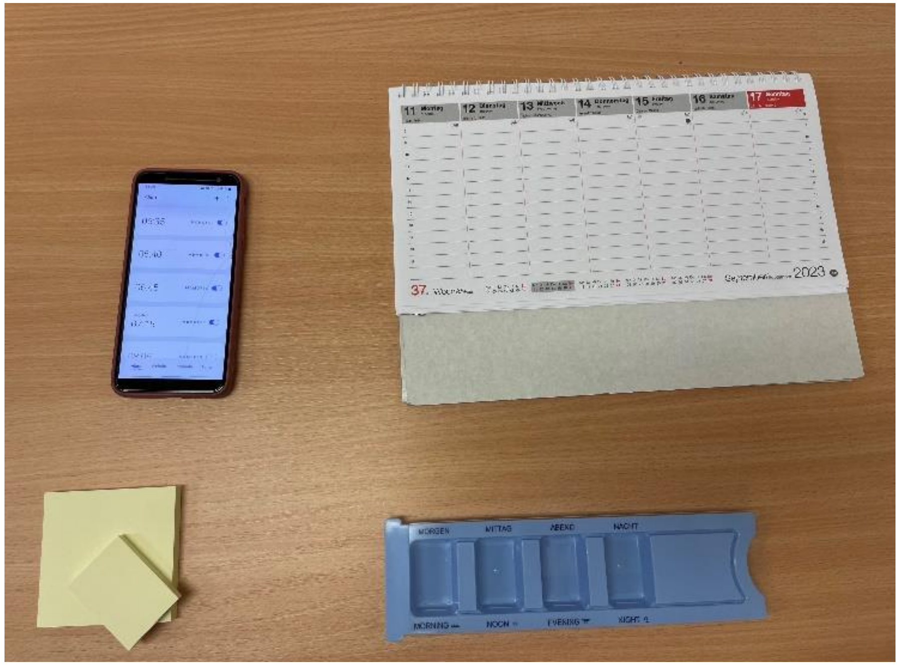

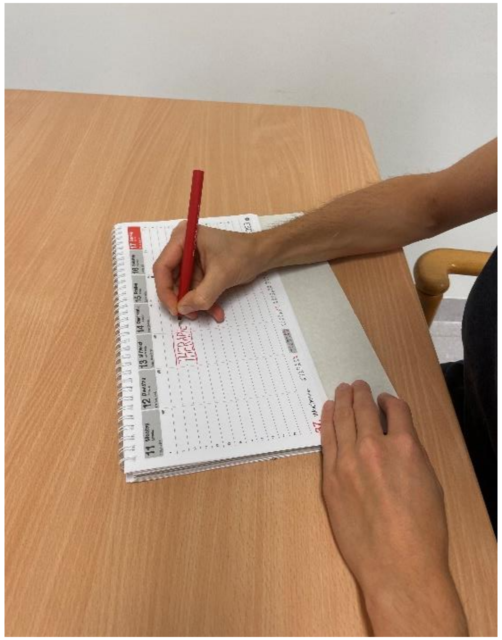

Memory aids:

- Introduce and encourage the use of memory aids, such as cell phone alarms for taking medications at prescribed intervals, calendars for appointments, Post-Its for reminders, and pill boxes
- Use of aids or training in activities for home life (see photo)

Further information: See "Tips and tricks" sheet - use of memory aids.

2. Writing Training (Level 1)

Writing with large grip thickening

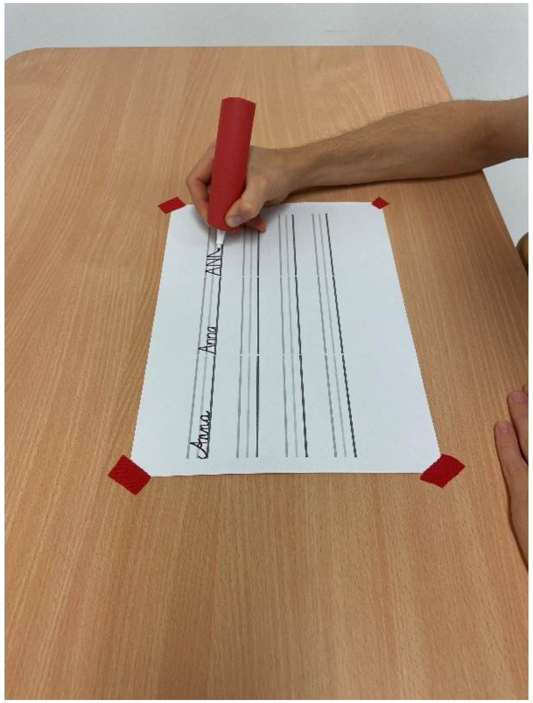

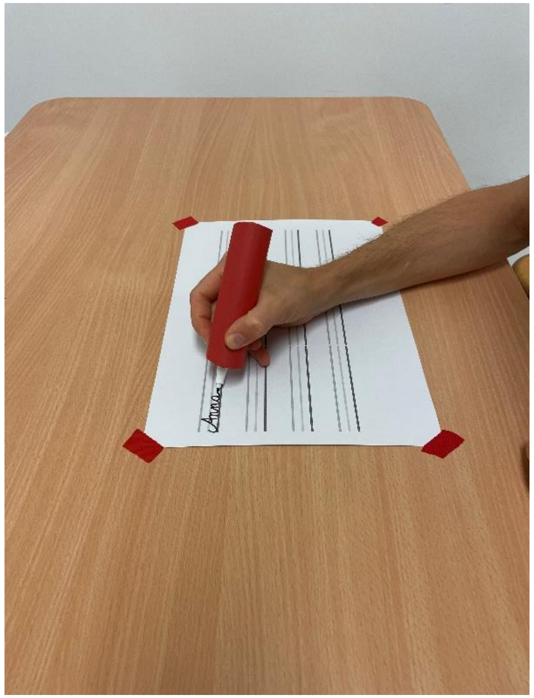

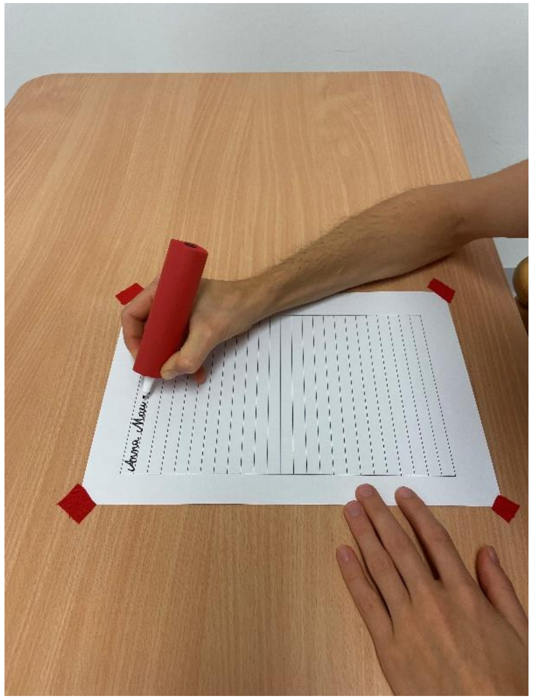

Writing training:

- Writing with thickened grips for pens
- Write on lined or squared paper and use the entire line height

Variant: Block letters are easier than italic letters, and capital letters are easier than lower-case ones

2. Writing Training (Level 2)

Writing with small or without grip thickening

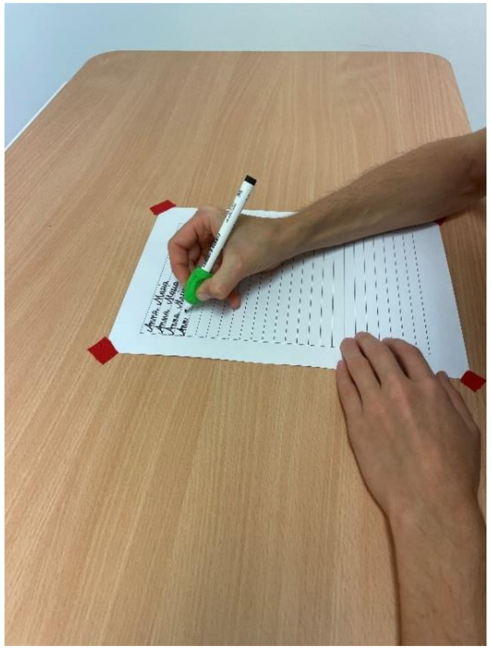

Writing training:

- Use pens and pencils with a thick or padded cover or a small, thickened grip so that you can hold them better
- Write on plain lined or squared paper to maintain the font size
- Use a clipboard or an anti-slip mat to prevent your paper from slipping
- Take time to write

Specific five-week writing training program.

3. Fine Motor Training (Level 1)

Crossing out zeros

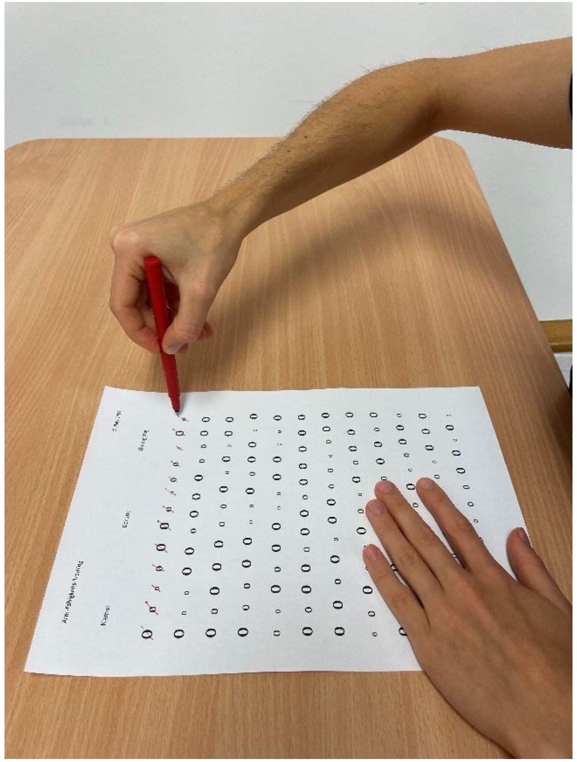

Cross out zeros:

- Sit at the table and place the template with zeros on the table (you may attach it with adhesive tape or keep it fixed with your free hand)
- Cross out six rows of zeros with a pen
- The arm and the hand with the pen should not touch the table

3. Fine Motor Training (Level 1)

Flip large coins (or small discs)

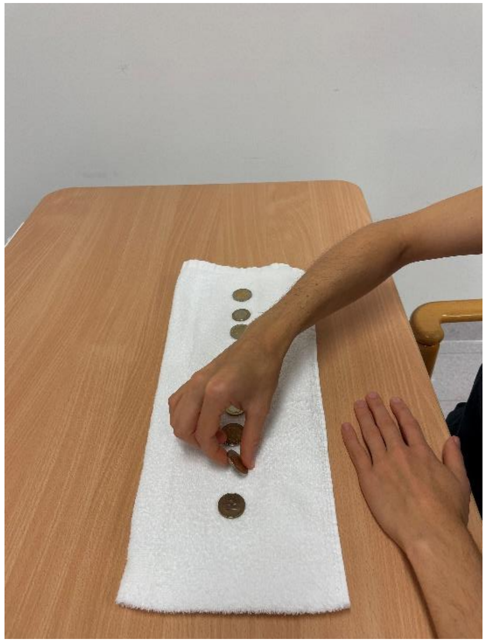

Flipping coins on the towel:

- Place 10 large coins (1 and 2 euros) in a row on a towel
- Flip the coins with your right hand as quickly as possible
- Change hands and repeat the exercise
- Repeat the sequence twice per side

3. Fine Motor Training (Level 1)

Turning nuts onto screws

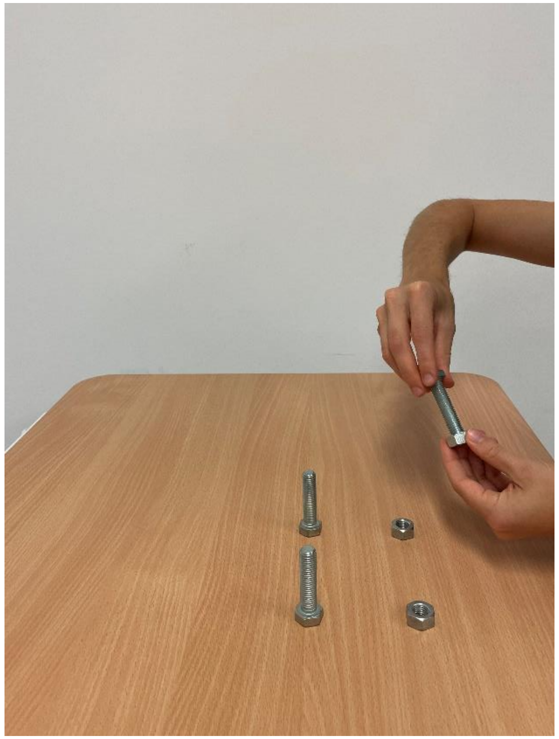

Turn large nuts onto large screws:

- Place three large screws with matching nuts on the table
- Turn nuts onto screws
- Repeat five times

3. Fine Motor Training (Level 2)

Crossing out zeros

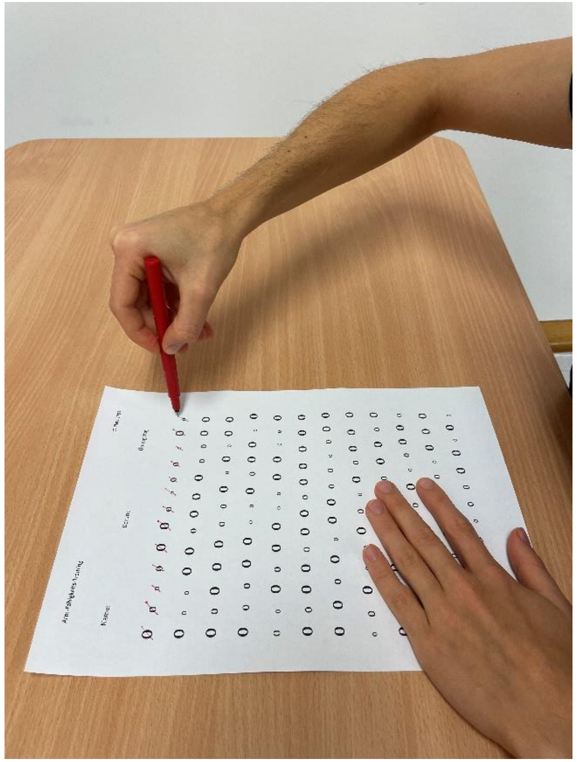

Cross out the zeros on an A4 sheet:

- Template with zeros is on the table (attach with adhesive tape or fix with your free hand)
- Cross out all zeros as quickly as possible with a pen
- The arm and the hand with the pen do not touch the table

3. Fine Motor Training (Level 2)

Flip and collect coins (or discs) of different sizes

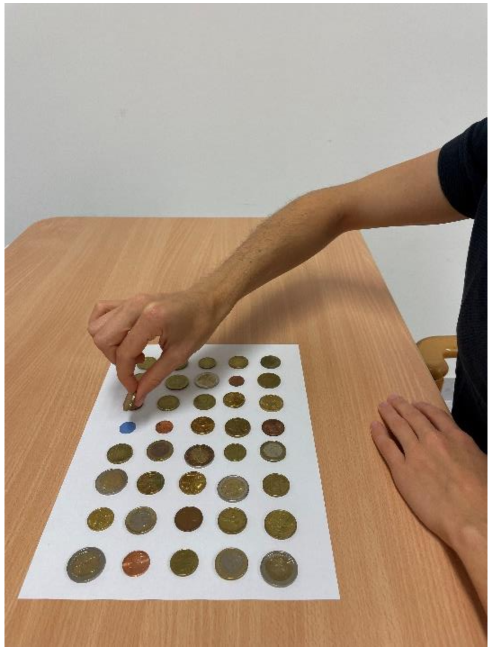

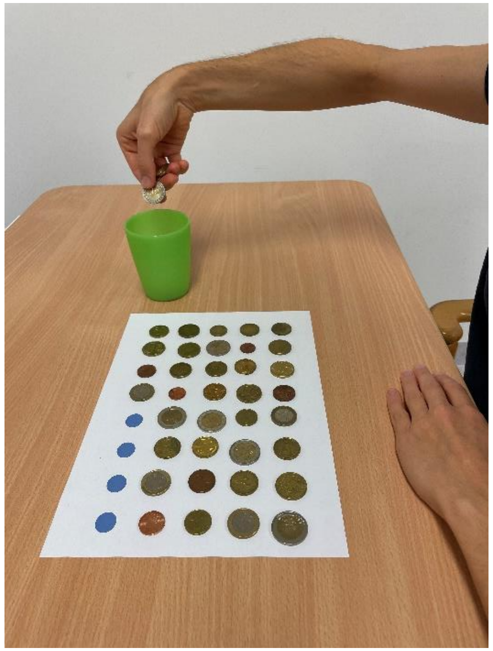

Turn over coins of different sizes:

- Place 40 coins of different sizes in five rows
- Flip the coins with your right hand (if right-handed) as quickly as possible and place them back on the respective marking
- Flip the coins with your left hand as quickly as possible

Pick up single coins and place them in a box or purse:

- Pick up to four coins one after the other and hold them in your hand
- Place the coins one after the other into a container (e.g., box or purse)

3. Fine Motor Training (Level 2)

Turning nuts onto screws

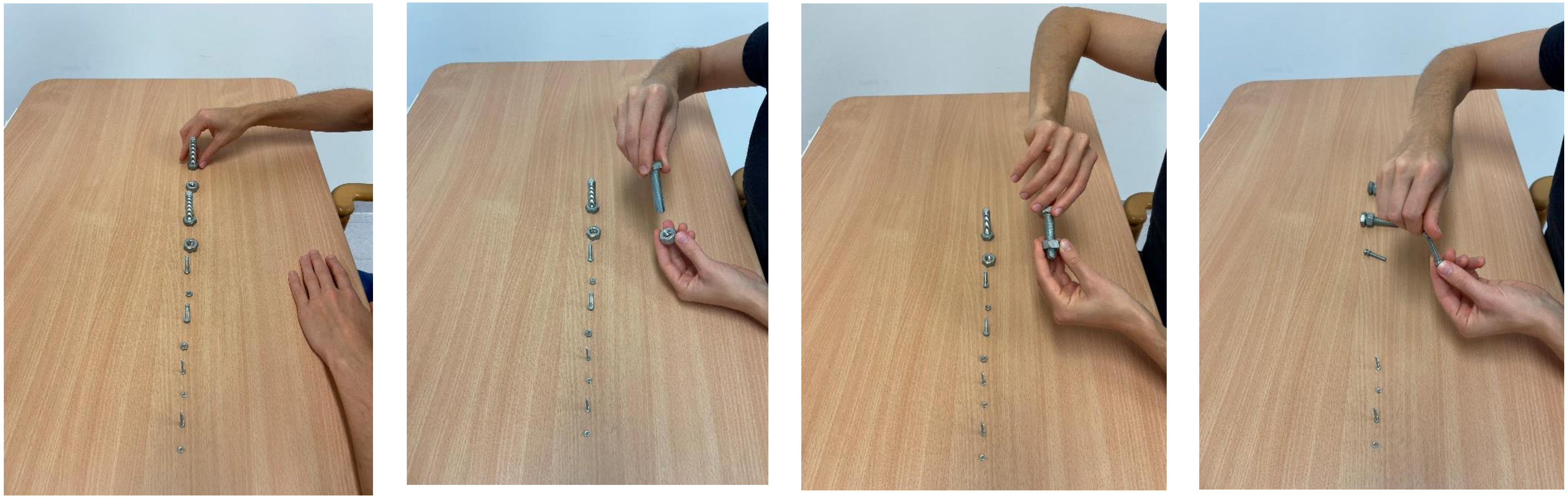

Screw the nuts onto the appropriate screws in different sizes:

- Place six screws in different sizes with matching nuts on the table
- Turn nuts onto screws, starting with the largest screw
- Remove the nuts again, starting with the largest one
- Repeat the sequence five times

4. Upper Limb Gross Motor Training (Level 1)

Moving cups

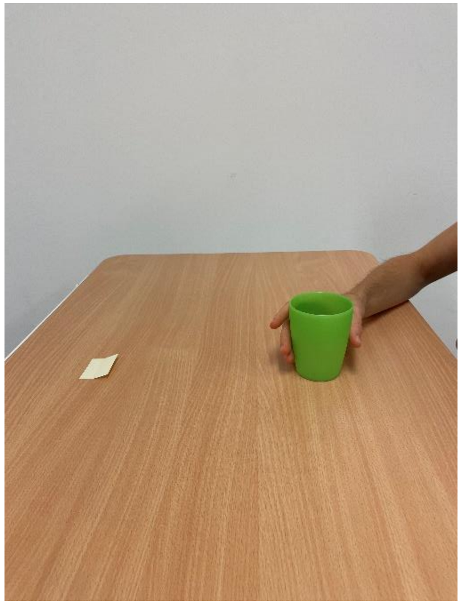
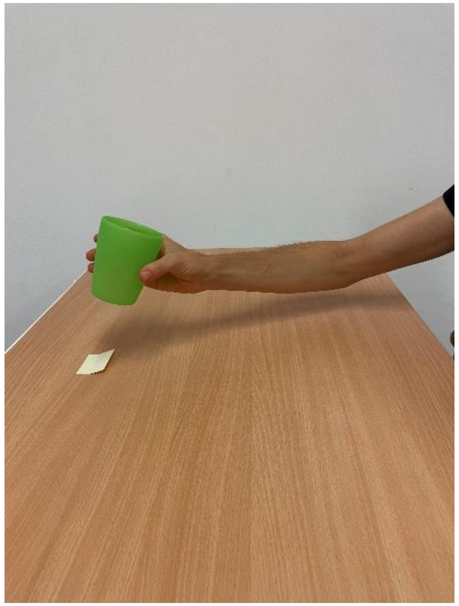
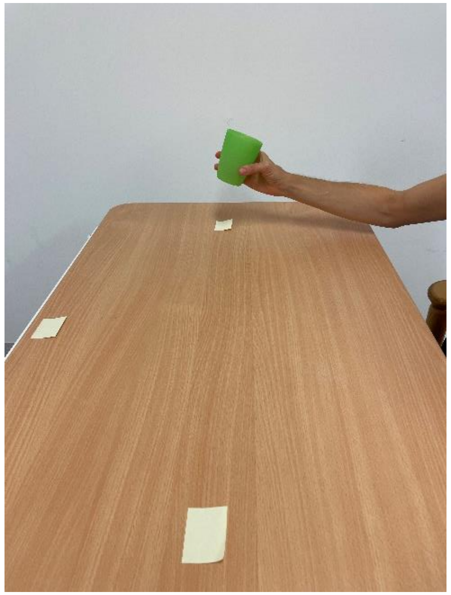
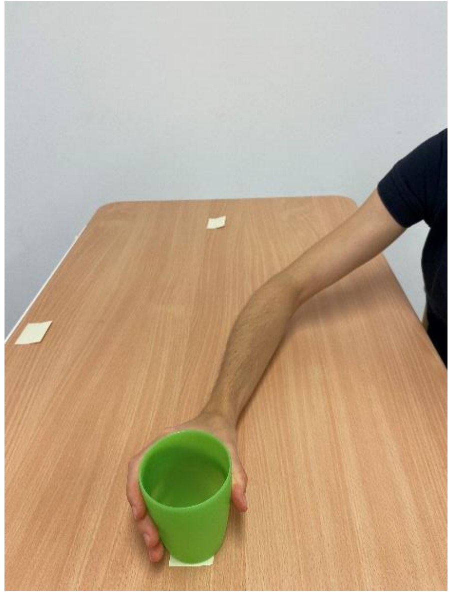

Place an empty cup on a marker in front of you:

- Grab a cup and lift it up
- Place the cup far forward
- Grab the cup again and put it back

Place an empty cup on markers at the side:

- Grab the cup and lift it up
- Place the cup on the left
- Grab the cup again and put it back

4. Upper Limb Gross Motor Training (Level 1)

Bimanual exercises with towel: folding

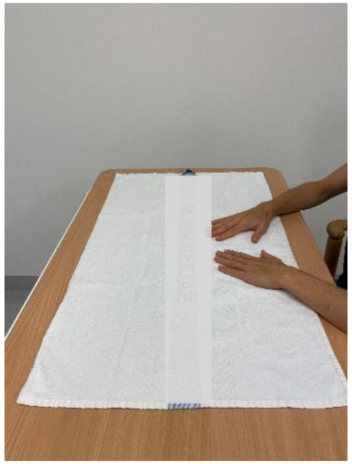
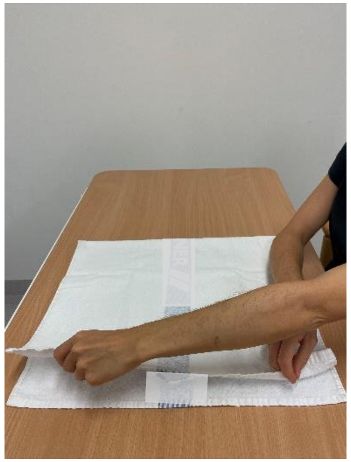
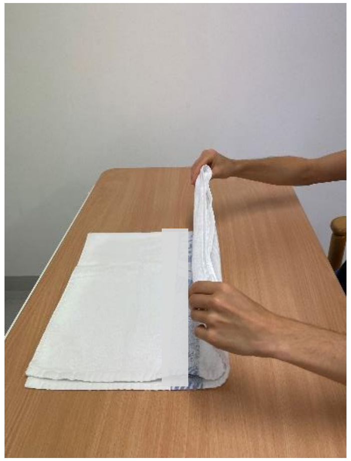
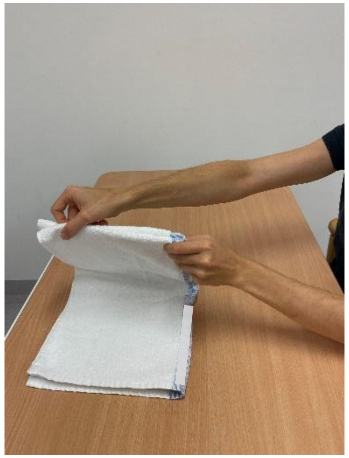
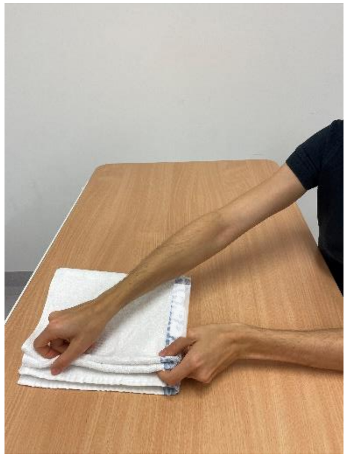

Fold the towel while sitting at the table:

- Spread out a towel on the table
- Fold the towel (two-three times depending on the size)
- Unfold it and repeat the exercise five times

4. Upper Limb Gross Motor Training (Level 1)

Targeted movement: small distances

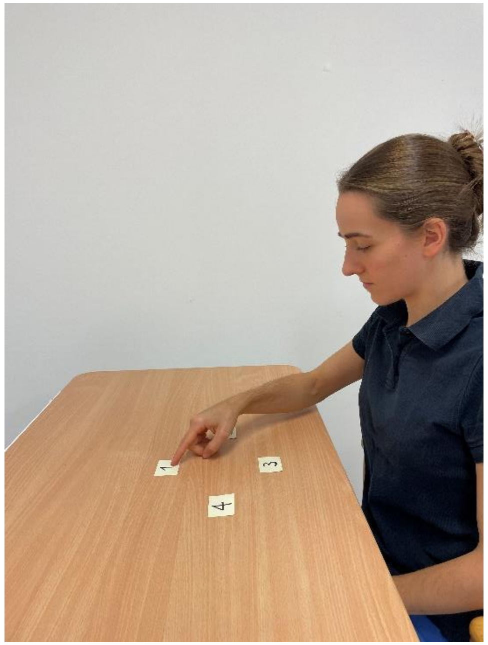

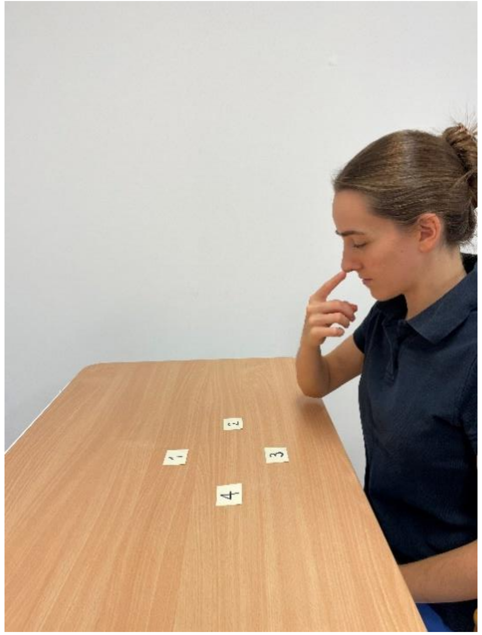

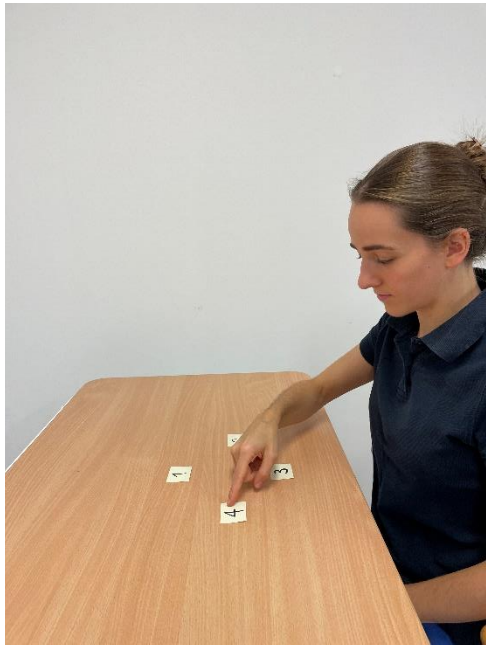

Target motor exercises with small distances:

- Stick small Post-Its (one-four) on the table as shown in the picture above
  Now touch in turn your nose and the Post-Its with your index finger, i.e., nose - Post-It 1 - nose - Post-It 2 - nose - Post-It 3 - nose - Post-It 4
- Repeat the sequence ten times

4. Upper Limb Gross Motor Training (Level 2)

Moving a water bottle

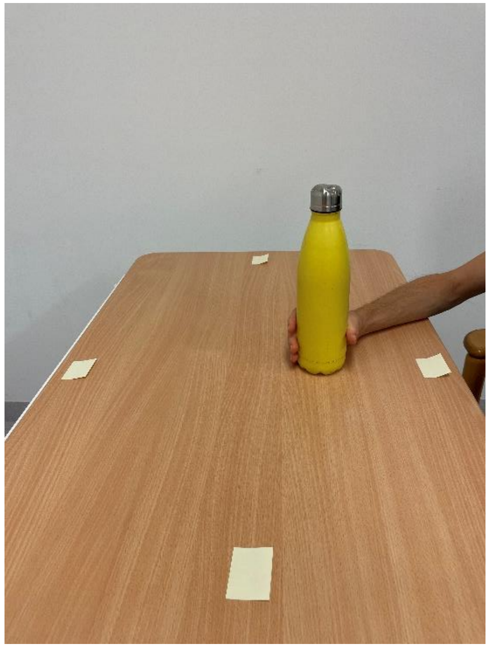

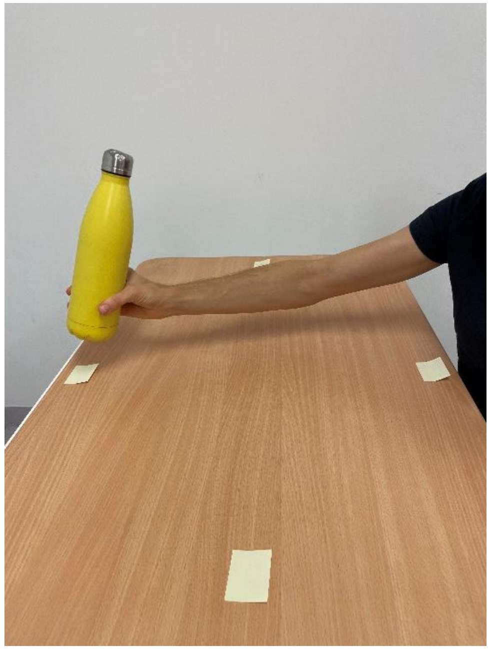

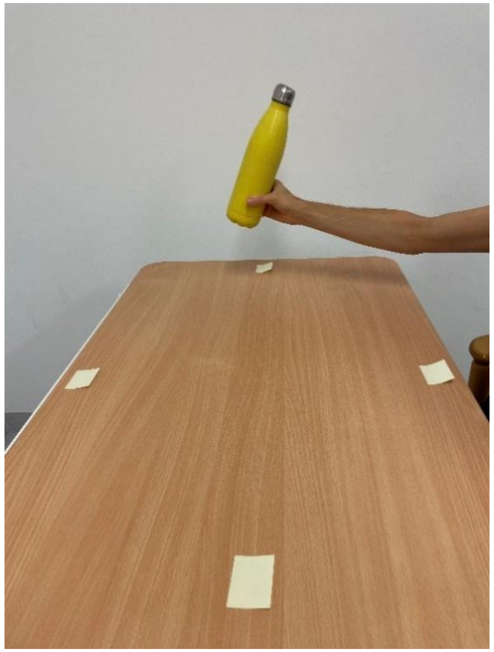

Move a water bottle for- and backward:

- Grab a bottle half-filled with water in front of you and lift it up
- Place the water bottle far forward
- Grab the water bottle again and put it back

Move a water bottle to the side:

- Grab the half-filled water bottle and lift it up
- Place the water bottle on the left
- Grab the water bottle again and put it back

Variation: Increase the weight of the object to increase difficulty (e.g., fill the water bottle to the top).

4. Upper Limb Gross Motor Training (Level 2)

Bimanual exercises with towel: rolling

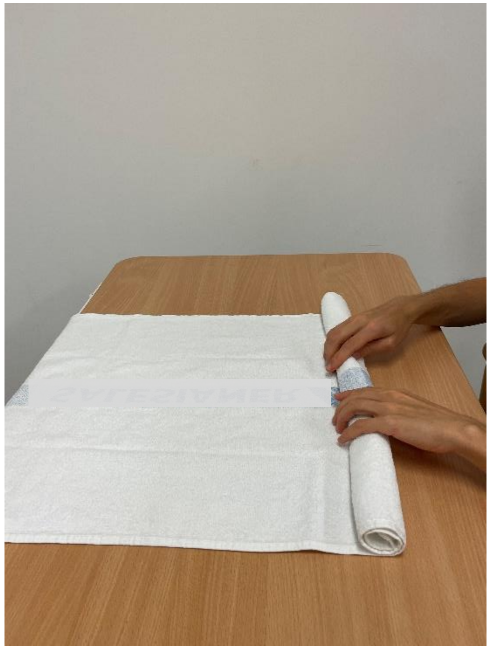

Roll up the towel at the table:

- Spread a towel on the table in front of you
- Roll up the towel from the short side

Everyday relevance: If you are able to, you can practice bimanual exercises by folding the freshly washed laundry.

4. Upper Limb Gross Motor Training (Level 2)

Targeted movements: large distances

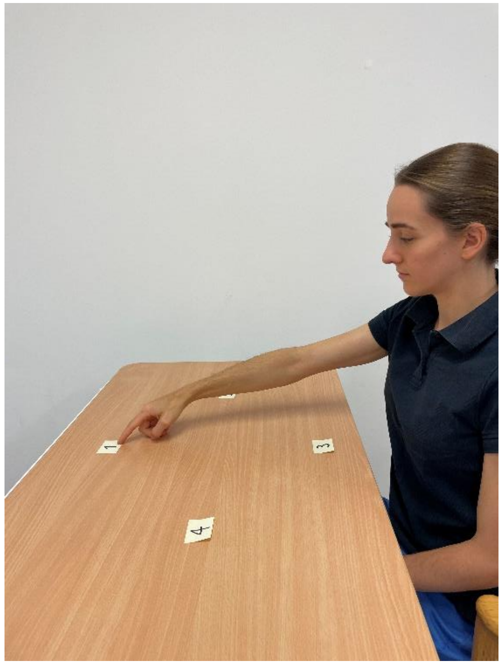

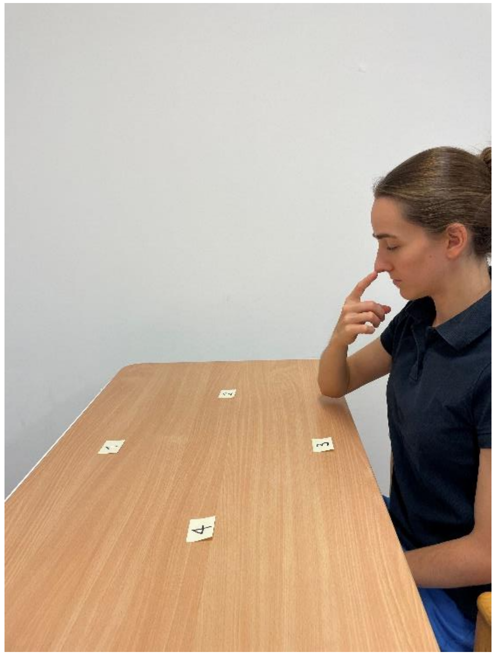

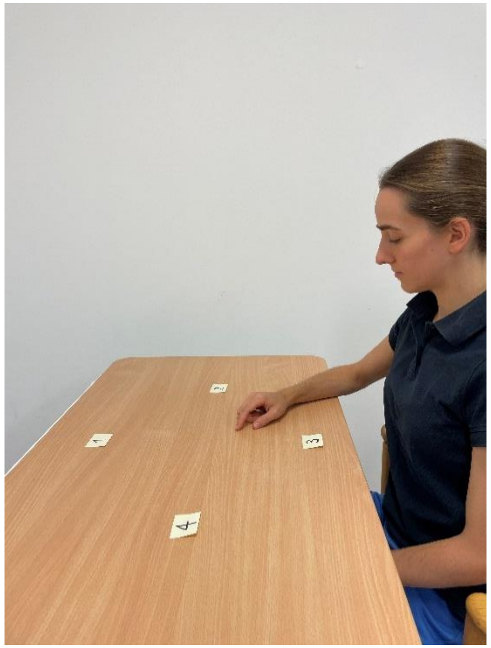

Target motor exercises with large distances:

- Stick small Post-Its (one to four) on the table as shown in the picture above
- Now touch in turn your nose and the Post-Its with your index finger, i.e., nose - Post-It 1 - nose - Post-It 2 - nose - Post-It 3 - nose - Post-It 4
- Repeat the sequence ten times

5. Upper Limb Strength Training (Level 1)

Exercises with a sponge

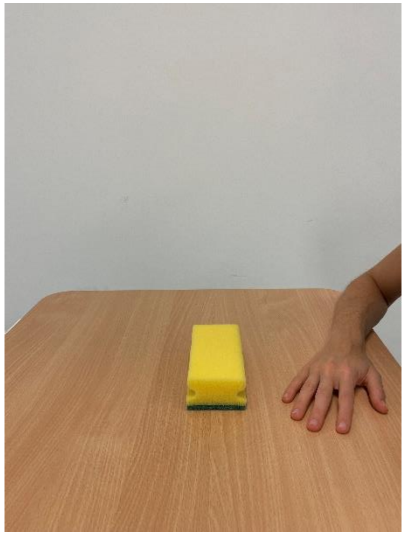

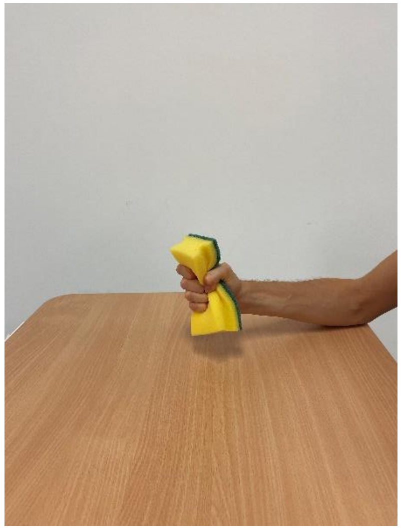

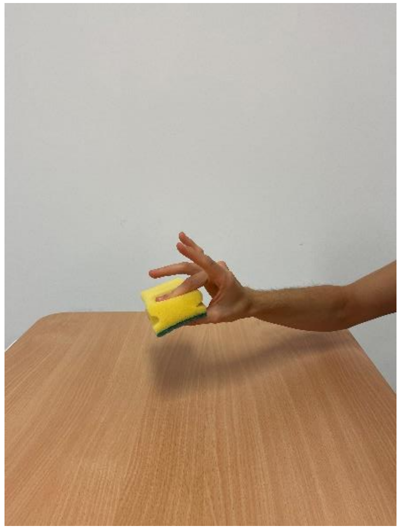

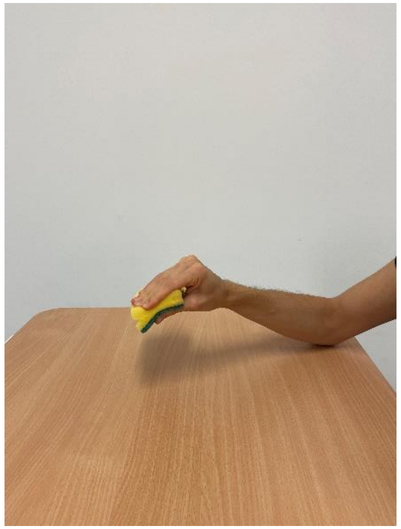

Strengthening exercises with a sponge:

- Take a sponge in your hand, form a fist and squeeze firmly
- Repeat ten times
- Take the sponge between your fingers and squeeze together
- Repeat ten times
- Place sponge between outstretched fingers and thumbs and squeeze together
- Repeat ten times

5.Upper Limb Strength Training (Level 1)

Exercises with an empty bottle

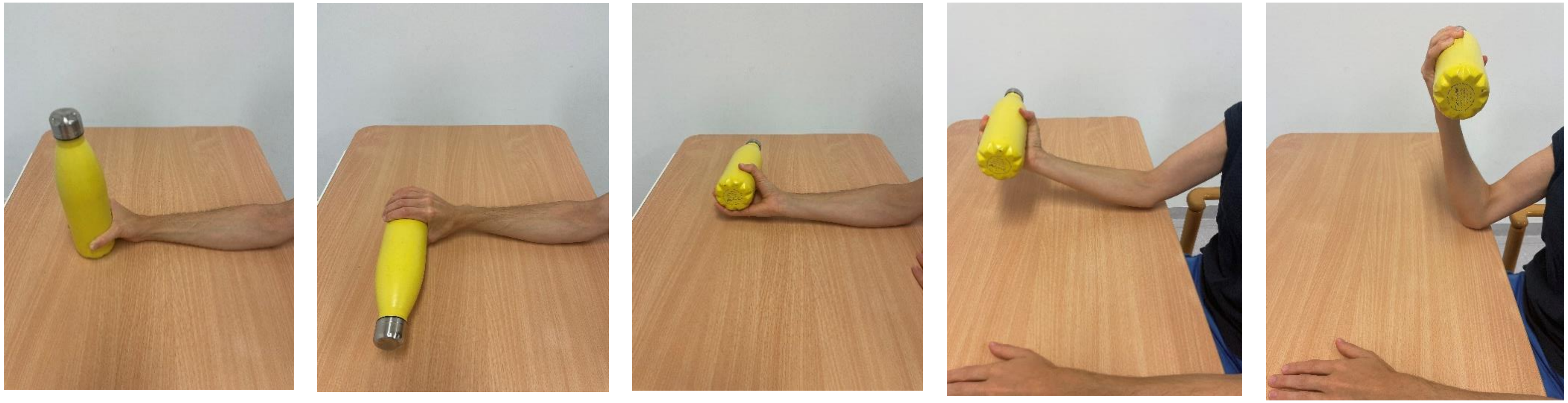

Exercises with an empty bottle:

- Lay a bottle on the table and flip it in both directions
- Move the bottle up and down by keeping your elbow on the table

Variation: Increase the weight of the object to increase difficulty (e.g., progressively fill the bottle with water).

5.Upper Limb Strength Training (Level 1)

Upon standing place an empty bottle on a high shelf

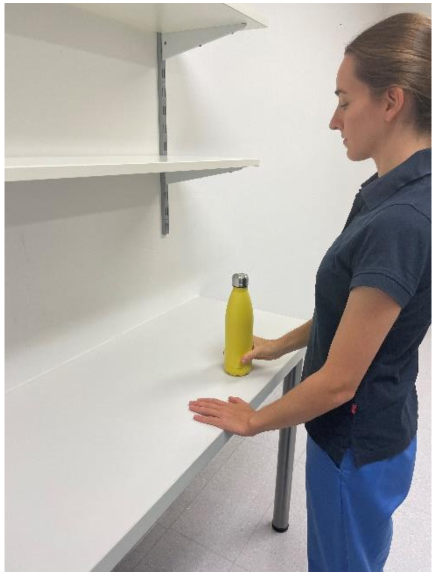
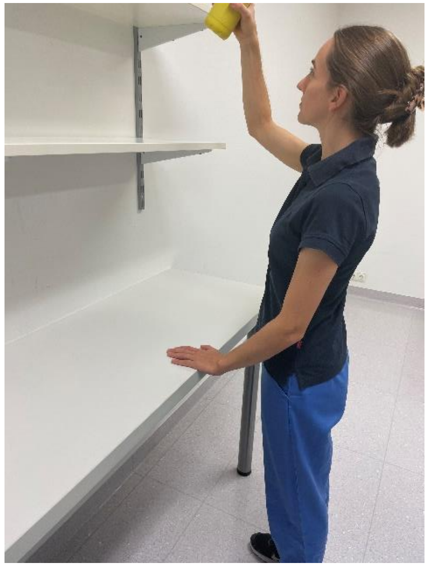
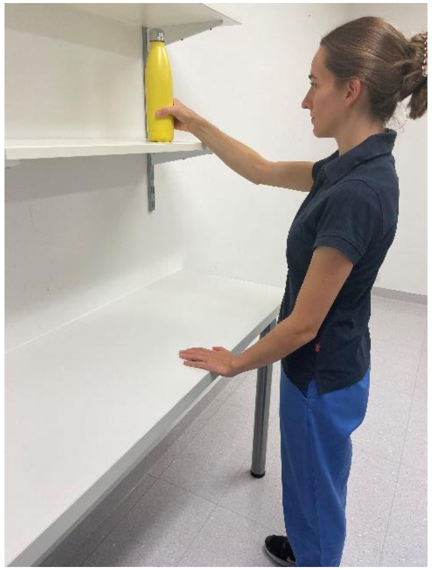

Everyday standing training - place a bottle on a high shelf:

- Practice specific daily sequences: grab a bottle and place it on a high shelf; then take the bottle down again and repeat the exercise five to ten times

Safety first:

- Feel free to hold on to the table or other stable surface
- Only do this exercise if you can stand on your own

Variation: Increase the weight of the object to increase difficulty (e.g., progressively fill the bottle with water).

5. Upper Limb Strength Training (Level 2)

Exercises with modeling clay

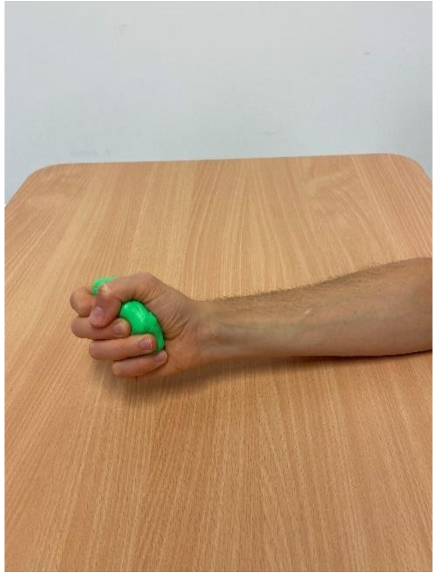
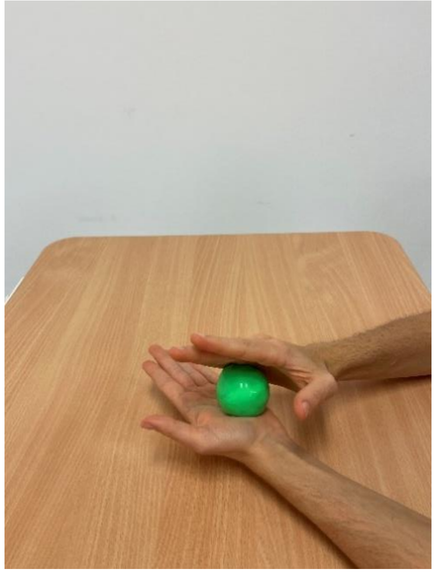
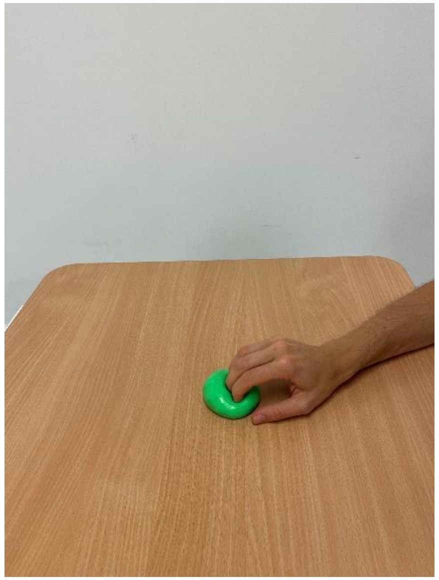
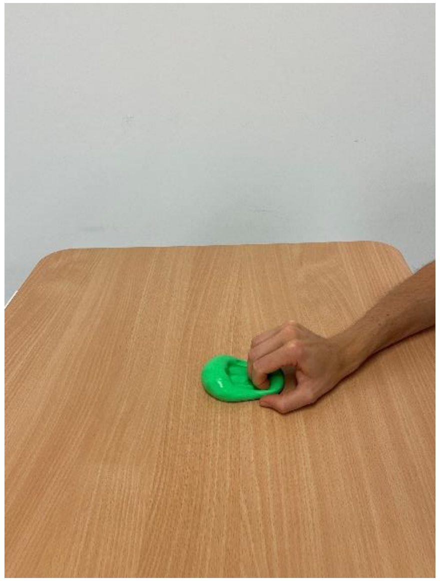

Exercises with modeling clay:

- Take the dough in your hand and squeeze it by forming a fist
- Repeat ten times
- Shape the dough into a ball and slowly press your fingers into it
- Pull the dough towards yourself with your fingers

5. Upper Limb Strength Training (Level 2)

Exercises with modeling clay

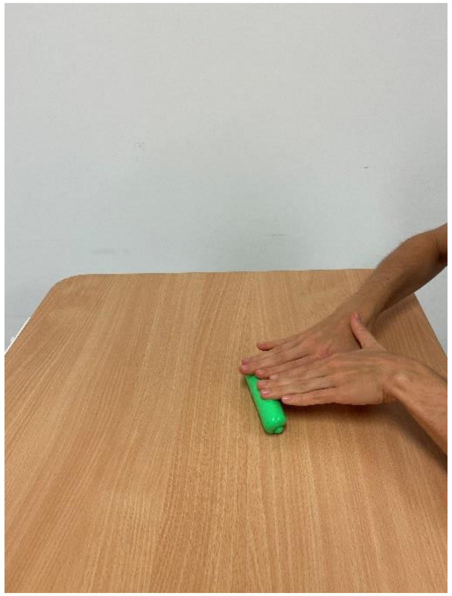
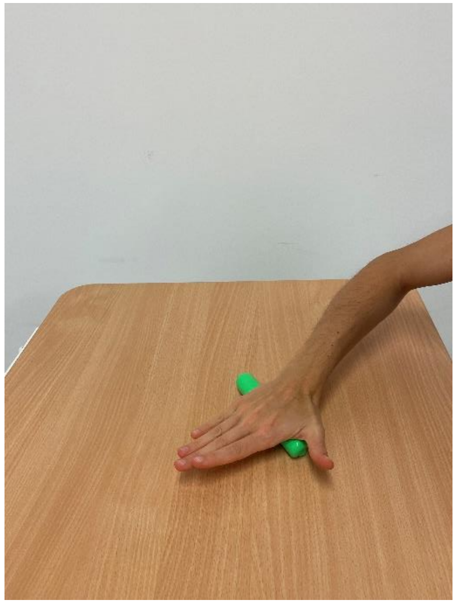
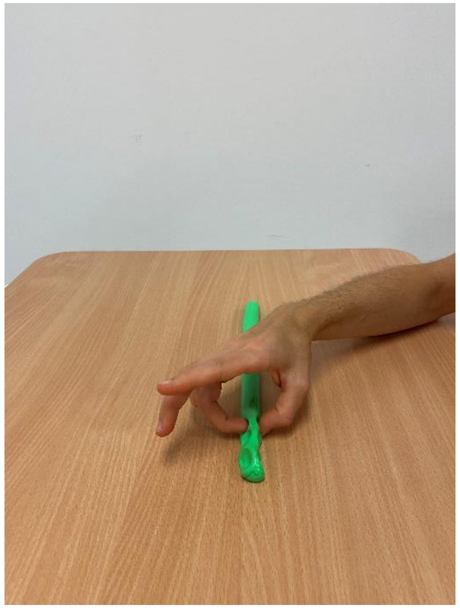
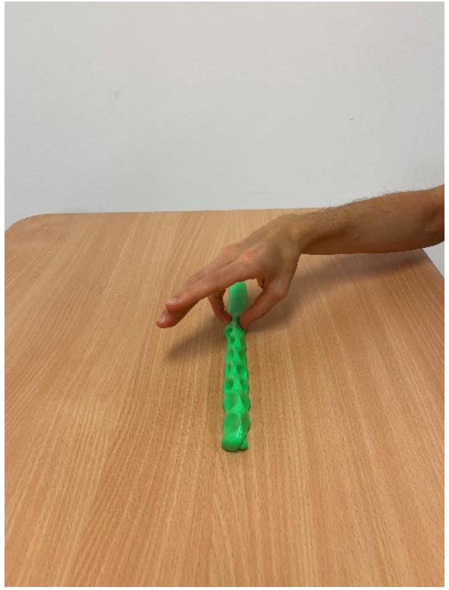

Exercises with modeling clay:

- Shape the dough into a string/sausage with both hands or with one hand
- Press your fingers alternately into the dough as shown in the picture above

5. Upper Limb Strength Training (Level 2)

Exercises with filled bottle

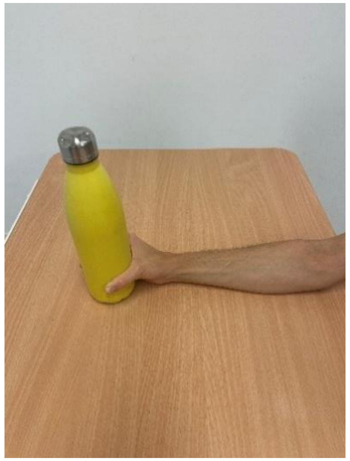
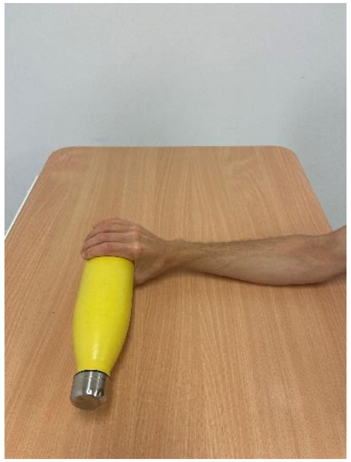
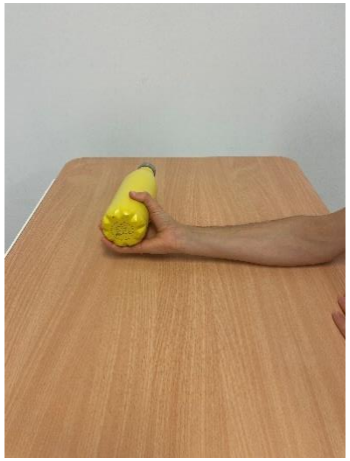
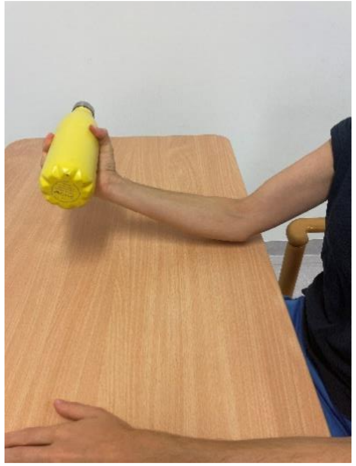
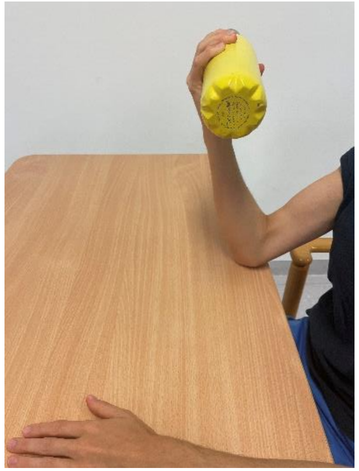

Exercises with a half-filled bottle:

- Lay a bottle on the table and flip it in both directions
- Move the bottle up and down by keeping your elbow on the table

Variation: Increase the weight of the object to increase difficulty (e.g., fill up the bottle with water).

5. Upper Limb Strength Training (Level 2)

Upon standing place a bottle on a raised shelf

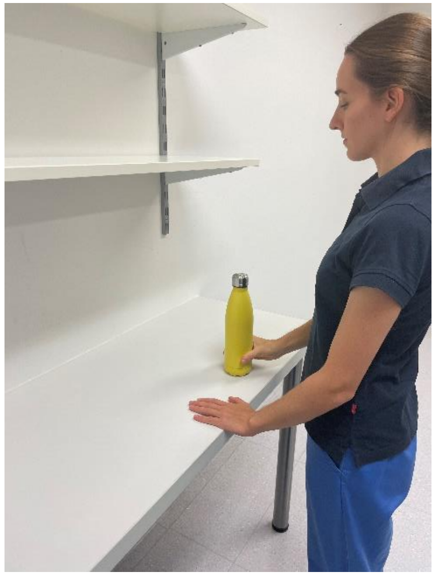

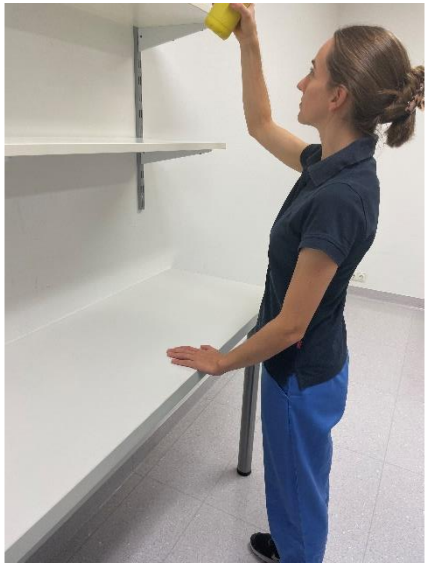

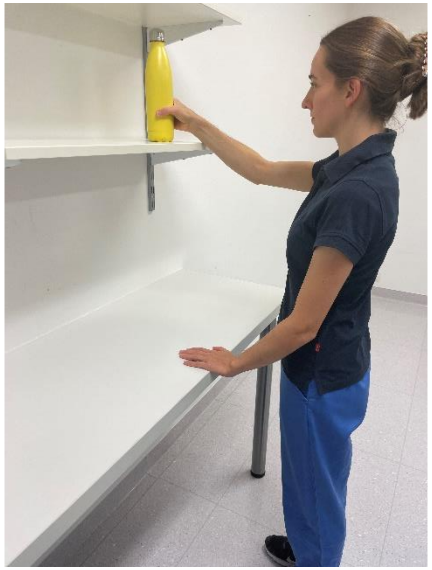

Everyday standing training - place a bottle on a raised shelf:

- Practice specific daily sequences: grab a bottle and place it on a high shelf
- Then take the bottle down again
- Repeat the exercise ten times

Variation: Increase the weight of the object to increase difficulty (e.g., fill up the bottle with water); adjust the height of the shelf; support with one hand or possibly with an assistant for additional safety.

6. General Tips and tricks

Tips and tricks	
**Self-care and personal hygiene**	- Stay active, but accept help if necessary<br>- Learn and practice dressing techniques<br>- Consider the use of aids<br>- Look after yourself: taking breaks, relaxing and getting enough sleep are important for your well-being
**Household management**	- Consider the use of aids<br>- Adapt household activities<br>- Schedule breaks<br>- Take advantage of help offers from family and friends for shopping and cooking<br>- Look for household support, discuss options with social workers, if needed
**Dealing with fatigue**	- Assess the extent to which fatigue affects your everyday life. Try to record your tasks and times with the help of a diary<br>- Prioritize your tasks to make the best use of your limited energy<br>- Energy management: distribute energy reserves throughout the day and save energy<br>- Plan activities ahead to fit well in the daily routine<br>- Observe sleep hygiene: avoid short naps throughout the day. If you are very tired during the day, plan a nap in the afternoon, which should not last longer than 40 minutes and should ideally be in bed
**Writing and communication**	- Warrant a good seating position: comfortable and upright<br>- Secure sufficient lightning<br>- Try out different aids to improve writing<br>- Write slowly. Think: "I should write slowly and smoothly"<br>- If writing is no longer possible, consider using alternative electronic methods, e.g., mobile phone signature<br>- Use PCs or smartphones, ask for support for setting-up software, apps and operating system, if encountering difficulties

**Advice for relatives**	- General tips for relatives: Positioning and posture (e.g., in a wheelchair or bed), information on the use of aids, support for staying active, safety aspects (removing tripping hazards), nutrition and hydration, own health, when to look for specialist support
