# Supplementary material for: MeDeMSA care study protocol: developing personalized best medical care with integrated mobile palliative and telemedicine support for individuals with multiple system atrophy
Source: J Neural Transm (Vienna). 2025 May 24;133(5):903–17. doi: 10.1007/s00702-025-02933-z (PMC13216168; doi:10.1007/s00702-025-02933-z)
Supplement: Supplementary file 4 — Supplementary file4 (PDF 666 KB) [file 702_2025_2933_MOESM4_ESM.pdf]

MeDeMSA Care study protocol – Supplementary Material 4

Speech Therapy Operational Protocol

Table 1 MeDeMSA Care Speech Therapy Operational Protocol The individual interventional needs are determined by the results of the in-person functional assessment.

	Area	Test results	Therapy content
1. Dysarthria	Posture	Impaired in the FDA	- Optimizing posture for speaking<br>- Posture training while sitting
	Breathing	Impaired in the FDA	- Awareness of resting and abdominal breathing<br>- Lengthening the expiration<br>- Implementing breaks to breathe deeply<br>- Diaphragm activation<br>- Develop an appropriate muscular tension<br>- Breathing and speaking
	Oral motor skills and facial expression	Impaired in the FDA	- Development of non-linguistic movements (tongue/lip motor skills and facial expressions)<br>- Autonomous movement sequences<br>- Motor exercises relevant to daily life

	Area	Test results	Therapy content
1. Dysarthria	Voice	Impaired in the FDA	- Phonation initiation<br>- Training the use of the voice<br>- Changing the tone holding time<br>- Modulating the voice<br>- Training of the register transitions<br>- Training of the medium speaking voice range<br>- Training the melody of the voice
	Speech articulation	Impaired in the FDA	- Oral motor exercises<br>- Diadochokinesia of the voice<br>- Sound processing<br>- Training the intentional adjustment of articulatory target movements
	Intelligibility (focus on loudness and volume)	Impaired in the FDA	- Changing the speech tempo<br>- Changing the speech volume<br>- Shortening the sentences<br>- Widen the jaw opening<br>- Clearness at syllable, word, sentence and text level

	Area	Test results	Therapy content
**2. Dysphagia**	Eating behavior	MUCS impaired<br>MDT-PD impaired<br>FEES impaired	- Advice for patients and relatives regarding eating behavior in daily situations
	Posture	MUCS impaired<br>MDT-PD impaired<br>FEES impaired	- Retraining the correct eating starting position<br>- Preparatory posture exercises for food intake
	Maintenance measures	MUCS impaired<br>MDT-PD impaired<br>FEES impaired	- Training a timely and residue-free swallowing act<br>- Training laryngeal protective mechanisms
	Compensatory measures	MUCS impaired<br>MDT-PD impaired<br>FEES impaired	- Training laryngeal protective mechanisms<br>- Training compensatory swallowing maneuvers (e.g., chin down, etc.)
	Dietary measures	MUCS impaired<br>MDT-PD impaired<br>FEES impaired	- Selection/adaptation/advice on suitable consistencies (IDDSI)
	Adaptive measures	MUCS impaired<br>MDT-PD impaired<br>FEES impaired	- Advice on suitable eating/drinking aids

FDA = Frenchay Dysarthria Assessment; MDT-PD = Munich Dysphagia Test- Parkinson's Disease; MUCSS = Munich-Copenhagen Swallowing Screen; FEES = fiberoptic endoscopic evaluation of swallowing; IDDSI = International Dysphagia Diet Standardisation Initiative.

MeDeMSA Care speech therapy exercise catalogue

1. Dysarthria 5
Posture 5
Breathing 6
Oral motor skills/facial expressions 7
Voice 8
Speech articulation 9
Intelligibility 10
Loudness/volume 11
2. Dysphagia 12
Eating behavior 12
Posture 13
Maintenance measures 14
Compensatory measures 15
Dietary measures 16
Adaptive measures 17

1. Dysarthria

Posture

Repeat each exercise three to five times.

Sit on the chair with your back straight, so that your lower back is touching the back of the chair. Keep your feet hip-width apart with good contact to the floor. Let your shoulders hang loosely. Try to make a long neck as if your head is being pulled upwards by a thread. You should return to this optimal position after each of the exercises below.

1. Turn your head slowly to the right, slowly back to the center and then slowly to the left side - feel the stretch.
2. Tilt your right ear towards your right shoulder, then slowly move your head back to the center. Tilt your left ear towards your left shoulder, then move your head back to the center.
3. Pull your shoulders towards your ears - breathe in and breathe out three times, drop your shoulders during the last expiration.
4. Perform shoulder circles (on both sides, one side, forwards, backwards).
5. Swing your upper body alternately from the left to the right and forward - backward.
6. Move your chin to the chest and stretch your neck (avoid a “turtle” posture).
7. Make yourself very small and straighten up again.
8. Imagine you are picking fruit and alternately stretch your arms upwards.

1. Dysarthria

Breathing

Repeat each exercise three to five times.

Notice: On inspiration: The belly becomes large; the shoulders remain relaxed and do not rise. During expiration: The abdomen becomes small.

1. **Inhale**, hold your breath (5 sec), and exhale on "fffffffff" for as long as possible.
2. **Diaphragm activation:** Place your hand on your stomach and feel the movement.

   Sniff three times in a row

   Pronunciate 3x SCH_SCH_SCHHHH

   Pronunciate 3x S_S_Ssssss

   Pronunciate 3x F_F_Ffffff

   Breathe in, as if you were **smelling** a **soothing fragrance.** Breathe deeply into your belly and consciously perceive this breathing.
   As you **exhale**, imagine that you are **blowing out** a **candle to the notes "SCH", "S", "F" and "F".**
3. **Lip “brake”:** Breathe out through loosely pursed lips, slowly and calmly. Do not press: No breathing noise is produced.
4. **Bubbling exercise**: For this exercise, you need a half-filled water jug. Now take a chewing tube (1 cm in diameter), place it between your incisors and the tip of your tongue (approx. 1.5 cm in your mouth) and bubble a "U" with a relaxed voice. The water should bubble slightly. Vary the exercise by blowing different rhythms and songs into the tube.

1. Dysarthria

Oral motor skills/facial expressions

Repeat each exercise three to five times.

1. Raise your eyebrows, look completely astonished and relax again.
2. Draw your eyebrows together, look angry.
3. Wrinkle your nose and relax it again.
4. Inflate both cheeks, hold the air briefly and then suck in both cheeks.
5. Pursing the lips (kissing mouth) alternating with spreading the lips (smiling with closed or open mouth) as in U-I-O-E.
6. Move the tongue to the right and left corner of the mouth, then stretch it to the tip of the nose and then to the chin.
7. Let your tongue circle between your lips.
8. Click your tongue.
9. Insert a cork between your teeth and hold it loosely for a while.
10. Practice chewing movements (jaw movements to the right, left and circling) in isolation or chewing gum.
11. Blowing exercises: Blow out candles, soap bubbles, suck up pieces of paper with a straw and blow them into a glass.

1. Dysarthria

Voice

Repeat each exercise three to five times.

1. Humming: Tap your sternum with the flat of your hand and hum the letter M.
   Move your hand to the left and right towards your shoulders.
   If possible, stay on one note and stop immediately if your voice creaks.
2. Chewing sound: Close your lips gently and imagine that you are pleasantly chewing your favorite food.
   As you do so, make a casual "Mmmm" or "Mjam" sound.
3. Speak an "Aaaa" with your mouth wide open and try to hold it as **long and loud** as possible.
   Then vary with other vowels such as "Eeee; Iii; Oooo; Uuuu;...."
4. Glide onto a breathing arc of “AaaaUuuuuIiiiiEeeeOooo”.
5. Bouncing exercise: Try to bounce a ball on the floor while saying “pa-pe-pi-po-pu...; ta-te-ti-to-tu...; ka-ke-ki-ko-ku-....”
6. Count in rows: Count from one to five, getting louder and louder; then count backwards, with 5 being as loud as possible and 1 being as quiet as possible (whispering). This exercise can be varied as desired. For example, you count and say every second, third or fourth number out loud.
   You can repeat the same exercise with the days of the week, months, etc.
7. Let the sound "u" slide from the top to the bottom or from the bottom to the top like on a rollercoaster ride.
8. Try to sing "LA"; "MI"; "DO" on three ascending and then descending notes.
9. Repeat sentences with different emphasis (e.g.: **The children** are playing in the garden. The children are **playing** in the garden. The children are playing **in the garden.**).
10. Sing along a song of your choice.

1. Dysarthria

Speech articulation

Repeat each exercise three to five times.

1. Try yawning an "A" in preparation for speaking.
2. Flap your lips like a horse. Finally, click your tongue a few times.
3. Choose different consonant-vowel combinations and repeat them clearly several times, first slowly, then quickly:

   FA FE FI FO FU WA WE WI WO WU BLA BLE BLI BLO BLU DA DE DI DO DU GA GE GI GO GU PA PE PI PO PU TA TE TI TO TU KA KE KI KO KU PA TA KA PO TO KO PUTUKU TEK TIK TOK TUK PAK PEK PIK POK PUK KAK KEK KIK KOK KUK KLAT KLET KLIT KLOT KLUT PLAT PLET PLIT PLIT PLOT PLUT KLAK KLEK KLIK KLIK KLOK KLU

4. Intentionally read words that you find difficult to pronounce out loud and make sure you pronounce them clearly.
   E.g., for German-speaking individuals: Fliegenklatsche, Narzissenstrauch, Rotweinflasche, Tannenbaumschmuck, Vogelscheuche, Siebenschläfer, Maskottchen, Handwerkskammer, Gesprächspartner, etc.
5. Repeat various tongue twisters as clearly, accurately and yet as quickly as possible.
   E.g., for German-speaking individuals: In Ulm und um Ulm und um Ulm herum; Blaukraut bleibt Blaukraut und Brautkleid bleibt Brautkleid, etc.
6. Speak with a cork: Read aloud words/text of your choice with a cork between your lips. Then do it without the cork and compare before and after.
7. Sing songs out loud or talk to friends on the phone.

1. Dysarthria

Intelligibility

Repeat each exercise three to five times.

1. Say difficult words on individual syllables. Tap in rhythm if possible.
   Mon-day; Tues-day; Ja-nu-ary; Fe-bru-ary; mir-ror cab-inet
2. Speak individual sentences or texts rhythmically (with the help of a metronome app).
   E.g., for German-speaking individuals: Was / ha / ben / wir / ge / lacht; Heut / ist / ein / schö / ner / Tag; Morgen / morgen/ nur/ nicht / heute / sagen / alle/ faulen / Leute.; etc.
3. Speak words out louder and louder. Start quietly and gradually increase the volume.
   Nanu Nanu Nanu Nanu Nanu   Achso Achso Achso Achso Achso   Come in Come in Come in Come in
   Attention Attention Attention Attention   Go away, go away, go away   Attention, here we go! Watch out, it's about to start!
4. Shout out the following words as loud as you can:
   Hey!   Ouch!   Oh dear!   Stop!   Watch out!   I see!   You rascal!
5. Read a newspaper text or a poem to your partner with deliberate pauses, paying particular attention that your words are well understood.
6. Read newspaper texts and poems with a cork between your lips (as clearly as possible). Then read the text without the cork. Ask your partner about the clarity of the text.

1. Dysarthria

Loudness/volume

Repeat each exercise three to five times.

1. Take a good breath and say a **loud** and clear "Aaaaaaaa", holding the sound for as long as possible
2. Please speak out very loudly:

   Run! Hey! Ouch! Oh dear! Stop! Watch out! I see! You rascal!

   Stop Watch out! That hurts!
3. Think of five sentences relevant to your daily life that you would like to practice OUT LOUD:
   a. E.g., "Honey, come here for a while!"
   b. "I need to go to the toilet!" ....

   ...and practice these sentences 10 times/day in a row.
4. Read a text aloud every day (film yourself with your smartphone to compare the effect of repeated training).
5. Read a newspaper text or a poem to your partner with deliberate pauses, pay particular attention to the volume of your voice.

2. Dysphagia

Eating behavior

General recommendations for eating:

1. Only eat/drink in the seated position, not while lying flat! At best, sit at a table with a chair or wheelchair. Your upper body should be slightly bent forward and your chin looks downward. If this is not possible for you, at least eat with the upper body in a raised position.
2. If you get tired quickly, it is better to eat small meals several times a day than large amounts three times a day.
3. Ensure a quiet environment during the meal.
4. Eat and drink in small sips and keep your chin to your chest, especially when swallowing.
5. Clear your throat, cough and/or swallow your saliva, if your voice sounds slurred.
6. Reduce the intake of food with troublesome consistency (e.g., rice, cookies, mixed consistency) or process your food accordingly (thicken liquids, puree food, no mixed consistencies).
7. Reduce dairy products intake as they stimulate mucus formation.
8. Please only crush medication after consulting your doctor! If necessary, swallow them with fruit puree or a sip of water.
9. Ensure good oral hygiene.

2. Dysphagia

Posture

Repeat each exercise three to five times.

1. Turn your head slowly to the right, slowly back to the center and then slowly to the left side - feel the stretch.
2. Tilt your right ear towards your right shoulder, then slowly move your head back to the center.
   Tilt your left ear towards your left shoulder, then move your head back to the center.
3. Pull your shoulders towards your ears - breathe in and breathe out three times, drop your shoulders during the last expiration.
4. Perform shoulder circles (on both sides, one side, forwards, backwards).
5. Swing your upper body alternately from the left to the right and forward - backward.
6. Move your chin to the chest and stretch your neck (avoid a “turtle” posture).
7. Make yourself very small and straighten up again.
8. Imagine you are picking fruit and alternately stretch your arms upwards.

2. Dysphagia

Maintenance measures

Repeat each exercise three to five times.

1. Put a spatula between your lips and hold it for a few minutes (e.g., while listening to a song).
2. Take a button (max. 2.5 cm) attached to a 20 cm long thread. Clench your teeth, place the button on your closed incisors and hold it with your closed lips. Pull the thread forward and try to hold the button with your lips.
3. Pull your cheeks slightly outwards with your finger and try to build up resistance by tensing your cheek.
4. Suck your tongue against the roof of your mouth. Keep your tongue sucked in for a few seconds before letting it snap down with a loud bang.
5. Slide your tongue between your incisors and hold it in place with your teeth. Swallow your saliva in this position.
6. Push your tongue out of your mouth as far as possible and then pull it as far back into your throat as possible, as if you were yawning or gargling.
7. Carry out the following blowing exercises: Blow out candles, blow soap bubbles, use a straw to suck up shreds of paper and transport them into a glass.
8. Adjust your tongue as if you were saying a "K" and hold this position or speak words beginning with "K" forcefully.
9. Sing an "I" as high as possible to keep your larynx elevated.
10. Sit at a table with your elbows propped up and press your forehead into the palms of your hands. Hold this tension for a few seconds.
11. Press your palms against each other and say a quick and short "A".
12. Lie on your back. Raise your head against your chest and look down at your feet. Hold this position as long as you can, ideally for one minute.

2. Dysphagia

Compensatory measures

Depending on the problem, different methods can be used to make swallowing easier. This must be tried out and adapted individually.
Below you will find some compensatory measures. Adopt an appropriate posture (see general recommendations on eating behavior above).

1. It can be helpful for the chin to touch the chest when swallowing.
2. When swallowing, try to use your mouth and throat muscles very forcefully and swallow hard (forceful swallowing).
3. Turning your head to the left or to the right may also make swallowing easier (head rotation).
4. Press the back of your tongue against the roof of your mouth when swallowing (imagine you are saying a "K").
5. Swallow the food bolus entirely and clear your throat after each swallowing (and maybe swallow empty).
6. Consciously hold your breath while swallowing, clear your throat or cough immediately after swallowing, but without breathing in between.

2. Dysphagia

Dietary measures

1. Adjust your diet if you find a particular food difficult to chew, your tongue feels weak and/or you get tired while eating. If necessary, switch from normal to soft food (e.g., bread without crust, pasta, very soft meat, etc.) or even to pureed food (e.g., pureed vegetables, smoothie, apple sauce, etc.).
2. If you often have problems with liquids and soups, you can thicken them with natural (honey, nectar) or artificial thickeners (i.e., polysaccarides)
3. It is sometimes advisable to separate consistencies, i.e., drink *after* eating rather *than during,* and avoid mixed consistencies (e.g., pasta soup, grapes, mandarins).
4. Adhere to the prescribed times of your medication schedule. If you have difficulty swallowing the medication, try with a teaspoon of apple sauce or split the tablets, if your doctor has clarified that this is possible (some tablets are designed to release their compound properly only if they remain intact).

2. Dysphagia

Adaptive measures

1. If you frequently choke while drinking, you can thicken liquids with natural (honey, nectar) or artificial thickeners (e.g., polysaccharides).
2. There are also various cups (e.g., with a nose cut-out) and drinking aids (straw with reflux stop) that make drinking easier in everyday life. There are also plates with raised edges and cutlery with thickened handles, which can ease eating if you have motor difficulties.
3. Drink cold or hot liquids. Avoid warm food/drinks, as they are harder to feel.
